# Potential therapeutic targets of gastric cancer explored under endogenous network modeling of clinical data

**DOI:** 10.1038/s41598-024-63812-3

**Published:** 2024-06-07

**Authors:** Xile Zhang, Yong-Cong Chen, Mengchao Yao, Ruiqi Xiong, Bingya Liu, Xiaomei Zhu, Ping Ao

**Affiliations:** 1 Center for Quantitative Life Sciences and Physics Department, Shanghai University, Shanghai, 200444 China; 2 Department of General Surgery, Shanghai Institute of Digestive Surgery, Shanghai Key Laboratory of Gastric Cancer, Ruijin Hospital, Shanghai Jiao Tong University School of Medicine, Shanghai, 200025 China; 3 Shanghai Key Laboratory of Modern Optical Systems, School of Optoelectronic Information and Computer Engineering, University of Shanghai for Science and Technology, Shanghai, 200093 China; 4 School of Biomedical Engineering, Sichuan University, Chengdu, 610065 China

**Keywords:** Biophysics, Cancer, Drug discovery, Systems biology

## Abstract

Improvement in the survival rate of gastric cancer, a prevalent global malignancy and the leading cause of cancer-related mortality calls for more avenues in molecular therapy. This work aims to comprehend drug resistance and explore multiple-drug combinations for enhanced therapeutic treatment. An endogenous network modeling clinic data with core gastric cancer molecules, functional modules, and pathways is constructed, which is then transformed into dynamics equations for *in-silicon* studies. Principal component analysis, hierarchical clustering, and K-means clustering are utilized to map the attractor domains of the stochastic model to the normal and pathological phenotypes identified from the clinical data. The analyses demonstrate gastric cancer as a cluster of stable states emerging within the stochastic dynamics and elucidate the cause of resistance to anti-VEGF monotherapy in cancer treatment as the limitation of the single pathway in preventing cancer progression. The feasibility of multiple objectives of therapy targeting specified molecules and/or pathways is explored. This study verifies the rationality of the platform of endogenous network modeling, which contributes to the development of cross-functional multi-target combinations in clinical trials.

## Introduction

Gastric cancer (GC) is a leading cause of cancer-related deaths globally^1,2^. Its occurrence, development, and prognosis are influenced by various complex factors and regulatory/signaling pathways. Established risk factors for GC include Helicobacter pylori infection, family history, medication, obesity, alcohol consumption, smoking, radiation, and dietary factors^3^. Despite recent advancements in GC prevention, diagnosis, and treatment, early detection rates remain low, and the overall survival for advanced GC is only one year with a poor prognosis^4–6^. This can be attributed, in part, to the extensive heterogeneity of GC, both morphologically and molecularly, as evidenced by numerous histological and molecular classifications^6–12^. Importantly, this heterogeneity occurs not only between patients but also within the same tumor (inter- and intratumor heterogeneity)^6^.

The study of combination molecular targeted therapies requires improved risk models that integrate genetic, lifestyle, and environmental factors^13,14^, which require a comprehensive understanding at the molecular level. Although recent high-throughput studies have provided vast amounts of molecular data with clinical relevance, there is still a general need for prognostic, mechanistic, and predictive systems to identify novel therapeutic targets^15–17^. Additionally, some studies suggest that gastric cancer subtypes may be too robust to be disrupted^18,19^, and experimental evidence supports the existence of high-dimensional attractors and functional robustness in gene expression patterns^20,21^. A collective model based on causal relationships is highly desirable, as it can serve as a “dry laboratory” to simulate carcinogenesis, metastasis, and cancer treatment efficacy. The concept of collective emergence in biology dates back to Waddington, Delbruck, Monod, Jacob, Hinshelwood, and Kauffman^22–27^.

The above argument aligns with the core concept^28^ that cancer corresponds to an inherent state(s) formed by delicate molecular-cell interaction networks. The endogenous network, a causal, quantitative, and simplified system developed in a series of previous works^28–30^, emphasizes the molecular mechanisms behind the disease. The network assembles the collective effects of interactions between molecular/cellular factors in the form of steady states, which include both normal physiological and cancer subtypes. Carcinogenesis is understood as a transition from a normal state to a cancer state. The methodology has been systematically applied to a number of cancerous diseases, including liver cancer^31^, prostate cancer^32^, gastric cancer heterogeneity^33^, leukemia^34^, osteoporosis^35^, colorectal cancer^36^, hematopoietic lineage^37^, and others.

Genetics and epigenetics research have identified numerous genes and molecular pathways that may play a causal role in gastric cancer (GC). This understanding has been further enhanced by large-scale molecular analysis, which has revealed hundreds of differentially expressed molecules in GC and its progression^38–40^. However, in the context of the endogenous network, we recognize the importance of considering genes that may not have a direct relationship with cancer pathology. In addition to well-known oncogenic molecules and pathways such as p53, Myc, Wnt, and NF-kB, we also include molecules that are crucial for embryonic development, particularly those related to the gastrointestinal (GI) tract and mesenchymal lineage. These elements are based on molecular biology and biochemical low-throughput experiments that investigate intracellular and extracellular signaling and other molecular interactions under normal physiological conditions.

In this study, we propose the incorporation of an additional layer of regulation that dynamically adjusts the relaxation time scale on each node of the network. By simulating the dynamics in silico, we identify robust attractor domains and the related elements that coordinate the activity of core regulatory molecules. From the steady-state distributions, we can classify heterogeneous clusters which offers the basis for improvement on the model. Namely, we fine-turn the network by adding nodes or adjusting interaction relationships with a trial-and-error approach towards matching the landscape found in the primary clinical data (collected from the Shanghai Key Laboratory of Gastric Cancer), Part A of SI details the process. Further validation of the model was conducted using gastric cancer data from the TCGA database.

The added regulation allows “dry” search for potential paths to reduce cancer domains, which is the primary objective of our research. We explore the potential and feasibility of targeting specific molecules and/or pathways for drug therapy based on our clinically matched model, to guide for improving the efficiency of gastric cancer treatment. Ultimately, this approach may provide valuable insights for the development of multi-target combinations across functional blocks in clinical trials.

## Materials and methods

### Network construction

**Figure 1:**
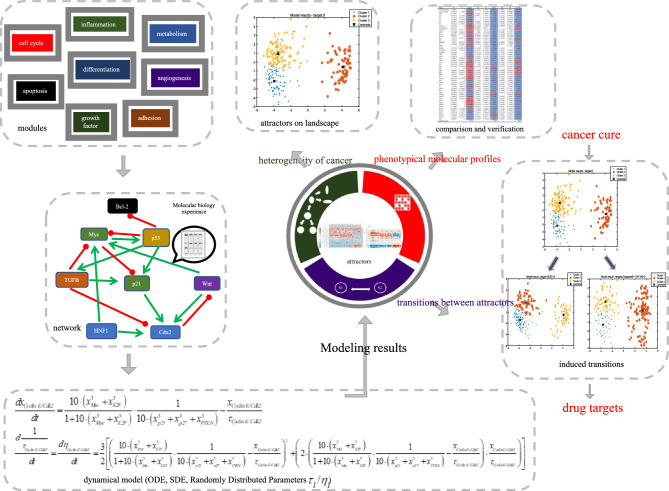
Schematics of endogenous molecular–cellular network construction and modeling (Created in Microsoft PowerPoint). Interactions between molecules are obtained from the literature, with priority given to interactions supported by solid biochemical foundations. Continuously fine-tuning the network notes using real gastric tumor microarray data collected from collaborators at the Shanghai Key Laboratory of GC.

**Figure 2:**
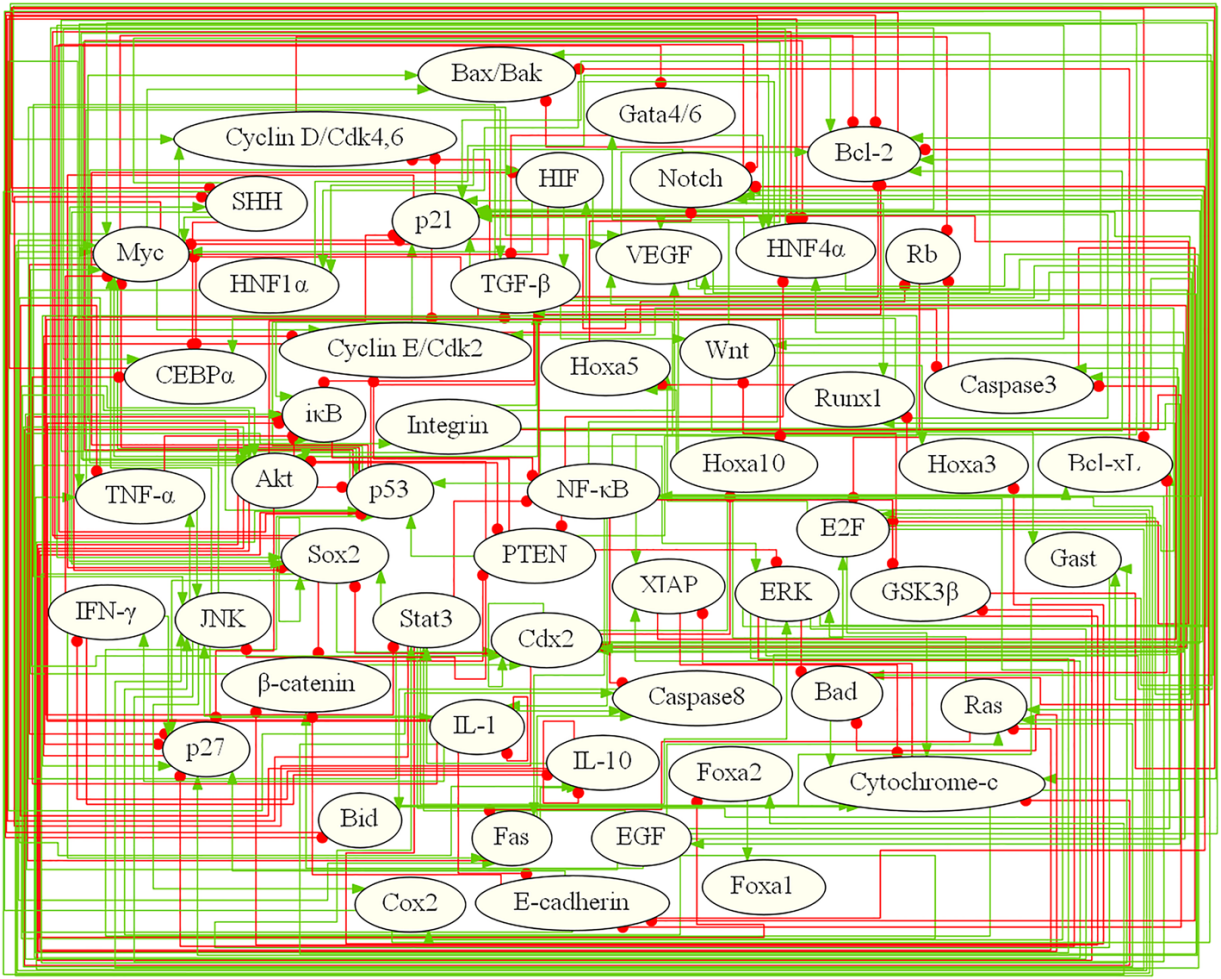
Core Endogenous Network of Gastric Cancer (Created in MATLAB R2023b). The figure illustrates the core molecular factors involved in gastric cancer, represented by ellipses. The lines connecting the ellipses depict the interactions between these factors. Promoting relationships are indicated by green arrow lines, while inhibitory relationships are represented by red lines ending with a solid circle. The core network comprises a total of 55 factors and 253 interaction relationships.

The construction of an endogenous molecular-cellular network, as depicted in Fig. 1, constitutes a fundamental component of gastric cancer (GC) research. Pathways such as Notch, SHH, and Wnt signaling pathways are represented by nodes in the network, with the cascading effects within these pathways also considered. Similarly, the activities of EGF and VEGF pathways are included rather than the expression levels of these proteins. Feedback loops of growth factors are assumed to be self-contained within tumor tissues. To simplify the model, only representative molecules of specific functional modules are included. For instance, in the cell cycle regulation module, factors such as Cyclin E and Cyclin D are included, totaling eight factors. The nodes and edges representing molecules and pathways in the GC network can be found in Table S1 of the SI, along with their interactions listed in Table S2. The model incorporates cell cycle, apoptosis, growth factor signaling, cell adhesion, metabolism, inflammation, and differentiation. Figure 2 illustrates the topological structure of the GC core network, consisting of 55 factors and 253 pairs of interaction relationships.

The construction process involved several steps, which are summarized below by academic writing conventions. Initially, a minimal core network was established to represent the regulation of fundamental cellular functions such as cell cycle, apoptosis, and stress response, drawing upon previous cancer models^31–36^ (see Fig. 1). Subsequently, additional molecules specific to gastrointestinal (GI) tract development and functions, including transcription factors Sox2, Cdx2, and HNF1α, were integrated into the core network. The interactions between molecules were derived from the literature, with a focus on those supported by molecular biology experiments.

Adding nodes or modulating interaction relationships to match the clinical gastric tumor microarray data obtained from our collaborators at the Shanghai Key Laboratory of Gastric Cancer, Ruijin Hospital Affiliated with Shanghai Jiao Tong University School of Medicine (refer to Part A of the SI). Feedback loops associated with inflammation and hematopoiesis were also incorporated. The network was described by dynamical differential equations, which are detailed in Part B of the SI. This formulation enabled the computation of attractor domains.

Notably, to account for the diverse time scales associated with the network nodes and their associated bioprocesses, a new regulatory layer was introduced. This layer allowed for variable degradation times, which self-adjusted towards stable phase spaces. Importantly, the attractors of the network dynamics corresponded to specific GC subtypes as well as the normal gastric phenotype, representing an advancement beyond previous studies. Finally, with the emergence of future knowledge, the network can be expanded and optimized, allowing for a more comprehensive description of the characteristics of gastric cancer.

### Quantitative analysis

The dynamics of the GC network are derived based on the methodology proposed in previous studies^28,30^, with the inclusion of an additional layer of auto-regulation. A coarse-grained modeling approach is employed due to the limited information available on the specific intracellular parameters governing the relationships between the network nodes. The interactions between the nodes are represented by a set of nonlinear activation and/or inhibition rules applied to the agents associated with each node.

Let ${\mathbf{X}} = \left( {x_{1} ,x_{2} , \ldots ,x_{N} } \right)$ denote the complete set of $N$ agents. We consider the time evolution of the concentration/activity of an agent $x_{i}$ under the influence of other agents in the generic form

1 $$ \frac{{{\rm d}x_{i} }}{{{\rm d}t}} = f_{i} \left( {\mathbf{X}} \right) $$

with $a$ denoting literally additional parameters. The total rate of change *f*_*i*_ may be further deposited into production and the degradation rates as

2 $$ f_{i} \left( {\mathbf{X}} \right) = g_{i} \left( {\mathbf{X}} \right) - \frac{{x_{i} }}{{\tau_{{\text{i}}} }} $$

where $g_{i} \left( {\mathbf{X}} \right)$ is some function that captures the impact of facilitation and/or inhibition from other agents on the production rate of agent *i* and *τ*_*i*_ is the degradation time for agent *i*. We follow a standard convention from previous work and choose the nonlinear Hill function for $g_{i} \left( {\mathbf{X}} \right)$, i.e.

3 $$ \frac{{{\rm d}x_{i} }}{{{\rm d}t}} = \frac{{\sum\nolimits_{u} {a_{iu} \cdot x_{u}^{{n_{iu} }} } }}{{1 + \sum\nolimits_{u} {a_{iu} \cdot x_{u}^{{n_{iu} }} } }} \cdot \frac{1}{{1 + \sum\nolimits_{v} {a_{iv} \cdot x_{v}^{{n_{iv} }} } }} - \frac{{x_{i} }}{{\tau_{{\text{i}}} }} $$

Take $n = 3$ for the Hill coefficient, $a = 10$ for the reciprocal of the apparent dissociation constant as the network dynamics is generally insensitive to the choices of these parameters^36^. $\sum\nolimits_{u} {}$ and $\sum\nolimits_{v} {}$ in the above summarize respectively over the agents that promote and inhibit the agent *i* in question. For instance, Cyclin E/Cdk2 is activated Myc and E2F, and inhibited by P21, P27 and PTEN (cf. Table S2 of the SI). The corresponding equation is

4 $$ \frac{{dx_{{{\text{Cyclin E}}/{\text{Cdk2}}}} }}{dt} = \frac{{10 \cdot \left( {x_{{{\text{Myc}}}}^{3} + x_{{{\text{E2F}}}}^{3} } \right)}}{{1 + 10 \cdot \left( {x_{{{\text{Myc}}}}^{3} + x_{{{\text{E2F}}}}^{3} } \right)}} \cdot \frac{1}{{1 + 10 \cdot \left( {x_{{{\text{p21}}}}^{3} + x_{{{\text{p27}}}}^{3} + x_{{{\text{PTEN}}}}^{3} } \right)}} - \frac{{x_{{{\text{Cyclin E}}/{\text{Cdk2}}}} }}{{\tau_{{{\text{Cyclin E}}/{\text{Cdk2}}}} }} $$

The degradation time of proteins, for instance, can exhibit considerable variation^41,42^. In our study, we introduce a random initial distribution for degradation times, which allows for their variability and their role in mitigating imbalances and the impact of random fluctuations. Specifically, we regulate the degradation parameters to promote stability. The temporal change in degradation parameter $\eta_{i} = 1/\tau_{i}$ can be described as follows,

5 $$ \frac{{{\rm d}\eta_{i} }}{{{\rm d}t}} = \frac{3}{2} \cdot \left[ {\left( {\frac{{{\rm d}x_{i} }}{{{\rm d}t}}} \right)^{2} + \left( {2 \cdot \frac{{{\rm d}x_{i} }}{{{\rm d}t}} \cdot \eta_{i} x_{i} } \right)} \right] $$

This set of rules is designed to identify regions within the state and parameter space that yield the most stable steady-state solutions for the aforementioned endogenous network. The inclusion of factor 3/2 on the right-hand side of Eq. (5) serves to adjust the overall scale of the regulatory process to match Eq. (4). For a more comprehensive understanding of the derivation and additional details, please refer to reference 43. A comprehensive list of 110 equations can be found in Part B of the SI. With the improved description of the network dynamic, it better reflects the real-world situation, and the new platform is now capable of monitoring / predicting the efficiency of drug treatments.

### Research involving animal or human rights

No animal and human studies were carried out by the authors for this article.

## Results

### From network model to phenotypes

Gastric cancer (GC) is a complex disease characterized by variations in treatment response and pathology among individuals^44,45^. These variations are reflected in the network modeling as basins of attractors. Transcriptome analyses of tumors have proven effective in identifying distinct subtypes of GC^46^, which can be attributed to the presence of structural domains within the network. To bridge the gap between modeling and phenotypes, common features in GC profiles derived from modeling can be matched with those obtained from clinical measurements and verified previously published microarray data. This comparison involves simulating the trajectories of the system starting from numerous randomly distributed initial points in the state space, which converge towards steady points/attractors. The stability of these fixed points is further analyzed using the Jacobian matrix. These attractors correspond to basins that align with specific cell phenotypes. Within each basin domain, multiple “microscopic” or “degenerate” expression profiles of endogenous agents can be observed^47^. The identification of basins and their associated attractors is achieved by analyzing the distribution of states within the state space.

**Figure 3:**
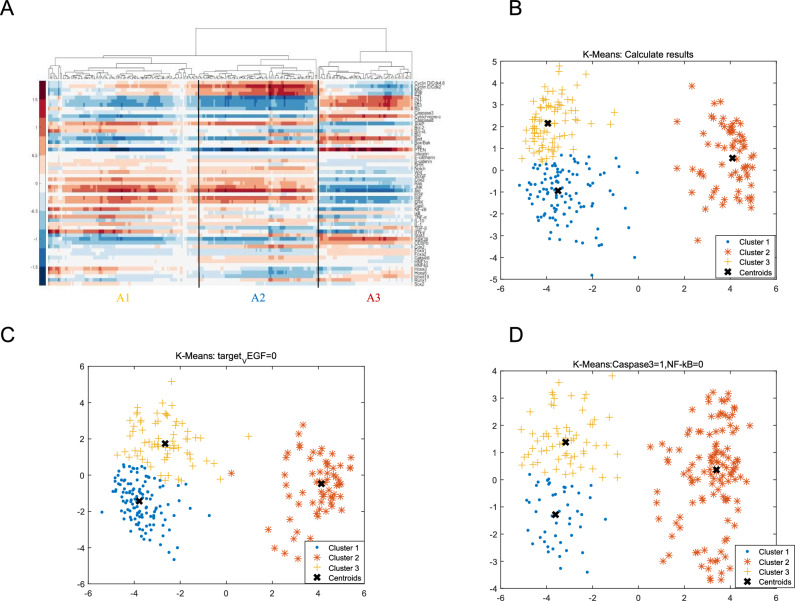
Hierarchical clustering and K-Means clustering (Created in MATLAB R2023b). (**A**) Hierarchical clustering of model’s steady states. The x-axis represents different steady states/samples, while the y-axis represents different genes. (**B**) K-Means clustering of a typical simulation when there is no disturbance to the network agents. (**C**) K-Means clustering of a simulation when the activity of VEGF is downregulated to 0. (**D**) K-Means clustering of a simulation when the activity of Caspase3 is upregulated to 1, and NF-κB is downregulated to 0. (**B**–**D**) The clusters are denoted by the symbols “.”, “*”, and “ + ”, with the centroid of each cluster marked by “ × ”.

In a typical simulation, we were able to obtain some 260 stable points/attractors, each corresponding to a unique phenotypical profile (refer to Part C of the SI for more details). It reflects the individual differences and heterogeneity of gastric cancer, closer to the real situation. Standard data mining techniques were subsequently employed, with Principal Component Analysis (PCA) being utilized for dimensionality reduction and hierarchical clustering (followed by K-means) being utilized for identifying the domains of multiple steady-states. As shown in Fig. 3A, the clustering analysis resulted in the identification of three distinct clusters, which accurately describe both normal physiological and pathological phenotypes. At this stage, the network nodes were carefully examined, for nodes/segments that significantly differ from the experimental data, we can add/adjust interactive relationships between nodes/or nodes towards the latter, which were clinically obtained from gastric cancer and normal samples (collected by some of the authors).

In addition to the basic Euclidean distance clustering, the K-Means algorithm (depicted in Fig. 3B) is utilized to identify the centroid of each cluster. The profiles of these centroid points can be conveniently used to identify the distinctive features of each class (refer to Part C of the SI, Table S3). Furthermore, the common closest state among multiple stable points within each attraction domain can be determined. The number of steady states contained within each cluster and their corresponding percentages are presented in Table 1.

**Table 1:** Distribution of stable points by K-means clustering.

The proportion of each cluster			
Cluster	a1	a2	a3
Points	111	68	81
Proportion	42.69%	26.15%	31.15%

The second row of the table represents the classification of steady states. The third and fourth rows indicate the count and proportion of steady states within each cluster. The size of a domain is characterized by the proportion of shared points among them.

### Comparison and verification

#### Characteristics of GC domains

The anticipated network attractor domains are expected to represent specific biological phenotypes. For more detailed information, please refer to Part C of the SI , specifically Table S3. Domains a1 and a3 are observed to correspond to distinct proliferation phenotypes characterized by the upregulation of Cyclin D/E and E2F. These phenotypes exhibit active signaling pathways such as EGF, HIF, and AKT, which are consistent with epithelial-to-mesenchymal transition, endoderm organ formation, and growth factor-stimulated proliferation^48^. It is worth noting that previous studies have reported high expression levels of VEGF^49^ and NF-κB^50^, indicating the involvement of endothelial cells in angiogenesis and inflammatory responses in the context of GC. One of the basins, classified as an intestinal subtype (I type), exhibits an active Wnt pathway^46^ along with high expression of Cdx2^51^ in basin a1. Conversely, the other basin appears more representative of the diffuse subtype (G type), it exhibits an active Notch pathway^46^ and a high expression of Sox2 in basin a3. Both of these growth states may contribute to cancer progression. While there may be multiple interpretations of the potential phenotypes associated with domains a1 and a3, they exhibit characteristics of both aberrant developmental processes and inflammation. In contrast, attractor domain a2 may represent a cell cycle arrested state and normal cell apoptosis.

We further evaluated the on/off activity or concentration of the agents in each functional module and compared them to known experimental results to summarize their behavior in the three attractor domains (Table 2). In normal gastric epithelium, most cells are in a cell cycle quiescent state, do not resist apoptosis, rely on aerobic metabolism for energy supply, exhibit strong cell adhesion, and do not display inflammation or abnormal angiogenesis. In contrast, GC cells in gastric tissue are in a proliferative state, resist apoptosis, rely on glycolytic metabolism for energy supply, exhibit reduced cell adhesion, and exhibit chronic inflammation and abnormal angiogenesis^52^. The network model predicts a set of subtypes defined by the computed attractor domains a1 and a3, and their combination. Attractor domain a2 may correspond to normal gastric epithelial cells.

**Table 2:** Presents the steady-state and clinical characteristics of the gastric epithelial endogenous network at the functional module level.

	a1	a2	a3	Gastric epithelium	Gastric cancer
Cell Cycle	ON	OFF	ON	OFF	ON
Cell Apoptosis	OFF	OFF	OFF	OFF	OFF
Growth Factors	ON	OFF	ON	OFF	ON|OFF
Cell Adhesion	OFF	ON	OFF	ON	OFF
Angiogenesis	ON	OFF	ON	OFF	ON
Metabolism	ON	OFF	ON	OFF	ON
Inflammation	ON	OFF	ON	OFF	ON
Gastric differentiation	ON	ON	ON|OFF	ON	ON|OFF

Columns 2–4 list the ON/Off states of the functional modules for each of the attractors. It should be noted that certain functional blocks exhibit oscillations and may alternate between ON and OFF states. These oscillating blocks are indicated as ON|OFF in the table. The experimental data-based representation of normal gastric epithelium and gastric cancer is provided in columns 5–6^52^.

#### Model match with clinical samples

To illustrate the match between the model and the clinical data, the simulation results were compared with the experimental data at the molecular level. This comparison was based on data obtained from the research group at Ruijin Hospital, School of Medicine, Shanghai Jiao Tong University, which is affiliated with the present study. The dataset consisted of 111 gastric cancer tissues and 21 non-cancerous gastric tissues, which were analyzed using high-density oligonucleotide microarray analysis. The tumor samples were obtained from gastric subtotal resection surgeries performed on 111 gastric cancer patients, while the normal samples were collected from the gastric mucosa of 21 volunteers who underwent gastroscopy.

**Figure 4:**
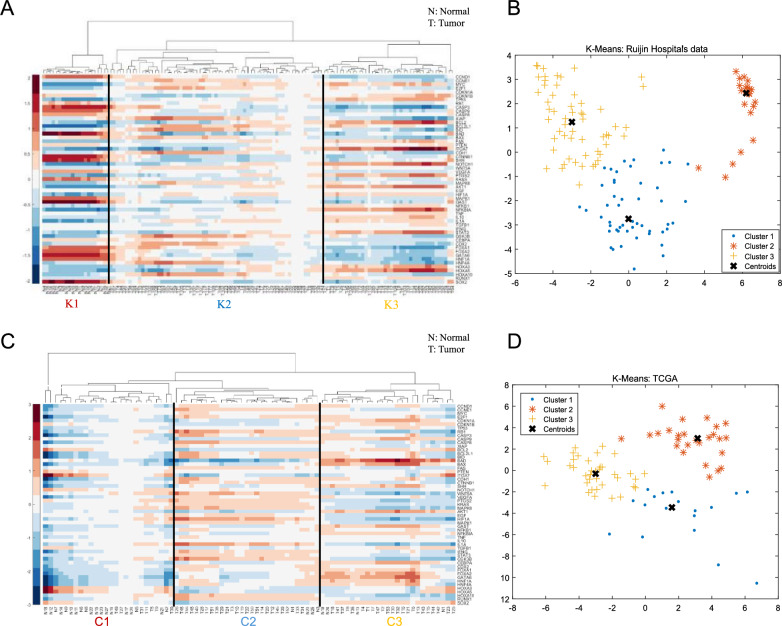
Compare with clinical data from Ruijin Hospital and published microarray data in the TCGA database (Created in MATLAB R2023b). (**A**) Hierarchical clustering of clinical samples. (**B**) K-Means clustering of clinical samples. (**C**) Hierarchical clustering of published microarray data. (**D**) K-Means clustering of published microarray data. (**A**, **C**) The x-axis represents different steady states/samples, while the y-axis represents different genes. (**B**, **D**) The clusters are denoted by the symbols “.”, “*”, and “+”, with the centroid of each cluster marked by “×”.

To analyze the microarray data, we employed the same set of statistical tools, namely principal component analysis (PCA), hierarchical clustering, and K-means clustering. The details of these analyses can be found in Part C of the SI. Using unsupervised hierarchical clustering, all samples from Ruijin Hospital were grouped into three clusters. The normal samples formed one distinct cluster, while the abnormal tumor samples were divided into two clusters, as shown in Fig. 4A. This clustering result is consistent with the *in-silico* findings, indicating that the tumor samples can be classified into two different subtypes. The K-means clustering (Fig. 4B) approach was used to determine the expression profiles for the center points of the identified domains. These expression profiles can be found in Table S4, Part C of the SI.

#### Comparison with TCGA data

The feasibility of the model is further verified by comparing it with a set of previously published microarray data in the TCGA database (The Cancer Genome Atlas Program (TCGA) − NCI). R package (Bioconductor − TCGAbiolinks) was used to obtain and preprocess TCGA data. The dataset included 55 gastric cancer tissues and 27 non-cancerous gastric tissues. The same method as in the above is employed and some further details of these analyses can be found in Part C of the SI.

Using unsupervised hierarchical clustering, almost all samples are grouped into three clusters. Most of the abnormal tumor samples were divided into two clusters, while the overwhelming majority of normal samples formed one distinct cluster, as shown in Fig. 4C. The accuracy rate is 70.90–90.10% (Part C of the SI, Table S6). This clustering result is consistent with the *in-silico* findings, indicating that the tumor samples can be classified into two different subtypes. Furthermore, the K-means clustering (Fig. 4D) approach was used to determine the expression profiles for the center points of the identified domains. These expression profiles can be found in Table S6, Part C of the SI.

Returning to the network simulation, it was previously mentioned that attractor domain a2 represents the normal gastric phenotype (see profile in Part C of SI, Table S3). It is reasonable to assume that cancer tissues are a heterogeneous combination of attractor domains a1 and a3, which can be compared with real tumor samples. Table S3 provides evidence that HNF4A, the TGF-β pathway, and E-cadherin are characteristic markers of the normal stomach. These markers are consistently downregulated in all abnormal states within domains a1 and a3. Analysis of microarray profiles from patients obtained from Ruijin Hospital at Shanghai Jiao Tong University revealed downregulation of HNF4A expression in gastric cancer (GC) patients (see Part C of SI, Table S5). As expected, the downregulation of HNF4A was accompanied by a decrease in gluconeogenesis, as well as upregulated glycolysis and lipogenesis^53–55^. The microarray results also showed high expression levels of the TGF-β pathway in GC patients. The model predicts the upregulation of NF-κB in GC patients within attractor domains a1 and a3. Additionally, the kinase activities of VEGF were found to be active in GC stem cells, which is consistent with both attractor domains a1 and a3.

The differences between attractor domains a1 and a3 were also reflected in the clinical microarray data, with a1 showing activation of Wnt/β-catenin-Cdx2, while a3 showed activation of Sox2-Shh. Biologically, Wnt/β-catenin-Cdx2, play a crucial role in intestinal differentiation^56–58^, with abnormal expression/activation in intestinalized gastric mucosa and intestinal-type gastric cancer^59,60^. Sox2 plays a dominant role in maintaining the gastric epithelial phenotype^33,61^ and is involved in regulating gastric-specific genes such as pepsinogen and Muc5ac^62,63^. Shh target genes can modulate gastric wall cell functions, such as gastric acid secretion^64^. Clinical data also indicate upregulation of Sox2 in gastric-type GC, while upregulation of Cdx2 in intestinal-type GC^65^. The Wnt/β-catenin-Cdx2 upregulated enhancer a1 may be responsible for maintaining intestinal-type GC cells, whereas the Sox2-Shh upregulated enhancer a3 may be responsible for maintaining gastric-type GC cells.

In summary, the comparison with clinical data and TCGA data demonstrates that the endogenous network constructed in this study effectively captures the difference between cancer and normal phenotypes and identifies different GC types. These findings appear sufficient to prove the feasibility of the model.

### Exploration of therapeutic targets

#### Population of steady state domains

Regarding the endogenous network, biological phenotypes correspond to attractor domains of the nonlinear stochastic dynamics that model the interactions on the molecular-cellular network. These attractors, also known as steady states, are more likely to occur and have a longer residence time in real biological systems. They provide a period of stable homeostasis with a specific molecular profile that determines the underlying phenotype. The size of a domain can be described by its relative proportion among the total number of states. This proportion can be altered by imposing constraints on selected nodes of the network, which corresponds to upregulating or downregulating the corresponding molecules through external induction.

To simulate the effects of drug treatments, “dry experiments” can be conducted by randomly elevating or inhibiting the levels of one or multiple nodes at a time. This simulates the “wet experiments” performed in laboratory settings. In our study, we utilized this approach and found that our computational results can potentially explain the cause of resistance to anti-VEGF monotherapy in cancer treatment. This finding highlights the limitation of targeting a single pathway in cancer progression and suggests potential combinations of targets that can enhance the efficacy of GC treatment.

#### Effects of altering single agent

We initiated our study by simulating single-target VEGF inhibition therapy, where the concentration/activity of VEGF was maintained at zero. Figure 3C depicts the emergence of three domains of attraction from the network. Cluster 2, the normal state, accounts for 26.19% of the total number of steady states, indicating a minimal reduction (− 1.15%) with the percentage of steady states remaining nearly unchanged (compared to the unperturbed result (Tables 1 and 3). Cluster 1 and 3, the abnormal state, accounts for 48.81 and 25.00% of the total number of steady states, one increases, and the other decreases. This indicates that anti-VEGF is effective against some gastric cancer patients and not in others. Over the long term, there is no increase in the percentage of normal states, the probability of the system converging to the normal cell state remains largely the same. This may suggest that anti-VEGF monotherapy is likely to be effective in the short term for only some patients and may lead to resistance in the long term. Clinical trials have demonstrated that it is long-term resistance and recurrence, which supports the credibility of our approach^66–68^ (refer to Part D of SI for the simulation details).

**Table 3:** Population of stable points obtained by K-means clustering.

The proportion of each cluster under VEGF = 0			
Cluster	a1	a2	a3
Points number	123	63	66
Proportion	48.81%	25.00%	26.19%

We conducted a comparative analysis between the computationally derived VEGF = 0 results and a curated dataset sourced from GEO Datasets (GSE160613), encompassing gene expression profiles of xenograft tumors derived from human gastric cancer MKN45 cells subjected to treatment with anti-VEGFR2 and anti-VEGF-A antibodies. The accuracy was 57.3%, significantly higher than the average concordance (~ 25%) of randomly generated numbers with the same dimensions in two columns. Given the inherent experimental variability, we posit that this level of agreement is deemed satisfactory. (Refer to Table S7 in Part C of the SI for further details.)

**Figure 5:**
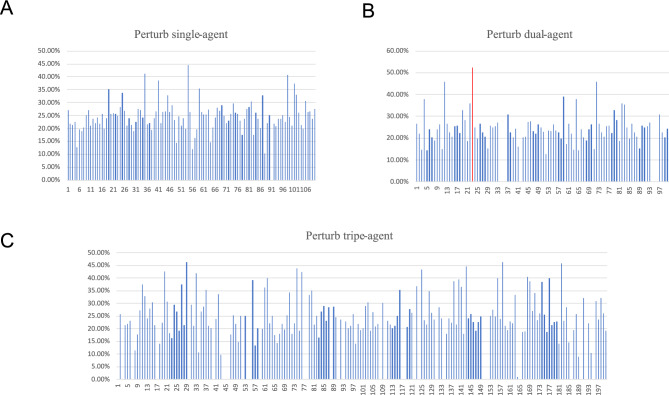
After each perturbation experiment, we recorded the same information as in Tables 1, 3, and 4, and then compiled the percentages of normal states under different perturbation conditions into the above histogram. Each stripe in the histogram represents one perturbation experiment, and the height of the stripe indicates the percentage of normal steady states under that perturbation (Created in Microsoft Excel). (**A**) Perturb single-agent. (**B**) Perturb dual-agent. (**C**) Perturb triple-agent.

The aforementioned analysis can be extended to alternative agents within the network. The network architecture allows for a total of $C_{{{55}}}^{{1}} \cdot C_{{2}}^{{1}} = {110}$ single-agent perturbations. These perturbations were implemented to investigate the potential of effectively preventing GC progression through any individual intervention. Figure 5A illustrates the percentage of stable points within the attractor basins of normal cells for each single-factor intervention experiment. The findings indicate that the proportion of stable states within the attractor basins of normal cells resulting from individual agents perturbing the network is below 45%.

It is evident that exclusively targeting a single agent proves to be ineffective in preventing GC progression. The discriminatory nature of cancer is governed by signaling pathways that exhibit partial redundancy. Consequently, targeted therapies that inhibit a core pathway in the tumor do not completely abolish its functions, thereby allowing some residual activities to persist. It is highly likely that these cells or their progeny will adapt to the selective pressure imposed by the applied therapy^52^, a critical aspect that is duly captured in the computational modeling.

#### Perturbing with dual and triple agents

Combination therapy involving multiple drugs is necessary to advance the efficacy of gastric cancer treatment. In this study, we preliminarily investigate the potential of enhancing treatment outcomes through dual and triple targets therapy approaches. We randomly perturb the network 100 times using dual agents to cover a large portion of combinations, but only 200 times for triple agents due to the limit on computational resources.

For dual-target experiments, the distribution of stable points is depicted in Fig. 3D, and a detailed breakdown is presented in Table 4. Figure 5B illustrates the percentage of stable points within the attractor basins of normal cells following each dual-agent intervention experiment. Notably, one perturbation involving the activation of Caspase3 and inhibition of NF-κB (Caspase3 = 1, NF-κB = 0) resulted in an attractor basin percentage of normal states reaching 52.42%.

**Table 4:** Distribution of stable points obtained by K-means clustering.

The proportion of each cluster(cure:Caspase3 = 1; NF-κB = 0)			
Cluster	a1	a2	a3
Points number	47	61	119
Proportion	20.70%	26.87%	52.42%

For triple-target experiments, the simulation results are presented in Fig. 5C. Among these 200 runs, the highest percentage of stable points within the attractor basins of normal cells was found to be 46.15%, lower than activating Caspase3 and inhibiting NF-κB (52.42%).

In short, targeting a single agent alone does not seem to be the best way to prevent GC progression, and the effect of targeted therapy for gastric cancer may not be proportional to the number of targets, the crucial points that are reflected in the modeling. The simulation results also show that the optimal intervention method is activation of Caspase3 and inhibition of NF-κB (Caspase3 = 1, NF-κB = 0). In terms of cancer characteristics and molecular interactions, the ability of tumor cells to evade apoptosis is a well-known hallmark of cancer. Moreover, dysregulation of apoptotic pathways provides a survival advantage to cells, allowing cancer cells to develop resistance against drugs with diverse functions and/or structures^52,69^. Caspase3, an enzyme known as the “executioner” of cell death, plays a critical role in apoptotic execution, and its activation induces cell apoptosis^70,71^. On the other hand, Helicobacter pylori infection is the most significant known risk factor for gastric cancer and serves as a core trigger for complications including chronic gastritis, gastric ulcers, and gastric cancer. Chronic infection and inflammation contribute to cancer development^72,73^. NF-κB activation typically plays a pivotal role in the inflammatory response triggered by infection and injury^74,75^, and inhibiting NF-κB can mitigate cellular inflammatory responses. Based on these considerations, we can conclude that activating Caspase3 and inhibiting NF-κB represents a promising combination of target interventions that can be further developed as an effective approach against gastric cancer.

## Discussion

The current study investigates potential therapeutic targets for gastric cancer by analyzing the steady-state structure of normal physiological and abnormal pathological phenotypes within a dynamical system. Instead of relying solely on statistical analysis of large datasets, we construct a core endogenous network of gastric cells based on existing biological knowledge and microarray data from tumor samples. The system is quantitatively described using dynamic equations, with an additional layer of regulation introduced to account for the stochastic and biological nature of the system. The model is carefully calibrated by adding or removing functional modules and network nodes to match the phenotypes observed in clinical data.

The model predicts multiple attractors in a typical simulation, which aligns with the significant inter-individual variations observed in cancer data and provides a more realistic representation. The multiplicity of attractors suggests that cells within tumors can occupy different attractors and undergo transitions between them, leading to a mixture of cell phenotypes that may explain the observed non-genetic heterogeneity in tumor tissues. Further statistical analysis of the computed results elucidates the distribution properties of the steady-state structure.

With the successful construction and validation of the core endogenous network, our system serves as a valuable model for conducting “dry experiments” to evaluate the efficacy of molecular targeted therapy for cancer. This approach substantially decreases the costs and search space involved in traditional “wet experiments”, saving time and financial resources. The predictive outcomes of the model can inspire new in vitro/in vivo experiments. Combining the model with experiments can accelerate progress more efficiently.

To illustrate the methodology, we apply a systems dynamics perspective to understand clinical resistance to anti-VEGF monotherapy, and the necessity of multi-target combination therapy to combat the progression of gastric tumor cells. Additionally, we explore a potential combination of therapeutic targets for gastric cancer, specifically the simultaneous activation of Caspase3 and inhibition of NF-κB. The next step, the search space will be greatly increased, it would be beneficial to explore additional perturbations and develop optimal intervention strategies. Such efforts are currently underway. As an interim outcome, a useful combination strategy was identified. The effectiveness of the method has been demonstrated, instilling significant confidence in our ability to discover more effective drugs in the future.

The clinical significance of our study may manifest in several key aspects. Designing personalized treatment plans for cancer patients by optimizing the network using individual genomic information and other biological features, and predicting cancer development trends, while enhancing treatment efficacy and reducing the risk of adverse reactions. In case that no clinically available agents, the effective combinations can be restricted to targets that are influenced by clinically available ones. Furthermore, repositioning of drugs that have failed in Phase II or III clinical trials for new combination therapies.

In brief, this work demonstrates that linking stable points of stochastic dynamics to specific biological observations, such as network topology (via simulation of known molecular interactions) and network dynamics (by comparing gene data from Ruijin Hospital and TCGA datasets with predicted attractors), can effectively describe crucial characteristics of gastric cancer. This includes its diversity among individual patients, with a high level of consistency between network predictions and actual observations. The model constructed in this study provides a novel perspective for understanding the occurrence and progression of gastric cancer and offers a qualitative and quantitative theoretical framework for the development of therapeutic targets for this disease.

### Supplementary Information

Supplementary Information 1.

Supplementary Information 2.

Supplementary Information 3.

Supplementary Information 4.

Supplementary Information 5.

## Data Availability

Clinical data that support the findings of this study have been deposited in The Gene Expression Omnibus code GSE54129. The data for anti-VEGFR2 and anti-VEGF-A can be accessed in the GEO database under the code GSE160613. Gastric cancer microarray data previously published were obtained from the TCGA database (The Cancer Genome Atlas Program—NCI). Simulation data are included in the supplementary information files.
