# Supplementary material for: Potential therapeutic targets of gastric cancer explored under endogenous network modeling of clinical data
Source: Sci Rep. 2024 Jun 7;14:13127. doi: 10.1038/s41598-024-63812-3 (PMC11161650; doi:10.1038/s41598-024-63812-3)
Supplement: Supplementary file 1 — Supplementary Information 1. [file 41598_2024_63812_MOESM1_ESM.pdf]

Supplementary Information for
Potential therapeutic targets of gastric cancer explored under endogenous network modeling of clinical data

Xile Zhang[1)], Yong-Cong Chen[1)] *, Mengchao Yao[1)], Ruiqi Xiong[1)],
Bingya Liu[2)], Xiaomei Zhu[3)], Ping Ao[4)]

（[1)] Center for Quantitative Life Sciences & Physics Department, Shanghai University, Shanghai 200444, China;

[2)] Department of General Surgery, Ruijin Hospital, Shanghai Jiao Tong University School of Medicine, Shanghai Institute of Digestive Surgery, Shanghai Key Laboratory of Gastric Cancer, Shanghai 200025, China;

[3)] Shanghai Key Laboratory of Modern Optical Systems, School of Optoelectronic Information and Computer Engineering, University of Shanghai for Science and Technology, Shanghai 200093, China;

[4)] School of Biomedical Engineering, Sichuan University, Chengdu 610065 China）

（Corresponding Author: chenyongcong@shu.edu.cn）

Abstract: This supplementary information consists of four main parts. Part A provides comprehensive information on the nodes included in the core endogenous network of gastric cancer, along with the references supporting the interactions between factors within the network. Part B presents the equations utilized to transform the network from a topological structure to a dynamic system. Part C encompasses the outcomes of a typical steady-state calculation of the network, as well as the microarray data collected from Ruijin Hospital. Detailed descriptions of the processes and outcomes of principal component analysis (PCA), hierarchical clustering, and K-means clustering for both datasets are also provided. Lastly, Part D presents the results of steady-state calculations following interventions using anti-VEGF and inhibiting Caspase3/activating NF-κB. Additionally, the hierarchical clustering results for the steady states in these two scenarios are included. These results elucidate the expression profiles of factors and can be utilized to determine the classification and modular functionality of steady states.

Contents

A. The factors and interactions of the network 2
B. From network topology to dynamical systems 20
C. Analyze steady states and microarray data 29
Steady State Data and Ruijin Hospital Samples 29
Analysis Process for the four Data Sets 29
D. Simulation results after perturb the network. 38

A. The factors and interactions of the network

Table S1 provides a comprehensive overview of the network model, presenting the modules and the factors included in each module. The network comprises a total of 55 factors and encompasses 253 pairs of interaction relationships. This part also offers detailed information on the nodes within the network, including the specific interactions between these nodes, along with the corresponding references supporting these interactions. This comprehensive representation of the network structure serves as a valuable resource for comprehending the intricate interactions and relationships within the gastric cancer network model. Furthermore, Table S2 lists the corresponding references for further exploration and verification.

Table S1 Modules and Factors of the Core Endogenous Network of Gastric Cells

Modules	Factors
Cell Cycle	Cyclin D/Cdk4,6; Cyclin E/Cdk2; Myc; E2F; p21; p27; p53; Rb
Cell Apoptosis	Caspase 3; Caspase9/Cytochrome c; Caspase 8; XIAP; Bcl-2; Bcl-xL; Bid; Bad; Bax/Bak; Fas
Growth Factor	Ras; JNK; EGF; ERK; Gast
Cell Adhesion	Integrin; E-cadherin; β-catenin
Angiogenesis	VEGF; Cox2;
Metabolism	Akt; PTEN; HIF; GSK3β
Inflammation	NF-κB; iκB; TNF-α; IL-10; IL-1; IFN-γ; TGF-β; STAT3
Gastric Differentiation	CEBPα; CDX2; Foxa1; Foxa2; Gata4/6; HNF1α; HNF4α; Hoxa3; Hoxa5; Hoxa10; Runx1; Sox2; Notch; Wnt; SHH

The core endogenous network of gastric cancer is constructed based on the hierarchical structure of cell regulatory networks and feedback from the surrounding environment. We first select important modules responsible for basic cellular functions and modules related to cancer (such as cell cycle, apoptosis, metabolism and stress response, immune response, and other fundamental functional modules and pathways), and then determine the key factors for each module. After agents are selected and integrated, we conducted extensive literature searches on interactions between the agents and carried out rigorous experimental validation to construct the network. It is important to note that network construction is an iterative process. Taking Network1 - the initial version of the network (see Excel - Network1.xlsx) as an example, we applied K-Means clustering to the attractors obtained from Network1 to generate a steady-state landscape graph, as illustrated in Figure S1A. All stable points were mixed without clear classification, which was inconsistent with the clinical data landscape graph from Ruijin Hospital (see Figure 4B in the main text). Furthermore, the metabolism module exhibited high expression in all attractors (see Figure S1B), which contradicted the experimental data. By introducing inhibitory factors of the metabolism module from other elements in the network and conducting multiple iterations, the final version of the network (see Table S2) was obtained. At this stage, K-Means clustering was used to categorize the attractors, resulting in 3 clusters, along with the expression patterns at the molecular level for each cluster, which were then compared with clinical data. The condition for network matching is that the consistency rate at the module level between the constructed model and experimental data should be 100%. Considering the data quality, the consistency rate at the molecular level should be around 70%, not less than 60%.

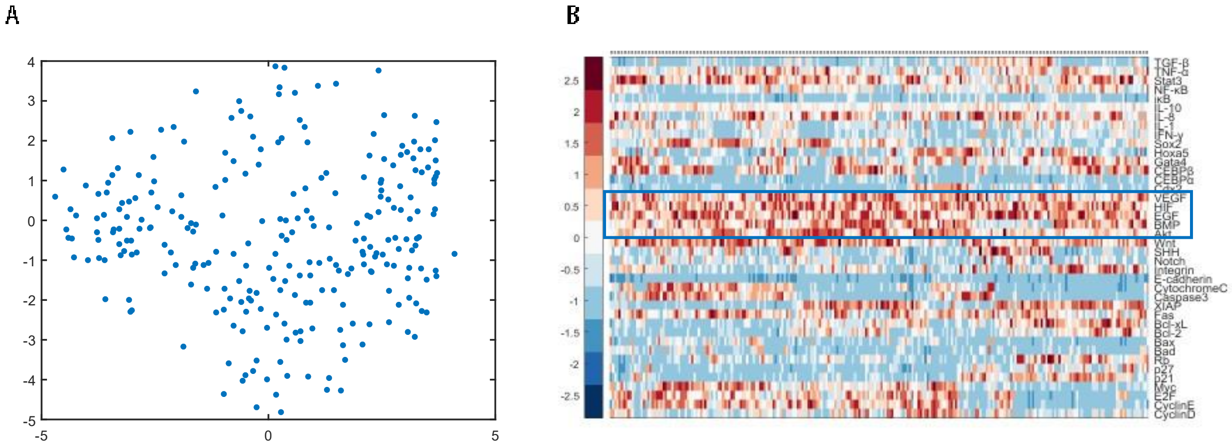

Figure S1: A K-Means clustering of Network1. B HeatMap of Network1, The x-axis represents different stable points, while the y-axis represents different genes.

Table S2 Literature sources for the interactions between factors in the network

Factors	Activator	Inhibitor
Cyclin D/Cdk4,6	Myc[1], EGF[2], Akt[3], Ras[4]	p21[5,6], P53[5], TGF-β[1]
Cyclin E/Cdk2	Myc[1 7], E2F[5]	p21[5,6], p27[5,6], PTEN[5]
Myc	E2F[8,9], Akt[9], ERK[9], JNK[9], β-catenin[10], NF-κB[11], HIF[12], SHH[13]	p53[8], TGF-β[14,15], IFN-γ[12,16-18], p21[19,20]
E2F	E2F[5], Myc[5]	Rb[21,22], p21[6]
p21	p53[23,24], TGF-β[25,26], E2F[27,28], HIF[29], IFN-γ[30-32], ERK[20], Cdx2[33], HNF4α[34], Runx1[35]	Myc[23,36], Akt[6]
p27	PTEN[37], E-cadherin[38], IFN-γ[32,39,40], TGF-β[41], Sox2[42]	Myc[8,43], Akt[44], Cyclin E/Cdk2[45], ERK[46,47]
p53	E2F[48,49], Myc[8,50,51], NF-κB[11], Cytochrome c[52], PTEN[53]	Akt[54,55], Notch[56]
Rb		Cyclin D/Cdk4,6[57], Cyclin E/Cdk2[57], Caspase 3[58]
Caspase 3	Caspase 8[59-63], Cytochrome c[59-64]	p21[65,66], XIAP[67,68]
Cytochrome c	Bad[69], Bax[69,70], Bid[69], E2F[71,72], Myc[73]	Akt[74,75], XIAP[67,68]
Caspase 8	Fas[69], TNF-α[69,76], IL-1[77]	NF-κB[41,78]
XIAP	Akt[79], NF-κB[11,80]	Cytochrome c[67]
Bcl-2	VEGF[81], NF-κB[82], ERK[83], SHH[13], Cox2[84]	p53[85,86], TGF-β[87], Bad[69], Myc[88], Bid[84]
Bcl-x$_L$	EGF[89-91],NF-κB[11,80]	Bad[69], Myc[88]
Bid	Caspase 8[69], P53[84]	Bcl-2[84]
Bad	p53[92]	Akt[93], ERK[94]
Bax/Bak	Myc[88], p53[85,86], Bid[84]	Bcl-2[95], Bcl-x$_L$[96]
Fas	NF-κB[11], IFN-γ[97], Myc[84]	Ras[98]
Ras	EGF[99], VEGF[100], Integrin[54,55,101,102]	p53[103]
JNK	TGF-β[104,105], TNF-α[106], IL-1[107-109], EGF[110], VEGF[100]	PTEN[111]
EGF	Integrin[112]	
ERK	Ras[113], Integrin[101,102], Stat3[114]	PTEN[111]
Gast	Sox2[115], TGF-β[116,117], Wnt[116,117]	
Integrin	EGF[118-120]	PTNE[102]
E-cadherin		Integrin[121], IL-1[122], Caspase 3[123]
β-catenin	EGF[124]	E-cadherin[125], Sox2[126], GSK3β[127]
VEGF	HIF[128], Akt[129], HGF[12], Cox2[130], IL-1[131], Stat3[132], Integrin[112]	
Cox2	NF-κB[11,133,134], JNK[135,136]	
Akt	Ras[113], E-cadherin[137], Integrin[112,138], VEGF[138], EGF[110], TGF-β[87,139]	PTEN[111]
PTEN		Akt[140], NF-κB[141], β-catenin[142]
HIF	Akt[129,143], NF-κB[144,145], Myc[146]	p53[147]
GSK3β		Akt[148], NF-κB[149]
NF-κB	JNK[150]	iκB[11,151], Stat3[152,153]
iκB	NF-κB[11,154,155], ERK[156], JNK[156]	TNF-α[151], IL-1[151,157], Akt[158-160], Fas[161], TGF-β[162]
TNF-α	NF-κB[11], ERK[163], JNK[163]	IL-10[164]
IL-10	TNF-α[165], Fas[166,167]	IFN-γ[168], IL-10[169]
IL-1	NF-κB[146], JNK[156]	IL-1[170-173]
IFN-γ	IL-1[174-176]	IL-10[177]

TGF-β	Integrin[178,179], HIF[180], Hoxa10[181], TNF-α[182,183]	NF-κB[162,184], Gata4/6[185], Sox2[186]
Stat3	IL-10[114], EGF[187,188], Gast[189]	TGF-β[190,191]
CEBPα	HNF4α[192], Runx1[193]	HIF[194], IL-1[195], SHH[196]
Cdx2	β-catenin[197], Cdx2[197], HNF1α[198], HNF4α[199], Wnt[200], Sox2[201]	Stst3[202],TGF-β[203]
Foxa1	Foxa2[204]	
Foxa2	C/EBPα[205], SHH[206]	Akt[207]
Gata4/6	Wnt[208]	Sox2[201,209,210]
HNF1α	HNF4α[211], Notch[212]	C/EBPα[213]
HNF4α	C/EBPα[214], Cdx2[199], E-Cadherin[215], Gata4/6[216], HNF1α[211]	NF-κB[217], p53[218], Sox2[115], Stat3[219]
Hoxa3	Wnt[220]	Stat3[221]
Hoxa5	Cdx2[222];Hoxa10[223]	Runx1[224]
Hoxa10		Cdx2[225],IL-1[226]
Runx1	Cdx2[227], Notch[228]	Hoxa3[229]
Sox2	Stat3[230], SHH[231], Sox2[232], Wnt[233]	p21[234], Cdx2[235]
Notch	NF-κB[236], Cdx2[237,238], Stat3[239]	CEBPα[240], E-cadherin[241], Hoxa5[242]
Wnt	EGF[243], TGF-β[78,244]	GSK3β[245]
SHH	Sox2[246]	Cdx2[247], GSK3β[13]

References

1 Obaya, A. J., Mateyak, M. K. & Sedivy, J. M. Mysterious liaisons: the relationship between c-Myc and the cell cycle. *Oncogene* **18**, 2934-2941 (1999). https://doi.org/10.1038/sj.onc.1202749

2 Tao, Y. *et al.* Nuclear accumulation of epidermal growth factor receptor and acceleration of G1/S stage by Epstein-Barr-encoded oncoprotein latent membrane protein 1. *Experimental Cell Research* **303**, 240-251 (2005). https://doi.org/10.1016/j.yexcr.2004.09.030

3 Oh, S. J. *et al.* Targeting cyclin D-CDK4/6 sensitizes immune-refractory cancer by blocking the SCP3-NANOG axis. *Cancer Research* **78**, 2638-2653 (2018). https://doi.org/10.1158/0008-5472.Can-17-2325

4 Gille, H. & Downward, J. Multiple ras effector pathways contribute to G(1) cell cycle progression. *Journal of Biological Chemistry* **274**, 22033-22040 (1999). https://doi.org/10.1074/jbc.274.31.22033

5 Murray, A. W. & Hunt, T. *The cell cycle: an introduction*. Vol. 3 (Oxford University Press 1993).

6 Sherr, C. J. & Roberts, J. M. CDK inhibitors: positive and negative regulators of G1-phase progression. *Genes & Development* **13**, 1501-1512 (1999). https://doi.org/10.1101/gad.13.12.1501

7 Singh, A. K., Swarnalatha, M. & Kumar, V. c-ETS1 facilitates G1/S-phase transition by up-regulating cyclin E and CDK2 genes and cooperates with hepatitis B virus X protein for their deregulation. *Journal of Biological Chemistry* **286**, 21961-21970 (2011). https://doi.org/10.1074/jbc.M111.238238

8 Dang, C. V. c-Myc target genes involved in cell growth, apoptosis, and metabolism. *Molecular and Cellular Biology* **19**, 1-11 (1999). https://doi.org/10.1128/mcb.19.1.1

9 Zhu, J., Blenis, J. & Yuan, J. Activation of PI3K/Akt and MAPK pathways regulates Myc-mediated transcription by phosphorylating and promoting the degradation of Mad1. *Proceedings of the National Academy of Sciences of the United States of America* **105**, 6584-6589 (2008). https://doi.org/10.1073/pnas.0802785105

10 He, T. C. *et al.* Identification of c-MYC as a target of the APC pathway. *Science* **281**, 1509-1512 (1998). https://doi.org/10.1126/science.281.5382.1509

11 Pahl, H. L. Activators and target genes of Rel/NF-kappaB transcription factors. *Oncogene* **18**, 6853-6866 (1999). https://doi.org/10.1038/sj.onc.1203239

12 Zhang, Y. W., Su, Y., Volpert, O. V. & Vande Woude, G. F. Hepatocyte growth factor/scatter factor mediates angiogenesis through positive VEGF and negative thrombospondin 1 regulation. *Proceedings of the National Academy of Sciences of the United States of America* **100**, 12718-12723 (2003). https://doi.org/10.1073/pnas.2135113100
13 Merchant, J. L. Hedgehog signalling in gut development, physiology and cancer. *Journal of Physiology-London* **590**, 421-432 (2012). https://doi.org/10.1113/jphysiol.2011.220681
14 Chen, C.-R., Kang, Y., Siegel, P. M. & Massagué, J. E2F4/5 and p107 as Smad cofactors linking the TGFβ receptor to c-myc repression. *Cell* **110**, 19-32 (2002).
15 Alexandrow, M. G. & Moses, H. L. Transforming growth factor beta and cell cycle regulation. *Cancer Research* **55**, 1452-1457 (1995).
16 Vairo, G. *et al.* Deregulated c-myc expression overrides IFN gamma-induced macrophage growth arrest. *Oncogene* **10**, 1969-1976 (1995).
17 Ayer, D. E. & Eisenman, R. N. A switch from Myc:Max to Mad:Max heterocomplexes accompanies monocyte/macrophage differentiation. *Genes & Development* **7**, 2110-2119 (1993). https://doi.org/10.1101/gad.7.11.2110
18 Ayer, D. E., Kretzner, L. & Eisenman, R. N. Mad: a heterodimeric partner for Max that antagonizes Myc transcriptional activity. *Cell* **72**, 211-222 (1993). https://doi.org/10.1016/0092-8674(93)90661-9
19 Kitaura, H. *et al.* Reciprocal regulation via protein-protein interaction between c-Myc and p21(cip1/waf1/sdi1) in DNA replication and transcription. *Journal of Biological Chemistry* **275**, 10477-10483 (2000). https://doi.org/10.1074/jbc.275.14.10477
20 Abbas, T. & Dutta, A. p21 in cancer: intricate networks and multiple activities. *Nature Reviews Cancer* **9**, 400-414 (2009). https://doi.org/10.1038/nrc2657
21 Dyson, N. The regulation of E2F by pRB-family proteins. *Genes & Development* **12**, 2245-2262 (1998). https://doi.org/10.1101/gad.12.15.2245
22 Harbour, J. W. & Dean, D. C. The Rb/E2F pathway: expanding roles and emerging paradigms. *Genes & Development* **14**, 2393-2409 (2000). https://doi.org/10.1101/gad.813200
23 Gartel, A. L. & Radhakrishnan, S. K. Lost in transcription: p21 repression, mechanisms, and consequences. *Cancer Research* **65**, 3980-3985 (2005). https://doi.org/10.1158/0008-5472.Can-04-3995
24 el-Deiry, W. S. *et al.* WAF1, a potential mediator of p53 tumor suppression. *Cell* **75**, 817-825 (1993). https://doi.org/10.1016/0092-8674(93)90500-p
25 Datto, M. B. *et al.* Transforming growth factor beta induces the cyclin-dependent kinase inhibitor p21 through a p53-independent mechanism. *Proceedings of the National Academy of Sciences of the United States of America* **92**, 5545-5549 (1995). https://doi.org/10.1073/pnas.92.12.5545
26 Datto, M. B., Yu, Y. & Wang, X. F. Functional analysis of the transforming growth factor beta responsive elements in the WAF1/Cip1/p21 promoter. *Journal of Biological Chemistry* **270**, 28623-28628 (1995). https://doi.org/10.1074/jbc.270.48.28623
27 Hiyama, H., Iavarone, A. & Reeves, S. A. Regulation of the cdk inhibitor p21 gene during cell cycle progression is under the control of the transcription factor E2F. *Oncogene* **16**, 1513-1523 (1998). https://doi.org/10.1038/sj.onc.1201667

28 Radhakrishnan, S. K. *et al.* Constitutive expression of E2F-1 leads to p21-dependent cell cycle arrest in S phase of the cell cycle. *Oncogene* **23**, 4173-4176 (2004). https://doi.org/10.1038/sj.onc.1207571
29 Koshiji, M. *et al.* HIF-1alpha induces cell cycle arrest by functionally counteracting Myc. *Embo Journal* **23**, 1949-1956 (2004). https://doi.org/10.1038/sj.emboj.7600196
30 Xaus, J. *et al.* Interferon gamma induces the expression of p21waf-1 and arrests macrophage cell cycle, preventing induction of apoptosis. *Immunity* **11**, 103-113 (1999). https://doi.org/10.1016/s1074-7613(00)80085-0
31 Kominsky, S. *et al.* IFNgamma inhibition of cell growth in glioblastomas correlates with increased levels of the cyclin dependent kinase inhibitor p21WAF1/CIP1. *Oncogene* **17**, 2973-2979 (1998). https://doi.org/10.1038/sj.onc.1202217
32 Mandal, M., Bandyopadhyay, D., Goepfert, T. M. & Kumar, R. Interferon-induces expression of cyclin-dependent kinase-inhibitors p21WAF1 and p27Kip1 that prevent activation of cyclin-dependent kinase by CDK-activating kinase (CAK). *Oncogene* **16**, 217-225 (1998). https://doi.org/10.1038/sj.onc.1201529
33 Yuasa, Y. Control of gut differentiation and intestinal-type gastric carcinogenesis. *Nature Reviews Cancer* **3**, 592-600 (2003). https://doi.org/10.1038/nrc1141
34 Apte, U., Bell, A., Walesky, C., Edwards, G. & Wolfe, A. Hepatocyte Nuclear Factor 4 alpha (HNF4α) is involved in regulation of hepatocyte proliferation. *The Faseb Journal* **24**, 2362-2362 (2010).
35 Hoi, C. S. *et al.* Runx1 directly promotes proliferation of hair follicle stem cells and epithelial tumor formation in mouse skin. *Molecular and Cellular Biology* **30**, 2518-2536 (2010).
36 Seoane, J., Le, H. V. & Massagué, J. Myc suppression of the p21(Cip1) Cdk inhibitor influences the outcome of the p53 response to DNA damage. *Nature* **419**, 729-734 (2002). https://doi.org/10.1038/nature01119
37 Gottschalk, A. R. *et al.* p27Kip1 is required for PTEN-induced G1 growth arrest. *Cancer Research* **61**, 2105-2111 (2001).
38 St Croix, B. *et al.* E-Cadherin-dependent growth suppression is mediated by the cyclin-dependent kinase inhibitor p27(KIP1). *Journal of Cell Biology* **142**, 557-571 (1998). https://doi.org/10.1083/jcb.142.2.557
39 Harvat, B. L., Seth, P. & Jetten, A. M. The role of p27Kip1 in gamma interferon-mediated growth arrest of mammary epithelial cells and related defects in mammary carcinoma cells. *Oncogene* **14**, 2111-2122 (1997). https://doi.org/10.1038/sj.onc.1201055
40 Matsuoka, M., Nishimoto, I. & Asano, S. Interferon-gamma impairs physiologic downregulation of cyclin-dependent kinase inhibitor, p27Kip1, during G1 phase progression in macrophages. *Experimental Hematology* **27**, 203-209 (1999). https://doi.org/10.1016/s0301-472x(98)00034-4
41 Ciarallo, S. *et al.* Altered p27(Kip1) phosphorylation, localization, and function in human epithelial cells resistant to transforming growth factor beta-mediated G(1) arrest. *Molecular and Cellular Biology* **22**, 2993-3002 (2002). https://doi.org/10.1128/mcb.22.9.2993-3002.2002
42 Otsubo, T., Akiyama, Y., Yanagihara, K. & Yuasa, Y. SOX2 is frequently downregulated in gastric cancers and inhibits cell growth through cell-cycle arrest and apoptosis. *British Journal*

of Cancer **98**, 824-831 (2008). https://doi.org/10.1038/sj.bjc.6604193
43 Slingerland, J. & Pagano, M. Regulation of the cdk inhibitor p27 and its deregulation in cancer. *Journal of Cellular Physiology* **183**, 10-17 (2000). https://doi.org/10.1002/(sici)1097-4652(200004)183:1<10::Aid-jcp2>3.0.Co;2-i
44 Sa, G. & Stacey, D. W. P27 expression is regulated by separate signaling pathways, downstream of Ras, in each cell cycle phase. *Experimental Cell Research* **300**, 427-439 (2004). https://doi.org/10.1016/j.yexcr.2004.07.032
45 Sheaff, R. J., Groudine, M., Gordon, M., Roberts, J. M. & Clurman, B. E. Cyclin E-CDK2 is a regulator of p27Kip1. *Genes & Development* **11**, 1464-1478 (1997). https://doi.org/10.1101/gad.11.11.1464
46 Rivard, N., Boucher, M. J., Asselin, C. & L'Allemain, G. MAP kinase cascade is required for p27 downregulation and S phase entry in fibroblasts and epithelial cells. *American Journal of Physiology-Cell Physiology* **277**, C652-C664 (1999). https://doi.org/10.1152/ajpcell.1999.277.4.C652
47 Quante, M., Varga, J., Wang, T. C. & Greten, F. R. The gastrointestinal tumor microenvironment. *Gastroenterology* **145**, 63-78 (2013). https://doi.org/10.1053/j.gastro.2013.03.052
48 Sherr, C. J. The INK4a/ARF network in tumour suppression. *Nature Reviews Molecular Cell Biology* **2**, 731-737 (2001). https://doi.org/10.1038/35096061
49 Sherr, C. J. & Weber, J. D. The ARF/p53 pathway. *Current opinion in genetics & development* **10**, 94-99 (2000).
50 Evan, G. I. *et al.* Induction of apoptosis in fibroblasts by c-myc protein. *Cell* **69**, 119-128 (1992). https://doi.org/10.1016/0092-8674(92)90123-t
51 Reisman, D., Elkind, N. B., Roy, B., Beamon, J. & Rotter, V. c-Myc trans-activates the p53 promoter through a required downstream CACGTG motif. *Cell Death and Differentiation* **4**, 57-65 (1993).
52 Cregan, S. P., Dawson, V. L. & Slack, R. S. Role of AIF in caspase-dependent and caspase-independent cell death. *Oncogene* **23**, 2785-2796 (2004). https://doi.org/10.1038/sj.onc.1207517
53 Stambolic, V. *et al.* Regulation of PTEN transcription by p53. *Molecular Cell* **8**, 317-325 (2001). https://doi.org/10.1016/s1097-2765(01)00323-9
54 Khwaja, A., Rodriguez-Viciana, P., Wennström, S., Warne, P. H. & Downward, J. Matrix adhesion and Ras transformation both activate a phosphoinositide 3-OH kinase and protein kinase B/Akt cellular survival pathway. *Embo Journal* **16**, 2783-2793 (1997). https://doi.org/10.1093/emboj/16.10.2783
55 Frisch, S. M. & Francis, H. Disruption of epithelial cell-matrix interactions induces apoptosis. *Journal of Cell Biology* **124**, 619-626 (1994). https://doi.org/10.1083/jcb.124.4.619
56 Beverly, L. J., Felsher, D. W. & Capobianco, A. J. Suppression of p53 by Notch in lymphomagenesis: implications for initiation and regression. *Cancer Research* **65**, 7159-7168 (2005). https://doi.org/10.1158/0008-5472.Can-05-1664
57 Weinberg, R. A. The retinoblastoma protein and cell cycle control. *Cell* **81**, 323-330 (1995).
58 Katsuda, K. *et al.* Activation of caspase-3 and cleavage of Rb are associated with p16-mediated

apoptosis in human non-small cell lung cancer cells. *Oncogene* **21**, 2108-2113 (2002). https://doi.org/10.1038/sj.onc.1205272
59 Budihardjo, I., Oliver, H., Lutter, M., Luo, X. & Wang, X. Biochemical pathways of caspase activation during apoptosis. *Annual Review of Cell and Developmental Biology* **15**, 269-290 (1999). https://doi.org/10.1146/annurev.cellbio.15.1.269
60 Chang, H. Y. & Yang, X. Proteases for cell suicide: functions and regulation of caspases. *Microbiology and Molecular Biology Reviews* **64**, 821-846 (2000). https://doi.org/10.1128/mmbr.64.4.821-846.2000
61 Zou, H. *et al.* Regulation of the Apaf-1/caspase-9 apoptosome by caspase-3 and XIAP. *Journal of Biological Chemistry* **278**, 8091-8098 (2003). https://doi.org/10.1074/jbc.M204783200
62 Liu, Z. *et al.* ras Oncogene triggers up-regulation of cIAP2 and XIAP in intestinal epithelial cells: epidermal growth factor receptor-dependent and -independent mechanisms of ras-induced transformation. *Journal of Biological Chemistry* **280**, 37383-37392 (2005). https://doi.org/10.1074/jbc.M503724200
63 Adrain, C. & Martin, S. J. The mitochondrial apoptosome: a killer unleashed by the cytochrome seas. *Trends In Biochemical Sciences* **26**, 390-397 (2001). https://doi.org/10.1016/s0968-0004(01)01844-8
64 Bratton, S. B. *et al.* Recruitment, activation and retention of caspases-9 and -3 by Apaf-1 apoptosome and associated XIAP complexes. *Embo Journal* **20**, 998-1009 (2001). https://doi.org/10.1093/emboj/20.5.998
65 Suzuki, A., Tsutomi, Y., Akahane, K., Araki, T. & Miura, M. Resistance to Fas-mediated apoptosis: activation of caspase 3 is regulated by cell cycle regulator p21WAF1 and IAP gene family ILP. *Oncogene* **17**, 931-939 (1998). https://doi.org/10.1038/sj.onc.1202021
66 Suzuki, A., Tsutomi, Y., Yamamoto, N., Shibutani, T. & Akahane, K. Mitochondrial regulation of cell death: mitochondria are essential for procaspase 3-p21 complex formation to resist Fas-mediated cell death. *Molecular and Cellular Biology* **19**, 3842-3847 (1999). https://doi.org/10.1128/mcb.19.5.3842
67 Deveraux, Q. L. & Reed, J. C. IAP family proteins--suppressors of apoptosis. *Genes & Development* **13**, 239-252 (1999). https://doi.org/10.1101/gad.13.3.239
68 Salvesen, G. S. & Duckett, C. S. IAP proteins: blocking the road to death's door. *Nature Reviews Molecular Cell Biology* **3**, 401-410 (2002). https://doi.org/10.1038/nrm830
69 Igney, F. H. & Krammer, P. H. Death and anti-death: tumour resistance to apoptosis. *Nature Reviews Cancer* **2**, 277-288 (2002). https://doi.org/10.1038/nrc776
70 Arnoult, D. *et al.* Mitochondrial release of AIF and EndoG requires caspase activation downstream of Bax/Bak-mediated permeabilization. *Embo Journal* **22**, 4385-4399 (2003). https://doi.org/10.1093/emboj/cdg423
71 Moroni, M. C. *et al.* Apaf-1 is a transcriptional target for E2F and p53. *Nature Cell Biology* **3**, 552-558 (2001). https://doi.org/10.1038/35078527
72 Furukawa, Y. *et al.* Apaf-1 is a mediator of E2F-1-induced apoptosis. *Journal of Biological Chemistry* **277**, 39760-39768 (2002). https://doi.org/10.1074/jbc.M200805200
73 Juin, P., Hueber, A. O., Littlewood, T. & Evan, G. c-Myc-induced sensitization to apoptosis is mediated through cytochrome c release. *Genes & Development* **13**, 1367-1381 (1999).

https://doi.org/10.1101/gad.13.11.1367
74 Fujita, E. *et al.* Akt phosphorylation site found in human caspase-9 is absent in mouse caspase-9. *Biochemical and Biophysical Research Communications* **264**, 550-555 (1999). https://doi.org/10.1006/bbrc.1999.1387
75 Zhou, H., Li, X. M., Meinkoth, J. & Pittman, R. N. Akt regulates cell survival and apoptosis at a postmitochondrial level. *Journal of Cell Biology* **151**, 483-494 (2000). https://doi.org/10.1083/jcb.151.3.483
76 Gaur, U. & Aggarwal, B. B. Regulation of proliferation, survival and apoptosis by members of the TNF superfamily. *Biochemical Pharmacology* **66**, 1403-1408 (2003).
77 Janssens, S. & Beyaert, R. A universal role for MyD88 in TLR/IL-1R-mediated signaling. *Trends in biochemical sciences* **27**, 474-482 (2002).
78 St. Croix, B. *et al.* E-cadherin–dependent growth suppression is mediated by the cyclin-dependent kinase inhibitor p27KIP1. *The Journal of Cell Biology* **142**, 557-571 (1998).
79 Dan, H. C. *et al.* Akt phosphorylation and stabilization of X-linked inhibitor of apoptosis protein (XIAP). *Journal of Biological Chemistry* **291**, 22846 (2016). https://doi.org/10.1074/jbc.A116.312044
80 Karin, M. & Lin, A. NF-kappaB at the crossroads of life and death. *Nature Immunology* **3**, 221-227 (2002). https://doi.org/10.1038/ni0302-221
81 Del Bufalo, D., Trisciuoglio, D. & Milella, M. *Crosstalk between VEGF and bcl-2 in tumor progression and angiogenesis*, <https://www.ncbi.nlm.nih.gov/books/NBK6393/> (2013).
82 Catz, S. D. & Johnson, J. L. Transcriptional regulation of bcl-2 by nuclear factor kappa B and its significance in prostate cancer. *Oncogene* **20**, 7342-7351 (2001). https://doi.org/10.1038/sj.onc.1204926
83 Breitschopf, K., Haendeler, J., Malchow, P., Zeiher, A. M. & Dimmeler, S. Posttranslational modification of Bcl-2 facilitates its proteasome-dependent degradation: molecular characterization of the involved signaling pathway. *Molecular and Cellular Biology* **20**, 1886-1896 (2000).
84 Jie, Z. *Cell and Molecular Biology of Tumors (in Chinese)*. (Science Press 2017).
85 Vogelstein, B., Lane, D. & Levine, A. J. Surfing the p53 network. *Nature* **408**, 307-310 (2000).
86 Levine, A. J. p53, the cellular gatekeeper for growth and division. *Cell* **88**, 323-331 (1997).
87 Rahimi, R. A. & Leof, E. B. TGF-beta signaling: a tale of two responses. *Journal of Cellular Biochemistry* **102**, 593-608 (2007). https://doi.org/10.1002/jcb.21501
88 Hoffman, B. & Liebermann, D. A. Apoptotic signaling by c-MYC. *Oncogene* **27**, 6462-6472 (2008). https://doi.org/10.1038/onc.2008.312
89 Rodeck, U. *et al.* Regulation of Bcl-xL expression in human keratinocytes by cell-substratum adhesion and the epidermal growth factor receptor. *Proceedings of the National Academy of Sciences of the United States of America* **94**, 5067-5072 (1997). https://doi.org/10.1073/pnas.94.10.5067
90 Stoll, S. W. *et al.* EGF receptor signaling inhibits keratinocyte apoptosis: evidence for mediation by Bcl-XL. *Oncogene* **16**, 1493-1499 (1998). https://doi.org/10.1038/sj.onc.1201657
91 Jost, M., Huggett, T. M., Kari, C., Boise, L. H. & Rodeck, U. Epidermal growth factor receptor-

dependent control of keratinocyte survival and Bcl-xL expression through a MEK-dependent pathway. *Journal of Biological Chemistry* **276**, 6320-6326 (2001). https://doi.org/10.1074/jbc.M008210200
92 Jiang, P., Du, W. & Wu, M. P53 and bad: remote strangers become close friends. *Cell Research* **17**, 283-285 (2007). https://doi.org/10.1038/cr.2007.19
93 Datta, S. R. *et al.* Akt phosphorylation of BAD couples survival signals to the cell-intrinsic death machinery. *Cell* **91**, 231-241 (1997). https://doi.org/10.1016/s0092-8674(00)80405-5
94 She, Q. B. *et al.* The BAD protein integrates survival signaling by EGFR/MAPK and PI3K/Akt kinase pathways in PTEN-deficient tumor cells. *Cancer Cell* **8**, 287-297 (2005). https://doi.org/10.1016/j.ccr.2005.09.006
95 Zhang, Z. *et al.* Bcl-2 homodimerization involves two distinct binding surfaces, a topographic arrangement that provides an effective mechanism for Bcl-2 to capture activated Bax. *Journal of Biological Chemistry* **279**, 43920-43928 (2004). https://doi.org/10.1074/jbc.M406412200
96 Chipuk, J. E., Bhat, M., Hsing, A. Y., Ma, J. & Danielpour, D. Bcl-xL blocks transforming growth factor-beta 1-induced apoptosis by inhibiting cytochrome c release and not by directly antagonizing Apaf-1-dependent caspase activation in prostate epithelial cells. *Journal of Biological Chemistry* **276**, 26614-26621 (2001). https://doi.org/10.1074/jbc.M100913200
97 Xu, X., Fu, X. Y., Plate, J. & Chong, A. S. IFN-gamma induces cell growth inhibition by Fas-mediated apoptosis: requirement of STAT1 protein for up-regulation of Fas and FasL expression. *Cancer Research* **58**, 2832-2837 (1998).
98 Peli, J. *et al.* Oncogenic Ras inhibits Fas ligand-mediated apoptosis by downregulating the expression of Fas. *Embo Journal* **18**, 1824-1831 (1999). https://doi.org/10.1093/emboj/18.7.1824
99 Rozakis-Adcock, M., Fernley, R., Wade, J., Pawson, T. & Bowtell, D. The SH2 and SH3 domains of mammalian Grb2 couple the EGF receptor to the Ras activator mSos1. *Nature* **363**, 83-85 (1993). https://doi.org/10.1038/363083a0
100 Ferrara, N., Gerber, H. P. & LeCouter, J. The biology of VEGF and its receptors. *Nature Medicine* **9**, 669-676 (2003). https://doi.org/10.1038/nm0603-669
101 Eliceiri, B. P. Integrin and growth factor receptor crosstalk. *Circulation Research* **89**, 1104-1110 (2001). https://doi.org/10.1161/hh2401.101084
102 Stupack, D. G. & Cheresh, D. A. Get a ligand, get a life: integrins, signaling and cell survival. *Journal of Cell Science* **115**, 3729-3738 (2002). https://doi.org/10.1242/jcs.00071
103 Eastman-Reks, S. B. & Vedeckis, W. V. Glucocorticoid inhibition of c-myc, c-myb, and c-Ki-ras expression in a mouse lymphoma cell line. *Cancer Research* **46**, 2457-2462 (1986).
104 Hocevar, B. A., Brown, T. L. & Howe, P. H. TGF-beta induces fibronectin synthesis through a c-Jun N-terminal kinase-dependent, Smad4-independent pathway. *Embo Journal* **18**, 1345-1356 (1999). https://doi.org/10.1093/emboj/18.5.1345
105 Engel, M. E., McDonnell, M. A., Law, B. K. & Moses, H. L. Interdependent SMAD and JNK signaling in transforming growth factor-beta-mediated transcription. *Journal of Biological Chemistry* **274**, 37413-37420 (1999). https://doi.org/10.1074/jbc.274.52.37413
106 Kant, S. *et al.* TNF-stimulated MAP kinase activation mediated by a Rho family GTPase signaling pathway. *Genes & Development* **25**, 2069-2078 (2011).

https://doi.org/10.1101/gad.17224711
107 Finch, A., Holland, P., Cooper, J., Saklatvala, J. & Kracht, M. Selective activation of JNK/SAPK by interleukin-1 in rabbit liver is mediated by MKK7. *Febs Letters* **418**, 144-148 (1997). https://doi.org/10.1016/s0014-5793(97)01364-1
108 Holtmann, H. *et al.* The MAPK kinase kinase TAK1 plays a central role in coupling the interleukin-1 receptor to both transcriptional and RNA-targeted mechanisms of gene regulation. *Journal of Biological Chemistry* **276**, 3508-3516 (2001). https://doi.org/10.1074/jbc.M004376200
109 Hammaker, D. R., Boyle, D. L., Chabaud-Riou, M. & Firestein, G. S. Regulation of c-Jun N-terminal kinase by MEKK-2 and mitogen-activated protein kinase kinase kinases in rheumatoid arthritis. *Journal of Immunology* **172**, 1612-1618 (2004). https://doi.org/10.4049/jimmunol.172.3.1612
110 Citri, A. & Yarden, Y. EGF-ERBB signalling: towards the systems level. *Nature Reviews Molecular Cell Biology* **7**, 505-516 (2006). https://doi.org/10.1038/nrm1962
111 Waite, K. A. & Eng, C. Protean PTEN: form and function. *American Journal of Human Genetics* **70**, 829-844 (2002). https://doi.org/10.1086/340026
112 Giancotti, F. G. & Ruoslahti, E. Integrin signaling. *Science* **285**, 1028-1032 (1999). https://doi.org/10.1126/science.285.5430.1028
113 Pylayeva-Gupta, Y., Grabocka, E. & Bar-Sagi, D. RAS oncogenes: weaving a tumorigenic web. *Nature Reviews Cancer* **11**, 761-774 (2011). https://doi.org/10.1038/nrc3106
114 Saraiva, M. & O'Garra, A. The regulation of IL-10 production by immune cells. *Nature Reviews Immunology* **10**, 170-181 (2010). https://doi.org/10.1038/nri2711
115 Raghoebir, L. *et al.* SOX2 redirects the developmental fate of the intestinal epithelium toward a premature gastric phenotype. *Journal of Molecular Cell Biology* **4**, 377-385 (2012). https://doi.org/10.1093/jmcb/mjs030
116 Chakladar, A. *et al.* Synergistic activation of the murine gastrin promoter by oncogenic Ras and beta-catenin involves SMAD recruitment. *Biochemical and Biophysical Research Communications* **336**, 190-196 (2005). https://doi.org/10.1016/j.bbrc.2005.08.061
117 Lei, S., Dubeykovskiy, A., Chakladar, A., Wojtukiewicz, L. & Wang, T. C. The murine gastrin promoter is synergistically activated by transforming growth factor-beta/Smad and Wnt signaling pathways. *Journal of Biological Chemistry* **279**, 42492-42502 (2004). https://doi.org/10.1074/jbc.M404025200
118 Mariotti, A. *et al.* EGF-R signaling through Fyn kinase disrupts the function of integrin alpha6beta4 at hemidesmosomes: role in epithelial cell migration and carcinoma invasion. *Journal of Cell Biology* **155**, 447-458 (2001). https://doi.org/10.1083/jcb.200105017
119 Chan, P. C., Chen, S. Y., Chen, C. H. & Chen, H. C. Crosstalk between hepatocyte growth factor and integrin signaling pathways. *Journal of Biomedical Science* **13**, 215-223 (2006). https://doi.org/10.1007/s11373-005-9061-7
120 Desgrosellier, J. S. & Cheresh, D. A. Integrins in cancer: biological implications and therapeutic opportunities. *Nature Reviews Cancer* **10**, 9-22 (2010). https://doi.org/10.1038/nrc2748
121 Avizienyte, E. *et al.* Src-induced de-regulation of E-cadherin in colon cancer cells requires

integrin signalling. *Molecular Pharmacology* **4**, 632-638 (2002). https://doi.org/10.1038/ncb829
122 Chan, A. O. *et al.* Stability of E-cadherin methylation status in gastric mucosa associated with histology changes. *Alimentary Pharmacology & Therapeutics* **24**, 831-836 (2006). https://doi.org/10.1111/j.1365-2036.2006.03032.x
123 Steinhusen, U. *et al.* Cleavage and shedding of E-cadherin after induction of apoptosis. *Journal of Biological Chemistry* **276**, 4972-4980 (2001). https://doi.org/10.1074/jbc.M006102200
124 Conacci-Sorrell, M. *et al.* Autoregulation of E-cadherin expression by cadherin-cadherin interactions: the roles of beta-catenin signaling, Slug, and MAPK. *Journal of Cell Biology* **163**, 847-857 (2003). https://doi.org/10.1083/jcb.200308162
125 Nelson, W. J. & Nusse, R. Convergence of Wnt, beta-catenin, and cadherin pathways. *Science* **303**, 1483-1487 (2004). https://doi.org/10.1126/science.1094291
126 Mansukhani, A., Ambrosetti, D., Holmes, G., Cornivelli, L. & Basilico, C. Sox2 induction by FGF and FGFR2 activating mutations inhibits Wnt signaling and osteoblast differentiation. *Journal of Cell Biology* **168**, 1065-1076 (2005). https://doi.org/10.1083/jcb.200409182
127 Vivanco, I. & Sawyers, C. L. The phosphatidylinositol 3-kinase-AKT pathway in human cancer. *Nature Reviews Cancer* **2**, 489-501 (2002).
128 Gunaratnam, L. *et al.* Hypoxia inducible factor activates the transforming growth factor-alpha/epidermal growth factor receptor growth stimulatory pathway in VHL(-/-) renal cell carcinoma cells. *Journal of Biological Chemistry* **278**, 44966-44974 (2003). https://doi.org/10.1074/jbc.M305502200
129 Skinner, H. D., Zheng, J. Z., Fang, J., Agani, F. & Jiang, B. H. Vascular endothelial growth factor transcriptional activation is mediated by hypoxia-inducible factor 1alpha, HDM2, and p70S6K1 in response to phosphatidylinositol 3-kinase/AKT signaling. *Journal of Biological Chemistry* **279**, 45643-45651 (2004). https://doi.org/10.1074/jbc.M404097200
130 Iñiguez, M. A., Rodríguez, A., Volpert, O. V., Fresno, M. & Redondo, J. M. Cyclooxygenase-2: a therapeutic target in angiogenesis. *Trends in Molecular Medicine* **9**, 73-78 (2003). https://doi.org/10.1016/s1471-4914(02)00011-4
131 Hatakeyama, M. *et al.* Interleukin-1 induces the expression of vascular endothelial growth factor in human pericardial mesothelial cells. *Heart Vessels* **22**, 123-127 (2007). https://doi.org/10.1007/s00380-006-0942-0
132 Niu, G. *et al.* Constitutive Stat3 activity up-regulates VEGF expression and tumor angiogenesis. *Oncogene* **21**, 2000-2008 (2002). https://doi.org/10.1038/sj.onc.1205260
133 Chang, Y. J. *et al.* Induction of cyclooxygenase-2 overexpression in human gastric epithelial cells by Helicobacter pylori involves TLR2/TLR9 and c-Src-dependent nuclear factor-kappaB activation. *Molecular Pharmacology* **66**, 1465-1477 (2004). https://doi.org/10.1124/mol.104.005199
134 Nakao, S. *et al.* Activation of NFkappaB is necessary for IL-1beta-induced cyclooxygenase-2 (COX-2) expression in human gingival fibroblasts. *Molecular and Cellular Biochemistry* **209**, 113-118 (2000). https://doi.org/10.1023/a:1007155525020
135 Chang, Y. J., Wu, M. S., Lin, J. T. & Chen, C. C. Helicobacter pylori-Induced invasion and angiogenesis of gastric cells is mediated by cyclooxygenase-2 induction through TLR2/TLR9

and promoter regulation. *Journal of Immunology* **175**, 8242-8252 (2005). https://doi.org/10.4049/jimmunol.175.12.8242

136 Singer, C. A. *et al.* p38 MAPK and NF-kappaB mediate COX-2 expression in human airway myocytes. *American Journal of Physiology-Lung Cellular and Molecular Physiology* **285**, L1087-L1098 (2003). https://doi.org/10.1152/ajplung.00409.2002

137 Peluso, J. J., Pappalardo, A. & Fernandez, G. E-cadherin-mediated cell contact prevents apoptosis of spontaneously immortalized granulosa cells by regulating Akt kinase activity. *Biology Reprod* **64**, 1183-1190 (2001). https://doi.org/10.1095/biolreprod64.4.1183

138 Guo, W. & Giancotti, F. G. Integrin signalling during tumour progression. *Nature Reviews Molecular Cell Biology* **5**, 816-826 (2004). https://doi.org/10.1038/nrm1490

139 Achyut, B. R. & Yang, L. Transforming growth factor-β in the gastrointestinal and hepatic tumor microenvironment. *Gastroenterology* **141**, 1167-1178 (2011). https://doi.org/10.1053/j.gastro.2011.07.048

140 Hara, S. *et al.* Akt activation in renal cell carcinoma: contribution of a decreased PTEN expression and the induction of apoptosis by an Akt inhibitor. *Annals of Oncology* **16**, 928-933 (2005). https://doi.org/10.1093/annonc/mdi182

141 Vasudevan, K. M., Gurumurthy, S. & Rangnekar, V. M. Suppression of PTEN expression by NF-kappa B prevents apoptosis. *Molecular and Cellular Biology* **24**, 1007-1021 (2004). https://doi.org/10.1128/mcb.24.3.1007-1021.2004

142 Lau, M. T., Klausen, C. & Leung, P. C. E-cadherin inhibits tumor cell growth by suppressing PI3K/Akt signaling via β-catenin-Egr1-mediated PTEN expression. *Oncogene* **30**, 2753-2766 (2011). https://doi.org/10.1038/onc.2011.6

143 Laughner, E., Taghavi, P., Chiles, K., Mahon, P. C. & Semenza, G. L. HER2 (neu) signaling increases the rate of hypoxia-inducible factor 1alpha (HIF-1alpha) synthesis: novel mechanism for HIF-1-mediated vascular endothelial growth factor expression. *Molecular and Cellular Biology* **21**, 3995-4004 (2001). https://doi.org/10.1128/mcb.21.12.3995-4004.2001

144 Belaiba, R. S. *et al.* Hypoxia up-regulates hypoxia-inducible factor-1alpha transcription by involving phosphatidylinositol 3-kinase and nuclear factor kappaB in pulmonary artery smooth muscle cells. *Molecular Biology of The Cell* **18**, 4691-4697 (2007). https://doi.org/10.1091/mbc.e07-04-0391

145 Görlach, A. & Bonello, S. The cross-talk between NF-kappaB and HIF-1: further evidence for a significant liaison. *Biochemical Journal* **412**, e17-19 (2008). https://doi.org/10.1042/bj20080920

146 Site, L. *Endogenous molecular network reveals mechanisms of heterogeneity within gastric cancer* doctor thesis, Shanghai Jiao Tong University, (2016).

147 Ravi, R. *et al.* Regulation of tumor angiogenesis by p53-induced degradation of hypoxia-inducible factor 1alpha. *Genes & Development* **14**, 34-44 (2000).

148 Cohen, P. & Frame, S. The renaissance of GSK3. *Nature Reviews Molecular Cell Biology* **2**, 769-776 (2001). https://doi.org/10.1038/35096075

149 Meng, F., Liu, L., Chin, P. C. & D'Mello, S. R. Akt is a downstream target of NF-kappa B. *Journal of Biological Chemistry* **277**, 29674-29680 (2002). https://doi.org/10.1074/jbc.M112464200

150 Finco, T. S. *et al.* Oncogenic Ha-Ras-induced signaling activates NF-kappaB transcriptional activity, which is required for cellular transformation. *Journal of Biological Chemistry* **272**, 24113-24116 (1997). https://doi.org/10.1074/jbc.272.39.24113

151 Zandi, E. & Karin, M. Bridging the gap: Composition, regulation, and physiological function of the IkappaB kinase complex. *Molecular and Cellular Biology* **19**, 4547-4551 (1999). https://doi.org/10.1128/mcb.19.7.4547

152 Driessler, F., Venstrom, K., Sabat, R., Asadullah, K. & Schottelius, A. J. Molecular mechanisms of interleukin-10-mediated inhibition of NF-kappaB activity: a role for p50. *Clinical and Experimental Immunology* **135**, 64-73 (2004). https://doi.org/10.1111/j.1365-2249.2004.02342.x

153 Murray, P. J. Understanding and exploiting the endogenous interleukin-10/STAT3-mediated anti-inflammatory response. *Current Opinion in Pharmacology* **6**, 379-386 (2006). https://doi.org/10.1016/j.coph.2006.01.010

154 Sun, S. C., Ganchi, P. A., Ballard, D. W. & Greene, W. C. NF-kappa B controls expression of inhibitor I kappa B alpha: evidence for an inducible autoregulatory pathway. *Science* **259**, 1912-1915 (1993). https://doi.org/10.1126/science.8096091

155 Griffin, B. D. & Moynagh, P. N. In vivo binding of NF-kappaB to the IkappaBbeta promoter is insufficient for transcriptional activation. *Biochemical Journal* **400**, 115-125 (2006). https://doi.org/10.1042/bj20060786

156 Weber, A., Wasiliew, P. & Kracht, M. Interleukin-1 (IL-1) pathway. *Science Signaling* **3**, cm1 (2010). https://doi.org/10.1126/scisignal.3105cm1

157 Windheim, M., Stafford, M., Peggie, M. & Cohen, P. Interleukin-1 (IL-1) induces the Lys63-linked polyubiquitination of IL-1 receptor-associated kinase 1 to facilitate NEMO binding and the activation of IkappaBalpha kinase. *Molecular and Cellular Biology* **28**, 1783-1791 (2008). https://doi.org/10.1128/mcb.02380-06

158 Romashkova, J. A. & Makarov, S. S. NF-kappaB is a target of AKT in anti-apoptotic PDGF signalling. *Nature* **401**, 86-90 (1999). https://doi.org/10.1038/43474

159 Madrid, L. V. *et al.* Akt suppresses apoptosis by stimulating the transactivation potential of the RelA/p65 subunit of NF-kappaB. *Molecular and Cellular Biology* **20**, 1626-1638 (2000). https://doi.org/10.1128/mcb.20.5.1626-1638.2000

160 Burow, M. E. *et al.* PI3-K/AKT regulation of NF-kappaB signaling events in suppression of TNF-induced apoptosis. *Biochemical and Biophysical Research Communications* **271**, 342-345 (2000). https://doi.org/10.1006/bbrc.2000.2626

161 Schneider, P. *et al.* TRAIL receptors 1 (DR4) and 2 (DR5) signal FADD-dependent apoptosis and activate NF-kappaB. *Immunity* **7**, 83183-83186 (1997). https://doi.org/10.1016/s1074-7613(00)80401-x

162 Derynck, R. & Zhang, Y. E. Smad-dependent and Smad-independent pathways in TGF-beta family signalling. *Nature* **425**, 577-584 (2003). https://doi.org/10.1038/nature02006

163 Rutault, K., Hazzalin, C. A. & Mahadevan, L. C. Combinations of ERK and p38 MAPK inhibitors ablate tumor necrosis factor-alpha (TNF-alpha) mRNA induction. Evidence for selective destabilization of TNF-alpha transcripts. *Journal of Biological Chemistry* **276**, 6666-6674 (2001). https://doi.org/10.1074/jbc.M005486200

164 Rajasingh, J. *et al.* IL-10-induced TNF-alpha mRNA destabilization is mediated via IL-10 suppression of p38 MAP kinase activation and inhibition of HuR expression. *The Faseb Journal* **20**, 2112-2114 (2006). https://doi.org/10.1096/fj.06-6084fje
165 Foey, A. D. *et al.* Regulation of monocyte IL-10 synthesis by endogenous IL-1 and TNF-alpha: role of the p38 and p42/44 mitogen-activated protein kinases. *Journal of Immunology* **160**, 920-928 (1998).
166 Walker, P. R., Saas, P. & Dietrich, P. Y. Role of Fas ligand (CD95L) in immune escape: the tumor cell strikes back. *Journal of Immunology* **158**, 4521-4524 (1997).
167 Ferguson, T. A. & Griffith, T. S. A vision of cell death: Fas ligand and immune privilege 10 years later. *Immunological Reviews* **213**, 228-238 (2006). https://doi.org/10.1111/j.1600-065X.2006.00430.x
168 Hu, X. *et al.* IFN-gamma suppresses IL-10 production and synergizes with TLR2 by regulating GSK3 and CREB/AP-1 proteins. *Immunity* **24**, 563-574 (2006). https://doi.org/10.1016/j.immuni.2006.02.014
169 Paul, W. E. *Fundamental immunology*. (Lippincott Williams & Wilkins, 2012).
170 Dinarello, C. A. *et al.* Interleukin 1 induces interleukin 1. I. Induction of circulating interleukin 1 in rabbits in vivo and in human mononuclear cells in vitro. *Journal of Immunology* **139**, 1902-1910 (1987).
171 Brissoni, B. *et al.* Intracellular trafficking of interleukin-1 receptor I requires Tollip. *Current Biology* **16**, 2265-2270 (2006). https://doi.org/10.1016/j.cub.2006.09.062
172 Burns, K. *et al.* Tollip, a new component of the IL-1RI pathway, links IRAK to the IL-1 receptor. *Nature Cell Biology* **2**, 346-351 (2000). https://doi.org/10.1038/35014038
173 Didierlaurent, A. *et al.* Tollip regulates proinflammatory responses to interleukin-1 and lipopolysaccharide. *Molecular and Cellular Biology* **26**, 735-742 (2006). https://doi.org/10.1128/mcb.26.3.735-742.2006
174 Akira, S. The role of IL-18 in innate immunity. *Current Opinion in Immunology* **12**, 59-63 (2000). https://doi.org/10.1016/s0952-7915(99)00051-5
175 Gołab, J. *et al.* Direct stimulation of macrophages by IL-12 and IL-18--a bridge too far? *Immunology Letters* **72**, 153-157 (2000). https://doi.org/10.1016/s0165-2478(00)00178-4
176 Otani, T. *et al.* Identification of IFN-gamma-producing cells in IL-12/IL-18-treated mice. *Cell Immunol* **198**, 111-119 (1999). https://doi.org/10.1006/cimm.1999.1589
177 Cope, A., Le Friec, G., Cardone, J. & Kemper, C. The Th1 life cycle: molecular control of IFN-γ to IL-10 switching. *Trends Immunol* **32**, 278-286 (2011). https://doi.org/10.1016/j.it.2011.03.010
178 Munger, J. S. *et al.* The integrin alpha v beta 6 binds and activates latent TGF beta 1: a mechanism for regulating pulmonary inflammation and fibrosis. *Cell* **96**, 319-328 (1999). https://doi.org/10.1016/s0092-8674(00)80545-0
179 Mu, D. *et al.* The integrin alpha(v)beta8 mediates epithelial homeostasis through MT1-MMP-dependent activation of TGF-beta1. *Journal of Cell Biology* **157**, 493-507 (2002). https://doi.org/10.1083/jcb.200109100
180 Gaber, T., Dziurla, R., Tripmacher, R., Burmester, G. R. & Buttgereit, F. Hypoxia inducible factor (HIF) in rheumatology: low O2! See what HIF can do! *Annals of the Rheumatic Diseases*

64, 971-980 (2005). https://doi.org/10.1136/ard.2004.031641
181 Shah, C. A., Bei, L., Wang, H., Platanias, L. C. & Eklund, E. A. HoxA10 protein regulates transcription of gene encoding fibroblast growth factor 2 (FGF2) in myeloid cells. *Journal of Biological Chemistry* **287**, 18230-18248 (2012). https://doi.org/10.1074/jbc.M111.328401
182 Annes, J. P., Munger, J. S. & Rifkin, D. B. Making sense of latent TGFbeta activation. *Journal of Cell Science* **116**, 217-224 (2003). https://doi.org/10.1242/jcs.00229
183 Massagué, J., Seoane, J. & Wotton, D. Smad transcription factors. *Genes & Development* **19**, 2783-2810 (2005). https://doi.org/10.1101/gad.1350705
184 Bitzer, M. *et al.* A mechanism of suppression of TGF-beta/SMAD signaling by NF-kappa B/RelA. *Genes & Development* **14**, 187-197 (2000).
185 Froese, N. *et al.* GATA6 promotes angiogenic function and survival in endothelial cells by suppression of autocrine transforming growth factor beta/activin receptor-like kinase 5 signaling. *Journal of Biological Chemistry* **286**, 5680-5690 (2011). https://doi.org/10.1074/jbc.M110.176925
186 Tompkins, D. H. *et al.* Sox2 is required for maintenance and differentiation of bronchiolar clara, ciliated, and goblet cells. *PLoS One* **4**, e8248 (2009). https://doi.org/10.1371/journal.pone.0008248
187 Kisseleva, T., Bhattacharya, S., Braunstein, J. & Schindler, C. W. Signaling through the JAK/STAT pathway, recent advances and future challenges. *Gene* **285**, 1-24 (2002). https://doi.org/10.1016/s0378-1119(02)00398-0
188 Henson, E. S. & Gibson, S. B. Surviving cell death through epidermal growth factor (EGF) signal transduction pathways: implications for cancer therapy. *Cell Signal* **18**, 2089-2097 (2006). https://doi.org/10.1016/j.cellsig.2006.05.015
189 Ferrand, A. & Wang, T. C. Gastrin and cancer: a review. *Cancer Letters* **238**, 15-29 (2006). https://doi.org/10.1016/j.canlet.2005.06.025
190 Starsíchová, A., Lincová, E., Pernicová, Z., Kozubík, A. & Soucek, K. TGF-beta1 suppresses IL-6-induced STAT3 activation through regulation of Jak2 expression in prostate epithelial cells. *Cell Signal* **22**, 1734-1744 (2010). https://doi.org/10.1016/j.cellsig.2010.06.014
191 Walia, B., Wang, L., Merlin, D. & Sitaraman, S. V. TGF-beta down-regulates IL-6 signaling in intestinal epithelial cells: critical role of SMAD-2. *The Faseb Journal* **17**, 2130-2132 (2003). https://doi.org/10.1096/fj.02-1211fje
192 Bailly, A., Späth, G., Bender, V. & Weiss, M. C. Phenotypic effects of the forced expression of HNF4 and HNF1alpha are conditioned by properties of the recipient cell. *Journal of Cell Science* **111**, 2411-2421 (1998). https://doi.org/10.1242/jcs.111.16.2411
193 Guo, H., Ma, O., Speck, N. A. & Friedman, A. D. Runx1 deletion or dominant inhibition reduces Cebpa transcription via conserved promoter and distal enhancer sites to favor monopoiesis over granulopoiesis. *Blood* **119**, 4408-4418 (2012). https://doi.org/10.1182/blood-2011-12-397091
194 Anderson, E. R. *et al.* The hypoxia-inducible factor-C/EBPα axis controls ethanol-mediated hepcidin repression. *Molecular and Cellular Biology* **32**, 4068-4077 (2012). https://doi.org/10.1128/mcb.00723-12
195 An, M. R. *et al.* Evidence for posttranscriptional regulation of C/EBPalpha and C/EBPbeta

isoform expression during the lipopolysaccharide-mediated acute-phase response. *Molecular and Cellular Biology* **16**, 2295-2306 (1996). https://doi.org/10.1128/mcb.16.5.2295
196 Spinella-Jaegle, S. *et al.* Sonic hedgehog increases the commitment of pluripotent mesenchymal cells into the osteoblastic lineage and abolishes adipocytic differentiation. *Journal of Cell Science* **114**, 2085-2094 (2001). https://doi.org/10.1242/jcs.114.11.2085
197 Coskun, M., Troelsen, J. T. & Nielsen, O. H. The role of CDX2 in intestinal homeostasis and inflammation. *Biochim Biophys Acta* **1812**, 283-289 (2011). https://doi.org/10.1016/j.bbadis.2010.11.008
198 D'Angelo, A. *et al.* Hepatocyte nuclear factor 1alpha and beta control terminal differentiation and cell fate commitment in the gut epithelium. *Development* **137**, 1573-1582 (2010). https://doi.org/10.1242/dev.044420
199 Saandi, T. *et al.* Regulation of the tumor suppressor homeogene Cdx2 by HNF4α in intestinal cancer. *Oncogene* **32**, 3782-3788 (2013). https://doi.org/10.1038/onc.2012.401
200 Zhao, T. *et al.* β-catenin regulates Pax3 and Cdx2 for caudal neural tube closure and elongation. *Development* **141**, 148-157 (2014). https://doi.org/10.1242/dev.101550
201 Adachi, K., Suemori, H., Yasuda, S. Y., Nakatsuji, N. & Kawase, E. Role of SOX2 in maintaining pluripotency of human embryonic stem cells. *Genes Cells* **15**, 455-470 (2010). https://doi.org/10.1111/j.1365-2443.2010.01400.x
202 Ura, H. *et al.* STAT3 and Oct-3/4 control histone modification through induction of Eed in embryonic stem cells. *Journal of Biological Chemistry* **283**, 9713-9723 (2008). https://doi.org/10.1074/jbc.M707275200
203 Selesniemi, K., Reedy, M., Gultice, A., Guilbert, L. J. & Brown, T. L. Transforming growth factor-beta induces differentiation of the labyrinthine trophoblast stem cell line SM10. *Stem Cells and Development* **14**, 697-711 (2005). https://doi.org/10.1089/scd.2005.14.697
204 Mavromatakis, Y. E. *et al.* Foxa1 and Foxa2 positively and negatively regulate Shh signalling to specify ventral midbrain progenitor identity. *Mechanisms of Development* **128**, 90-103 (2011). https://doi.org/10.1016/j.mod.2010.11.002
205 Martis, P. C. *et al.* C/EBPalpha is required for lung maturation at birth. *Development* **133**, 1155-1164 (2006). https://doi.org/10.1242/dev.02273
206 Bayly, R. D., Brown, C. Y. & Agarwala, S. A novel role for FOXA2 and SHH in organizing midbrain signaling centers. *Developmental Biology* **369**, 32-42 (2012). https://doi.org/10.1016/j.ydbio.2012.06.018
207 Howell, J. J. & Stoffel, M. Nuclear export-independent inhibition of Foxa2 by insulin. *Journal of Biological Chemistry* **284**, 24816-24824 (2009). https://doi.org/10.1074/jbc.M109.042135
208 Tian, Y. *et al.* Characterization and in vivo pharmacological rescue of a Wnt2-Gata6 pathway required for cardiac inflow tract development. *Developmental Cell* **18**, 275-287 (2010). https://doi.org/10.1016/j.devcel.2010.01.008
209 Murakami, A., Shen, H., Ishida, S. & Dickson, C. SOX7 and GATA-4 are competitive activators of Fgf-3 transcription. *Journal of Biological Chemistry* **279**, 28564-28573 (2004). https://doi.org/10.1074/jbc.M313814200
210 Shirai, T. *et al.* Identification of an enhancer that controls up-regulation of fibronectin during differentiation of embryonic stem cells into extraembryonic endoderm. *Journal of Biological*

Chemistry **280**, 7244-7252 (2005). https://doi.org/10.1074/jbc.M410731200
211 Odom, D. T. *et al.* Control of pancreas and liver gene expression by HNF transcription factors. *Science* **303**, 1378-1381 (2004). https://doi.org/10.1126/science.1089769
212 Afelik, S. *et al.* Notch-mediated patterning and cell fate allocation of pancreatic progenitor cells. *Development* **139**, 1744-1753 (2012). https://doi.org/10.1242/dev.075804
213 Yamasaki, H. *et al.* Suppression of C/EBPalpha expression in periportal hepatoblasts may stimulate biliary cell differentiation through increased Hnf6 and Hnf1b expression. *Development* **133**, 4233-4243 (2006). https://doi.org/10.1242/dev.02591
214 Hatzis, P., Kyrmizi, I. & Talianidis, I. Mitogen-activated protein kinase-mediated disruption of enhancer-promoter communication inhibits hepatocyte nuclear factor 4alpha expression. *Molecular and Cellular Biology* **26**, 7017-7029 (2006). https://doi.org/10.1128/mcb.00297-06
215 Peignon, G. *et al.* E-cadherin-dependent transcriptional control of apolipoprotein A-IV gene expression in intestinal epithelial cells: a role for the hepatic nuclear factor 4. *Journal of Biological Chemistry* **281**, 3560-3568 (2006). https://doi.org/10.1074/jbc.M506360200
216 Morrisey, E. E. *et al.* GATA6 regulates HNF4 and is required for differentiation of visceral endoderm in the mouse embryo. *Genes & Development* **12**, 3579-3590 (1998). https://doi.org/10.1101/gad.12.22.3579
217 Ning, B. F. *et al.* Hepatocyte nuclear factor 4α-nuclear factor-κB feedback circuit modulates liver cancer progression. *Hepatology* **60**, 1607-1619 (2014). https://doi.org/10.1002/hep.27177
218 Maeda, Y. *et al.* Tumour suppressor p53 down-regulates the expression of the human hepatocyte nuclear factor 4alpha (HNF4alpha) gene. *Biochemical Journal* **400**, 303-313 (2006). https://doi.org/10.1042/bj20060614
219 Hatziapostolou, M. *et al.* An HNF4α-miRNA inflammatory feedback circuit regulates hepatocellular oncogenesis. *Cell* **147**, 1233-1247 (2011). https://doi.org/10.1016/j.cell.2011.10.043
220 In der Rieden, P. M., Vilaspasa, F. L. & Durston, A. J. Xwnt8 directly initiates expression of labial Hox genes. *Developmental Dynamics* **239**, 126-139 (2010). https://doi.org/10.1002/dvdy.22020
221 Kidder, B. L., Yang, J. & Palmer, S. Stat3 and c-Myc genome-wide promoter occupancy in embryonic stem cells. *PLoS One* **3**, e3932 (2008). https://doi.org/10.1371/journal.pone.0003932
222 Rawat, V. P. *et al.* Overexpression of CDX2 perturbs HOX gene expression in murine progenitors depending on its N-terminal domain and is closely correlated with deregulated HOX gene expression in human acute myeloid leukemia. *Blood* **111**, 309-319 (2008). https://doi.org/10.1182/blood-2007-04-085407
223 Magnusson, M. *et al.* HOXA10 is a critical regulator for hematopoietic stem cells and erythroid/megakaryocyte development. *Blood* **109**, 3687-3696 (2007). https://doi.org/10.1182/blood-2006-10-054676
224 Ross, K. *et al.* Polycomb group ring finger 1 cooperates with Runx1 in regulating differentiation and self-renewal of hematopoietic cells. *Blood* **119**, 4152-4161 (2012). https://doi.org/10.1182/blood-2011-09-382390
225 Scholl, C. *et al.* The homeobox gene CDX2 is aberrantly expressed in most cases of acute

myeloid leukemia and promotes leukemogenesis. *Journal of Clinical Investigation* **117**, 1037-1048 (2007). https://doi.org/10.1172/jci30182
226 Weiss, G., Goldsmith, L. T., Taylor, R. N., Bellet, D. & Taylor, H. S. Inflammation in reproductive disorders. *Reproductive Sciences* **16**, 216-229 (2009). https://doi.org/10.1177/1933719108330087
227 Rawat, V. P., Humphries, R. K. & Buske, C. Beyond Hox: the role of ParaHox genes in normal and malignant hematopoiesis. *Blood* **120**, 519-527 (2012). https://doi.org/10.1182/blood-2012-02-385898
228 Guo, Y., Maillard, I., Chakraborti, S., Rothenberg, E. V. & Speck, N. A. Core binding factors are necessary for natural killer cell development and cooperate with Notch signaling during T-cell specification. *Blood* **112**, 480-492 (2008). https://doi.org/10.1182/blood-2007-10-120261
229 Iacovino, M. *et al.* HoxA3 is an apical regulator of haemogenic endothelium. *Nature Cell Biology* **13**, 72-78 (2011). https://doi.org/10.1038/ncb2137
230 Foshay, K. M. & Gallicano, G. I. Regulation of Sox2 by STAT3 initiates commitment to the neural precursor cell fate. *Stem Cells and Development* **17**, 269-278 (2008). https://doi.org/10.1089/scd.2007.0098
231 Takanaga, H. *et al.* Gli2 is a novel regulator of sox2 expression in telencephalic neuroepithelial cells. *Stem Cells* **27**, 165-174 (2009). https://doi.org/10.1634/stemcells.2008-0580
232 Boyer, L. A. *et al.* Core transcriptional regulatory circuitry in human embryonic stem cells. *Cell* **122**, 947-956 (2005). https://doi.org/10.1016/j.cell.2005.08.020
233 Okubo, T., Pevny, L. H. & Hogan, B. L. Sox2 is required for development of taste bud sensory cells. *Genes & Development* **20**, 2654-2659 (2006). https://doi.org/10.1101/gad.1457106
234 Marqués-Torrejón, M. *et al.* Cyclin-dependent kinase inhibitor p21 controls adult neural stem cell expansion by regulating Sox2 gene expression. *Cell Stem Cell* **12**, 88-100 (2013). https://doi.org/10.1016/j.stem.2012.12.001
235 Camilo, V. *et al.* Helicobacter pylori and the BMP pathway regulate CDX2 and SOX2 expression in gastric cells. *Carcinogenesis* **33**, 1985-1992 (2012). https://doi.org/10.1093/carcin/bgs233
236 Espinosa, L. *et al.* The Notch/Hes1 pathway sustains NF-κB activation through CYLD repression in T cell leukemia. *Cancer Cell* **18**, 268-281 (2010). https://doi.org/10.1016/j.ccr.2010.08.006
237 Grainger, S. *et al.* Cdx regulates Dll1 in multiple lineages. *Developmental Biology* **361**, 1-11 (2012). https://doi.org/10.1016/j.ydbio.2011.09.034
238 Uesaka, T., Kageyama, N. & Watanabe, H. Identifying target genes regulated downstream of Cdx2 by microarray analysis. *Journal of Molecular Biology* **337**, 647-660 (2004). https://doi.org/10.1016/j.jmb.2004.01.061
239 Yoshimatsu, T. *et al.* Non-cell-autonomous action of STAT3 in maintenance of neural precursor cells in the mouse neocortex. *Development* **133**, 2553-2563 (2006). https://doi.org/10.1242/dev.02419
240 Shi, Y. C. *et al.* C/EBPα inhibits hepatocellular carcinoma by reducing Notch3/Hes1/p27 cascades. *Digestive and Liver Disease* **45**, 844-851 (2013). https://doi.org/10.1016/j.dld.2013.03.013

241 Ferreira, A. C. *et al.* E-cadherin impairment increases cell survival through Notch-dependent upregulation of Bcl-2. *Human Molecular Genetics* **21**, 334-343 (2012). https://doi.org/10.1093/hmg/ddr469
242 Boucherat, O., Chakir, J. & Jeannotte, L. The loss of Hoxa5 function promotes Notch-dependent goblet cell metaplasia in lung airways. *Biology Open* **1**, 677-691 (2012). https://doi.org/10.1242/bio.20121701
243 Krejci, P. *et al.* Receptor tyrosine kinases activate canonical WNT/β-catenin signaling via MAP kinase/LRP6 pathway and direct β-catenin phosphorylation. *PLoS One* **7**, e35826 (2012). https://doi.org/10.1371/journal.pone.0035826
244 Akhmetshina, A. *et al.* Activation of canonical Wnt signalling is required for TGF-β-mediated fibrosis. *Nature Communications* **3**, 735 (2012). https://doi.org/10.1038/ncomms1734
245 Zhang, X. *et al.* Wnt signaling regulates the stemness of lung cancer stem cells and its inhibitors exert anticancer effect on lung cancer SPC-A1 cells. *Medical Oncology* **32**, 95 (2015). https://doi.org/10.1007/s12032-014-0462-1
246 Favaro, R. *et al.* Hippocampal development and neural stem cell maintenance require Sox2-dependent regulation of Shh. *Nature Neuroscience* **12**, 1248-1256 (2009). https://doi.org/10.1038/nn.2397
247 Mutoh, H., Hayakawa, H., Sashikawa, M., Sakamoto, H. & Sugano, K. Direct repression of Sonic Hedgehog expression in the stomach by Cdx2 leads to intestinal transformation. *Biochemical Journal* **427**, 423-434 (2010). https://doi.org/10.1042/bj20091177

B. From network topology to dynamical systems

In accordance with the equations (1)-(5) outlined in the main text, equations 1-55 are employed to depict the temporal dynamics of the concentration/activation levels of the 55 factors within the network. Additionally, equations 56-110 are utilized to represent the random distribution parameters η for these 55 factors in the network. It is worth noting that the parameter n is assigned a value of 3, while the parameter a is set to 10.

$$\frac{dx(1)}{dt}=\frac{10\cdot\left(x(3)^3+x(31)^3\right)}{1+10\cdot\left(x(3)^3+x(31)^3\right)}\cdot\frac{1}{1+10\cdot\left(x(5)^3+x(40)^3\right)}-x(1)\cdot x(56);$$

$$\frac{dx(2)}{dt}=\frac{10\cdot\left(x(3)^3+x(4)^3\right)}{1+10\cdot\left(x(3)^3+x(4)^3\right)}\cdot\frac{1}{1+10\cdot\left(x(5)^3+x(6)^3+x(19)^3\right)}-x(2)\cdot x(57);$$

$$\frac{dx(3)}{dt}=\frac{10\cdot\left(x(4)^3+x(22)^3+x(23)^3+x(29)^3+x(30)^3+x(32)^3+x(33)^3+x(35)^3\right)}{1+10\cdot\left(x(4)^3+x(22)^3+x(23)^3+x(29)^3+x(30)^3+x(32)^3+x(33)^3+x(35)^3\right)}\cdot\frac{1}{1+10\cdot\left(x(5)^3+x(7)^3+x(40)^3+x(41)^3\right)}-x(3)\cdot x(58);$$

$$\frac{dx(4)}{dt}=\frac{10\cdot\left(x(3)^3+x(4)^3\right)}{1+10\cdot\left(x(3)^3+x(4)^3\right)}\cdot\frac{1}{1+10\cdot\left(x(5)^3+x(8)^3\right)}-x(4)\cdot x(59);$$

$$\frac{dx(5)}{dt}=\frac{10\cdot\left(x(4)^3+x(7)^3+x(32)^3+x(33)^3+x(40)^3+x(41)^3+x(45)^3+x(50)^3+x(54)^3\right)}{1+10\cdot\left(x(4)^3+x(7)^3+x(32)^3+x(33)^3+x(40)^3+x(41)^3+x(45)^3+x(50)^3+x(54)^3\right)}\cdot\frac{1}{1+10\cdot\left(x(3)^3+x(30)^3\right)}-x(5)\cdot x(60);$$

$$\frac{dx(6)}{dt}=\frac{10\cdot\left(x(19)^3+x(21)^3+x(40)^3+x(41)^3+x(55)^3\right)}{1+10\cdot\left(x(19)^3+x(21)^3+x(40)^3+x(41)^3+x(55)^3\right)}\cdot\frac{1}{1+10\cdot\left(x(2)^3+x(3)^3+x(30)^3+x(33)^3\right)}-x(6)\cdot x(61);$$

$$\frac{dx(7)}{dt}=\frac{10\cdot\left(x(3)^3+x(4)^3+x(10)^3+x(19)^3+x(35)^3\right)}{1+10\cdot\left(x(3)^3+x(4)^3+x(10)^3+x(19)^3+x(35)^3\right)}\cdot\frac{1}{1+10\cdot\left(x(24)^3+x(30)^3\right)}-x(7)\cdot x(62);$$

$$\frac{dx(8)}{dt}=\frac{1}{1+10\cdot\left(x(1)^3+x(2)^3+x(9)^3\right)}-x(8)\cdot x(63);$$

$$\frac{dx(9)}{dt}=\frac{10\cdot\left(x(10)^3+x(11)^3\right)}{1+10\cdot\left(x(10)^3+x(11)^3\right)}\cdot\frac{1}{1+10\cdot\left(x(5)^3+x(12)^3\right)}-x(9)\cdot x(64);$$

$$\frac{dx(10)}{dt}=\frac{10\cdot\left(x(3)^3+x(4)^3+x(15)^3+x(16)^3\right)}{1+10\cdot\left(x(3)^3+x(4)^3+x(15)^3+x(16)^3\right)}\cdot\frac{1}{1+10\cdot\left(x(12)^3+x(30)^3\right)}-x(10)\cdot x(65);$$

$$\frac{dx(11)}{dt}=\frac{10\cdot\left(x(18)^3+x(37)^3+x(39)^3\right)}{1+10\cdot\left(x(18)^3+x(37)^3+x(39)^3\right)}\cdot\frac{1}{1+10\cdot x(35)^3}-x(11)\cdot x(66);$$

$$\frac{dx(12)}{dt}=\frac{10\cdot\left(x(30)^3+x(35)^3\right)}{1+10\cdot\left(x(30)^3+x(35)^3\right)}\cdot\frac{1}{1+10\cdot x(10)^3}-x(12)\cdot x(67);$$

$$\frac{dx(13)}{dt}=\frac{10\cdot\left(x(23)^3+x(26)^3+x(27)^3+x(33)^3+x(35)^3\right)}{1+10\cdot\left(x(23)^3+x(26)^3+x(27)^3+x(33)^3+x(35)^3\right)}\cdot\frac{1}{1+10\cdot\left(x(3)^3+x(7)^3+x(15)^3+x(16)^3+x(40)^3\right)}-x(13)\cdot x(68);$$

$$\frac{dx(14)}{dt}=\frac{10\cdot\left(x(31)^3+x(35)^3\right)}{1+10\cdot\left(x(31)^3+x(35)^3\right)}\cdot\frac{1}{1+10\cdot\left(x(3)^3+x(16)^3\right)}-x(14)\cdot x(69);$$

$$\frac{dx(15)}{dt}=\frac{10\cdot x(11)^3}{1+10\cdot x(11)^3}\cdot\frac{1}{1+10\cdot x(13)^3}-x(15)\cdot x(70);$$

$$\frac{dx(16)}{dt}=\frac{10\cdot x(7)^3}{1+10\cdot x(7)^3}\cdot\frac{1}{1+10\cdot\left(x(30)^3+x(33)^3\right)}-x(16)\cdot x(71);$$

$$\frac{dx(17)}{dt}=\frac{10\cdot\left(x(3)^3+x(7)^3+x(15)^3\right)}{1+10\cdot\left(x(3)^3+x(7)^3+x(15)^3\right)}\cdot\frac{1}{1+10\cdot\left(x(13)^3+x(14)^3\right)}-x(17)\cdot x(72);$$

$$\frac{dx(18)}{dt}=\frac{10\cdot\left(x(3)^3+x(35)^3+x(41)^3\right)}{1+10\cdot\left(x(3)^3+x(35)^3+x(41)^3\right)}\cdot\frac{1}{1+10\cdot\left(x(28)^3\right)}-x(18)\cdot x(73);$$

$$\frac{dx(19)}{dt}=\frac{1}{1+10\cdot\left(x(22)^3+x(30)^3+x(35)^3\right)}-x(19)\cdot x(74);$$

$$\frac{dx(20)}{dt}=\frac{10\cdot x(31)^3}{1+10\cdot x(31)^3}-x(20)\cdot x(75);$$

$$\frac{dx(21)}{dt}=\frac{1}{1+10\cdot\left(x(9)^3+x(20)^3+x(39)^3\right)}-x(21)\cdot x(76);$$

$$\frac{dx(22)}{dt}=\frac{10\cdot x(31)^3}{1+10\cdot x(31)^3}\cdot\frac{1}{1+10\cdot\left(x(21)^3+x(43)^3+x(55)^3\right)}-x(22)\cdot x(77);$$

$$\frac{dx(23)}{dt}=\frac{10\cdot x(55)^3}{1+10\cdot x(55)^3}\cdot\frac{1}{1+10\cdot\left(x(43)^3+x(45)^3\right)}-x(23)\cdot x(78);$$

$$\frac{dx(24)}{dt}=\frac{10\cdot\left(x(35)^3+x(42)^3+x(45)^3\right)}{1+10\cdot\left(x(35)^3+x(42)^3+x(45)^3\right)}\cdot\frac{1}{1+10\cdot\left(x(21)^3+x(44)^3+x(52)^3\right)}-x(24)\cdot x(79);$$

$$\frac{dx(25)}{dt}=\frac{10\cdot\left(x(31)^3+x(40)^3\right)}{1+10\cdot\left(x(31)^3+x(40)^3\right)}\cdot\frac{1}{1+10\cdot x(43)^3}-x(25)\cdot x(80);$$

$$\frac{dx(26)}{dt}=\frac{10\cdot\left(x(20)^3+x(27)^3+x(30)^3+x(32)^3+x(39)^3+x(42)^3\right)}{1+10\cdot\left(x(20)^3+x(27)^3+x(30)^3+x(32)^3+x(39)^3+x(42)^3\right)}-x(26)\cdot x(81);$$

$$\frac{dx(27)}{dt}=\frac{10\cdot\left(x(29)^3+x(35)^3\right)}{1+10\cdot\left(x(29)^3+x(35)^3\right)}-x(27)\cdot x(82);$$

$$\frac{dx(28)}{dt}=\frac{10\cdot\left(x(20)^3+x(26)^3+x(31)^3\right)}{1+10\cdot\left(x(20)^3+x(26)^3+x(31)^3\right)}\cdot\frac{1}{1+10\cdot x(7)^3}-x(28)\cdot x(83);$$

$$\frac{dx(29)}{dt}=\frac{10\cdot\left(x(26)^3+x(31)^3+x(37)^3+x(39)^3+x(40)^3\right)}{1+10\cdot\left(x(26)^3+x(31)^3+x(37)^3+x(39)^3+x(40)^3\right)}\cdot\frac{1}{1+10\cdot x(19)^3}-x(29)\cdot x(84);$$

$$\frac{dx(30)}{dt}=\frac{10\cdot\left(x(20)^3+x(21)^3+x(26)^3+x(28)^3+x(31)^3+x(40)^3\right)}{1+10\cdot\left(x(20)^3+x(21)^3+x(26)^3+x(28)^3+x(31)^3+x(40)^3\right)}\cdot\frac{1}{1+10\cdot x(19)^3}-x(30)\cdot x(85);$$

$$\frac{dx(31)}{dt}=\frac{10\cdot x(20)^3}{1+10\cdot x(20)^3}\cdot -x(31)\cdot x(86);$$

$$\frac{dx(32)}{dt}=\frac{10\cdot\left(x(30)^3+x(35)^3\right)}{1+10\cdot\left(x(30)^3+x(35)^3\right)}\cdot\frac{1}{1+10\cdot x(7)^3}-x(32)\cdot x(87);$$

$$\frac{dx(33)}{dt}=\frac{10\cdot\left(x(20)^3+x(28)^3+x(42)^3\right)}{1+10\cdot\left(x(20)^3+x(28)^3+x(42)^3\right)}\cdot\frac{1}{1+10\cdot x(19)^3}-x(33)\cdot x(88);$$

$$\frac{dx(34)}{dt}=\frac{10\cdot\left(x(25)^3+x(40)^3+x(55)^3\right)}{1+10\cdot\left(x(25)^3+x(40)^3+x(55)^3\right)}\cdot -x(34)\cdot x(89);$$

$$\frac{dx(35)}{dt}=\frac{10\cdot x(29)^3}{1+10\cdot x(29)^3}\cdot\frac{1}{1+10\cdot\left(x(36)^3+x(42)^3\right)}-x(35)\cdot x(90);$$

$$\frac{dx(36)}{dt}=\frac{10\cdot\left(x(29)^3+x(33)^3+x(35)^3\right)}{1+10\cdot\left(x(29)^3+x(33)^3+x(35)^3\right)}\cdot\frac{1}{1+10\cdot\left(x(18)^3+x(30)^3+x(37)^3+x(39)^3+x(40)^3\right)}-x(36)\cdot x(91);$$

$$\frac{dx(37)}{dt}=\frac{10\cdot\left(x(29)^3+x(33)^3+x(35)^3\right)}{1+10\cdot\left(x(29)^3+x(33)^3+x(35)^3\right)}\cdot\frac{1}{1+10\cdot x(38)^3}-x(37)\cdot x(92);$$

$$\frac{dx(38)}{dt}=\frac{10\cdot\left(x(18)^3+x(37)^3\right)}{1+10\cdot\left(x(18)^3+x(37)^3\right)}\cdot\frac{1}{1+10\cdot\left(x(38)^3+x(41)^3\right)}-x(38)\cdot x(93);$$

$$\frac{dx(39)}{dt}=\frac{10\cdot\left(x(29)^3+x(35)^3\right)}{1+10\cdot\left(x(29)^3+x(35)^3\right)}\cdot\frac{1}{1+10\cdot x(39)^3}-x(39)\cdot x(94);$$

$$\frac{dx(40)}{dt}=\frac{10\cdot\left(x(20)^3+x(32)^3+x(37)^3+x(53)^3\right)}{1+10\cdot\left(x(20)^3+x(32)^3+x(37)^3+x(53)^3\right)}\cdot\frac{1}{1+10\cdot\left(x(35)^3+x(48)^3+x(55)^3\right)}-x(40)\cdot x(95);$$

$$\frac{dx(41)}{dt}=\frac{10\cdot x(39)^3}{1+10\cdot x(39)^3}\cdot\frac{1}{1+10\cdot x(38)^3}-x(41)\cdot x(96);$$

$$\frac{dx(42)}{dt}=\frac{10\cdot\left(x(31)^3+x(34)^3+x(38)^3\right)}{1+10\cdot\left(x(31)^3+x(34)^3+x(38)^3\right)}\cdot\frac{1}{1+10\cdot x(40)^3}-x(42)\cdot x(97);$$

$$\frac{dx(43)}{dt}=\frac{1}{1+10\cdot\left(x(30)^3+x(35)^3\right)}-x(43)\cdot x(98);$$

$$\frac{dx(44)}{dt}=\frac{10\cdot\left(x(50)^3+x(54)^3\right)}{1+10\cdot\left(x(50)^3+x(54)^3\right)}\cdot\frac{1}{1+10\cdot\left(x(23)^3+x(32)^3+x(39)^3\right)}-x(44)\cdot x(99);$$

$$\frac{dx(45)}{dt}=\frac{10\cdot\left(x(22)^3+x(25)^3+x(45)^3+x(49)^3+x(50)^3+x(55)^3\right)}{1+10\cdot\left(x(22)^3+x(25)^3+x(45)^3+x(49)^3+x(50)^3+x(55)^3\right)}\cdot\frac{1}{1+10\cdot x(40)^3}-x(45)\cdot x(100);$$

$$\frac{dx(46)}{dt}=\frac{10\cdot x(47)^3}{1+10\cdot x(47)^3}-x(46)\cdot x(101);$$

$$\frac{dx(47)}{dt}=\frac{10\cdot x(23)^3}{1+10\cdot x(23)^3}\cdot\frac{1}{1+10\cdot x(30)^3}-x(47)\cdot x(102);$$

$$\frac{dx(48)}{dt}=\frac{10\cdot x(25)^3}{1+10\cdot x(25)^3}\cdot\frac{1}{1+10\cdot x(55)^3}-x(48)\cdot x(103);$$

$$\frac{dx(49)}{dt}=\frac{10\cdot\left(x(24)^3+x(50)^3\right)}{1+10\cdot\left(x(24)^3+x(50)^3\right)}-x(49)\cdot x(104);$$

$$\frac{dx(50)}{dt}=\frac{10\cdot\left(x(45)^3+x(48)^3+x(49)^3\right)}{1+10\cdot\left(x(45)^3+x(48)^3+x(49)^3\right)}\cdot\frac{1}{1+10\cdot\left(x(7)^3+x(35)^3+x(42)^3+x(55)^3\right)}-x(50)\cdot x(105);$$

$$\frac{dx(51)}{dt}=\frac{10\cdot x(25)^3}{1+10\cdot x(25)^3}\cdot\frac{1}{1+10\cdot x(42)^3}-x(51)\cdot x(106);$$

$$\frac{dx(52)}{dt}=\frac{10\cdot\left(x(45)^3+x(53)^3\right)}{1+10\cdot\left(x(45)^3+x(53)^3\right)}\cdot\frac{1}{1+10\cdot x(54)^3}-x(52)\cdot x(107);$$

$$\frac{dx(53)}{dt}=\frac{1}{1+10\cdot\left(x(39)^3+x(45)^3\right)}-x(53)\cdot x(108);$$

$$\frac{dx(54)}{dt}=\frac{10\cdot\left(x(24)^3+x(45)^3\right)}{1+10\cdot\left(\left(x(24)^3+x(45)^3\right)\right)}\cdot\frac{1}{1+10\cdot x(51)^3}-x(54)\cdot x(109);$$

$$\frac{dx(55)}{dt}=\frac{10\cdot\left(x(23)^3+x(25)^3+x(42)^3+x(55)^3\right)}{1+10\cdot\left(x(23)^3+x(25)^3+x(42)^3+x(55)^3\right)}\cdot\frac{1}{1+10\cdot\left(x(5)^3+x(45)^3\right)}-x(55)\cdot x(110);$$

$$\frac{dx(56)}{dt}=\frac{3}{2}\left[\left(\frac{10\cdot\left(x(3)^3+x(31)^3\right)}{1+10\cdot\left(x(3)^3+x(31)^3\right)}\cdot\frac{1}{1+10\cdot\left(x(5)^3+x(40)^3\right)}-x(1)\cdot x(56)\right)^2+\left(2\cdot\left(\frac{10\cdot\left(x(3)^3+x(31)^3\right)}{1+10\cdot\left(x(3)^3+x(31)^3\right)}\cdot\frac{1}{1+10\cdot\left(x(5)^3+x(40)^3\right)}-x(1)\cdot x(56)\right)\cdot x(56)x(1)\right)\right];$$

$$\frac{dx(57)}{dt}=\frac{3}{2}\left[\left(\frac{10\cdot\left(x(3)^3+x(4)^3\right)}{1+10\cdot\left(x(3)^3+x(4)^3\right)}\cdot\frac{1}{1+10\cdot\left(x(5)^3+x(6)^3+x(19)^3\right)}-x(2)\cdot x(57)\right)^2+\left(2\cdot\left(\frac{10\cdot\left(x(3)^3+x(4)^3\right)}{1+10\cdot\left(x(3)^3+x(4)^3\right)}\cdot\frac{1}{1+10\cdot\left(x(5)^3+x(6)^3+x(19)^3\right)}-x(2)\cdot x(57)\right)\cdot x(57)x(2)\right)\right];$$

$$\frac{dx(58)}{dt}=\frac{3}{2}\left[\left(\frac{10\cdot\left(x(4)^3+x(22)^3+x(23)^3+x(29)^3+x(30)^3+x(32)^3+x(33)^3+x(35)^3\right)}{1+10\cdot\left(x(4)^3+x(22)^3+x(23)^3+x(29)^3+x(30)^3+x(32)^3+x(33)^3+x(35)^3\right)}\cdot\frac{1}{1+10\cdot\left(x(5)^3+x(7)^3+x(40)^3+x(41)^3\right)}-x(3)\cdot x(58)\right)^2 \right.$$
$$\left.+\left(2\cdot\left(\frac{10\cdot\left(x(4)^3+x(22)^3+x(23)^3+x(29)^3+x(30)^3+x(32)^3+x(33)^3+x(35)^3\right)}{1+10\cdot\left(x(4)^3+x(22)^3+x(23)^3+x(29)^3+x(30)^3+x(32)^3+x(33)^3+x(35)^3\right)}\cdot\frac{1}{1+10\cdot\left(x(5)^3+x(7)^3+x(40)^3+x(41)^3\right)}-x(3)\cdot x(58)\right)\cdot x(58)x(3)\right)\right];$$

$$\frac{dx(59)}{dt}=\frac{3}{2}\left[\left(\frac{10\cdot\left(x(3)^3+x(4)^3\right)}{1+10\cdot\left(x(3)^3+x(4)^3\right)}\cdot\frac{1}{1+10\cdot\left(x(5)^3+x(8)^3\right)}-x(4)\cdot x(59)\right)^2+\left(2\cdot\left(\frac{10\cdot\left(x(3)^3+x(4)^3\right)}{1+10\cdot\left(x(3)^3+x(4)^3\right)}\cdot\frac{1}{1+10\cdot\left(x(5)^3+x(8)^3\right)}-x(4)\cdot x(59)\right)\cdot x(59)x(4)\right)\right];$$

$$\frac{dx(60)}{dt}=\frac{3}{2}\left[\left(\frac{10\cdot\left(x(4)^3+x(7)^3+x(32)^3+x(33)^3+x(40)^3+x(41)^3+x(45)^3+x(50)^3+x(54)^3\right)}{1+10\cdot\left(x(4)^3+x(7)^3+x(32)^3+x(33)^3+x(40)^3+x(41)^3+x(45)^3+x(50)^3+x(54)^3\right)}\cdot\frac{1}{1+10\cdot\left(x(3)^3+x(30)^3\right)}-x(5)\cdot x(60)\right)^2\right.$$
$$\left.+\left(2\cdot\left(\frac{10\cdot\left(x(4)^3+x(7)^3+x(32)^3+x(33)^3+x(40)^3+x(41)^3+x(45)^3+x(50)^3+x(54)^3\right)}{1+10\cdot\left(x(4)^3+x(7)^3+x(32)^3+x(33)^3+x(40)^3+x(41)^3+x(45)^3+x(50)^3+x(54)^3\right)}\cdot\frac{1}{1+10\cdot\left(x(3)^3+x(30)^3\right)}-x(5)\cdot x(60)\right)\cdot x(60)x(5)\right)\right];$$

$$\frac{dx(61)}{dt}=\frac{3}{2}\left[\left(\frac{10\cdot\left(x(19)^3+x(21)^3+x(40)^3+x(41)^3+x(55)^3\right)}{1+10\cdot\left(x(19)^3+x(21)^3+x(40)^3+x(41)^3+x(55)^3\right)}\cdot\frac{1}{1+10\cdot\left(x(2)^3+x(3)^3+x(30)^3+x(33)^3\right)}-x(6)\cdot x(61)\right)^2\right.$$
$$\left.+\left(2\cdot\left(\frac{10\cdot\left(x(19)^3+x(21)^3+x(40)^3+x(41)^3+x(55)^3\right)}{1+10\cdot\left(x(19)^3+x(21)^3+x(40)^3+x(41)^3+x(55)^3\right)}\cdot\frac{1}{1+10\cdot\left(x(2)^3+x(3)^3+x(30)^3+x(33)^3\right)}-x(6)\cdot x(61)\right)\cdot x(61)x(6)\right)\right];$$

$$\frac{dx(62)}{dt}=\frac{3}{2}\left[\left(\frac{10\cdot\left(x(3)^3+x(4)^3+x(10)^3+x(19)^3+x(35)^3\right)}{1+10\cdot\left(x(3)^3+x(4)^3+x(10)^3+x(19)^3+x(35)^3\right)}\cdot\frac{1}{1+10\cdot\left(x(24)^3+x(30)^3\right)}-x(7)\cdot x(62)\right)^2\right.$$
$$\left.+\left(2\cdot\left(\frac{10\cdot\left(x(3)^3+x(4)^3+x(10)^3+x(19)^3+x(35)^3\right)}{1+10\cdot\left(x(3)^3+x(4)^3+x(10)^3+x(19)^3+x(35)^3\right)}\cdot\frac{1}{1+10\cdot\left(x(24)^3+x(30)^3\right)}-x(7)\cdot x(62)\right)\cdot x(62)x(7)\right)\right];$$

$$\frac{dx(63)}{dt}=\frac{3}{2}\left[\left(\frac{1}{1+10\cdot\left(x(1)^3+x(2)^3+x(9)^3\right)}-x(8)\cdot x(63)\right)^2+\left(2\cdot\left(\frac{1}{1+10\cdot\left(x(1)^3+x(2)^3+x(9)^3\right)}-x(8)\cdot x(63)\right)\cdot x(63)x(8)\right)\right];$$

$$\frac{dx(64)}{dt}=\frac{3}{2}\left[\left(\frac{10\cdot\left(x(10)^3+x(11)^3\right)}{1+10\cdot\left(x(10)^3+x(11)^3\right)}\cdot\frac{1}{1+10\cdot\left(x(5)^3+x(12)^3\right)}-x(9)\cdot x(64)\right)^2+\left(2\cdot\left(\frac{10\cdot\left(x(10)^3+x(11)^3\right)}{1+10\cdot\left(x(10)^3+x(11)^3\right)}\cdot\frac{1}{1+10\cdot\left(x(5)^3+x(12)^3\right)}-x(9)\cdot x(64)\right)\cdot x(64)x(9)\right)\right];$$

$$\frac{dx(65)}{dt}=\frac{3}{2}\left[\begin{array}{l}\left(\frac{10\cdot\left(x(3)^3+x(4)^3+x(15)^3+x(16)^3\right)}{1+10\cdot\left(x(3)^3+x(4)^3+x(15)^3+x(16)^3\right)}\cdot\frac{1}{1+10\cdot\left(x(12)^3+x(30)^3\right)}-x(10)\cdot x(65)\right)^2\\+\left(2\cdot\left(\frac{10\cdot\left(x(3)^3+x(4)^3+x(15)^3+x(16)^3\right)}{1+10\cdot\left(x(3)^3+x(4)^3+x(15)^3+x(16)^3\right)}\cdot\frac{1}{1+10\cdot\left(x(12)^3+x(30)^3\right)}-x(10)\cdot x(65)\right)\cdot x(65)x(10)\right)\end{array}\right];$$

$$\frac{dx(66)}{dt}=\frac{3}{2}\left[\left(\frac{10\cdot\left(x(18)^3+x(37)^3+x(39)^3\right)}{1+10\cdot\left(x(18)^3+x(37)^3+x(39)^3\right)}\cdot\frac{1}{1+10\cdot x(35)^3}-x(11)\cdot x(66)\right)^2+\left(2\cdot\left(\frac{10\cdot\left(x(18)^3+x(37)^3+x(39)^3\right)}{1+10\cdot\left(x(18)^3+x(37)^3+x(39)^3\right)}\cdot\frac{1}{1+10\cdot x(35)^3}-x(11)\cdot x(66)\right)\cdot x(66)x(11)\right)\right];$$

$$\frac{dx(67)}{dt}=\frac{3}{2}\left[\left(\frac{10\cdot\left(x(30)^3+x(35)^3\right)}{1+10\cdot\left(x(30)^3+x(35)^3\right)}\cdot\frac{1}{1+10\cdot x(10)^3}-x(12)\cdot x(67)\right)^2+\left(2\cdot\left(\frac{10\cdot\left(x(30)^3+x(35)^3\right)}{1+10\cdot\left(x(30)^3+x(35)^3\right)}\cdot\frac{1}{1+10\cdot x(10)^3}-x(12)\cdot x(67)\right)\cdot x(67)x(12)\right)\right];$$

$$\frac{dx(68)}{dt}=\frac{3}{2}\left[\begin{array}{l}\left(\frac{10\cdot\left(x(23)^3+x(26)^3+x(27)^3+x(33)^3+x(35)^3\right)}{1+10\cdot\left(x(23)^3+x(26)^3+x(27)^3+x(33)^3+x(35)^3\right)}\cdot\frac{1}{1+10\cdot\left(x(3)^3+x(7)^3+x(15)^3+x(16)^3+x(40)^3\right)}-x(13)\cdot x(68)\right)^2\\+\left(2\cdot\left(\frac{10\cdot\left(x(23)^3+x(26)^3+x(27)^3+x(33)^3+x(35)^3\right)}{1+10\cdot\left(x(23)^3+x(26)^3+x(27)^3+x(33)^3+x(35)^3\right)}\cdot\frac{1}{1+10\cdot\left(x(3)^3+x(7)^3+x(15)^3+x(16)^3+x(40)^3\right)}-x(13)\cdot x(68)\right)\cdot x(68)x(13)\right)\end{array}\right];$$

$$\frac{dx(69)}{dt}=\frac{3}{2}\left[\left(\frac{10\cdot\left(x(31)^3+x(35)^3\right)}{1+10\cdot\left(x(31)^3+x(35)^3\right)}\cdot\frac{1}{1+10\cdot\left(x(3)^3+x(16)^3\right)}-x(14)\cdot x(69)\right)^2+\left(2\cdot\left(\frac{10\cdot\left(x(31)^3+x(35)^3\right)}{1+10\cdot\left(x(31)^3+x(35)^3\right)}\cdot\frac{1}{1+10\cdot\left(x(3)^3+x(16)^3\right)}-x(14)\cdot x(69)\right)\cdot x(69)x(14)\right)\right];$$

$$\frac{dx(70)}{dt}=\frac{3}{2}\left[\left(\frac{10\cdot x(11)^3}{1+10\cdot x(11)^3}\cdot\frac{1}{1+10\cdot x(13)^3}-x(15)\cdot x(70)\right)^2+\left(2\cdot\left(\frac{10\cdot x(11)^3}{1+10\cdot x(11)^3}\cdot\frac{1}{1+10\cdot x(13)^3}-x(15)\cdot x(70)\right)\cdot x(70)x(15)\right)\right];$$

$$\frac{dx(71)}{dt}=\frac{3}{2}\left[\left(\frac{10\cdot x(7)^3}{1+10\cdot x(7)^3}\cdot\frac{1}{1+10\cdot\left(x(30)^3+x(33)^3\right)}-x(16)\cdot x(71)\right)^2+\left(2\cdot\left(\frac{10\cdot x(7)^3}{1+10\cdot x(7)^3}\cdot\frac{1}{1+10\cdot\left(x(30)^3+x(33)^3\right)}-x(16)\cdot x(71)\right)\cdot x(71)x(16)\right)\right];$$

$$\frac{dx(72)}{dt}=\frac{3}{2}\left[\left(\frac{10\cdot\left(x(3)^3+x(7)^3+x(15)^3\right)}{1+10\cdot\left(x(3)^3+x(7)^3+x(15)^3\right)}\cdot\frac{1}{1+10\cdot\left(x(13)^3+x(14)^3\right)}-x(17)\cdot x(72)\right)^2+\left(2\cdot\left(\frac{10\cdot\left(x(3)^3+x(7)^3+x(15)^3\right)}{1+10\cdot\left(x(3)^3+x(7)^3+x(15)^3\right)}\cdot\frac{1}{1+10\cdot\left(x(13)^3+x(14)^3\right)}-x(17)\cdot x(72)\right)\cdot x(72)x(17)\right)\right];$$

$$\frac{dx(73)}{dt}=\frac{3}{2}\left[\left(\frac{10\cdot\left(x(3)^3+x(35)^3+x(41)^3\right)}{1+10\cdot\left(x(3)^3+x(35)^3+x(41)^3\right)}\cdot\frac{1}{1+10\cdot\left(x(28)^3\right)}-x(18)\cdot x(73)\right)^2+\left(2\cdot\left(\frac{10\cdot\left(x(3)^3+x(35)^3+x(41)^3\right)}{1+10\cdot\left(x(3)^3+x(35)^3+x(41)^3\right)}\cdot\frac{1}{1+10\cdot\left(x(28)^3\right)}-x(18)\cdot x(73)\right)\cdot x(73)x(18)\right)\right];$$

$$\frac{dx(74)}{dt}=\frac{3}{2}\left[\left(\frac{1}{1+10\cdot\left(x(22)^3+x(30)^3+x(35)^3\right)}-x(19)\cdot x(74)\right)^2+\left(2\cdot\left(\frac{1}{1+10\cdot\left(x(22)^3+x(30)^3+x(35)^3\right)}-x(19)\cdot x(74)\right)\cdot x(74)x(19)\right)\right];$$

$$\frac{dx(75)}{dt}=\frac{3}{2}\left[\left(\frac{10\cdot x(31)^3}{1+10\cdot x(31)^3}-x(20)\cdot x(75)\right)^2+\left(2\cdot\left(\frac{10\cdot x(31)^3}{1+10\cdot x(31)^3}-x(20)\cdot x(75)\right)\cdot x(75)x(20)\right)\right];$$

$$\frac{dx(76)}{dt}=\frac{3}{2}\left[\left(\frac{1}{1+10\cdot\left(x(9)^3+x(20)^3+x(39)^3\right)}-x(21)\cdot x(76)\right)^2+\left(2\cdot\left(\frac{1}{1+10\cdot\left(x(9)^3+x(20)^3+x(39)^3\right)}-x(21)\cdot x(76)\right)\cdot x(76)x(21)\right)\right];$$

$$\frac{dx(77)}{dt}=\frac{3}{2}\left[\left(\frac{10\cdot x(31)^3}{1+10\cdot x(31)^3}\cdot\frac{1}{1+10\cdot\left(x(21)^3+x(43)^3+x(55)^3\right)}-x(22)\cdot x(77)\right)^2+\left(2\cdot\left(\frac{10\cdot x(31)^3}{1+10\cdot x(31)^3}\cdot\frac{1}{1+10\cdot\left(x(21)^3+x(43)^3+x(55)^3\right)}-x(22)\cdot x(77)\right)\cdot x(77)x(22)\right)\right];$$

$$\frac{dx(78)}{dt}=\frac{3}{2}\left[\left(\frac{10\cdot x(55)^3}{1+10\cdot x(55)^3}\cdot\frac{1}{1+10\cdot\left(x(43)^3+x(45)^3\right)}-x(23)\cdot x(78)\right)^2+\left(2\cdot\left(\frac{10\cdot x(55)^3}{1+10\cdot x(55)^3}\cdot\frac{1}{1+10\cdot\left(x(43)^3+x(45)^3\right)}-x(23)\cdot x(78)\right)\cdot x(78)x(23)\right)\right];$$

$$\frac{dx(79)}{dt}=\frac{3}{2}\left[\begin{array}{l}\left(\frac{10\cdot\left(x(35)^3+x(42)^3+x(45)^3\right)}{1+10\cdot\left(x(35)^3+x(42)^3+x(45)^3\right)}\cdot\frac{1}{1+10\cdot\left(x(21)^3+x(44)^3+x(52)^3\right)}-x(24)\cdot x(79)\right)^2\\+\left(2\cdot\left(\frac{10\cdot\left(x(35)^3+x(42)^3+x(45)^3\right)}{1+10\cdot\left(x(35)^3+x(42)^3+x(45)^3\right)}\cdot\frac{1}{1+10\cdot\left(x(21)^3+x(44)^3+x(52)^3\right)}-x(24)\cdot x(79)\right)\cdot x(79)x(24)\right)\end{array}\right];$$

$$\frac{dx(80)}{dt}=\frac{3}{2}\left[\left(\frac{10\cdot\left(x(31)^3+x(40)^3\right)}{1+10\cdot\left(x(31)^3+x(40)^3\right)}\cdot\frac{1}{1+10\cdot x(43)^3}-x(25)\cdot x(80)\right)^2+\left(2\cdot\left(\frac{10\cdot\left(x(31)^3+x(40)^3\right)}{1+10\cdot\left(x(31)^3+x(40)^3\right)}\cdot\frac{1}{1+10\cdot x(43)^3}-x(25)\cdot x(80)\right)\cdot x(80)x(25)\right)\right];$$

$$\frac{dx(81)}{dt}=\frac{3}{2}\left[\begin{array}{l}\left(\frac{10\cdot\left(x(20)^3+x(27)^3+x(30)^3+x(32)^3+x(39)^3+x(42)^3\right)}{1+10\cdot\left(x(20)^3+x(27)^3+x(30)^3+x(32)^3+x(39)^3+x(42)^3\right)}-x(26)\cdot x(81)\right)^2\\+\left(2\cdot\left(\frac{10\cdot\left(x(20)^3+x(27)^3+x(30)^3+x(32)^3+x(39)^3+x(42)^3\right)}{1+10\cdot\left(x(20)^3+x(27)^3+x(30)^3+x(32)^3+x(39)^3+x(42)^3\right)}-x(26)\cdot x(81)\right)\cdot x(81)x(26)\right)\end{array}\right];$$

$$\frac{dx(82)}{dt}=\frac{3}{2}\left[\left(\frac{10\cdot\left(x(29)^3+x(35)^3\right)}{1+10\cdot\left(x(29)^3+x(35)^3\right)}-x(27)\cdot x(82)\right)^2+\left(2\cdot\left(\frac{10\cdot\left(x(29)^3+x(35)^3\right)}{1+10\cdot\left(x(29)^3+x(35)^3\right)}-x(27)\cdot x(82)\right)\cdot x(82)x(27)\right)\right];$$

$$\frac{dx(83)}{dt}=\frac{3}{2}\left[\left(\frac{10\cdot\left(x(20)^3+x(26)^3+x(31)^3\right)}{1+10\cdot\left(x(20)^3+x(26)^3+x(31)^3\right)}\cdot\frac{1}{1+10\cdot x(7)^3}-x(28)\cdot x(83)\right)^2+\left(2\cdot\left(\frac{10\cdot\left(x(20)^3+x(26)^3+x(31)^3\right)}{1+10\cdot\left(x(20)^3+x(26)^3+x(31)^3\right)}\cdot\frac{1}{1+10\cdot x(7)^3}-x(28)\cdot x(83)\right)\cdot x(83)x(28)\right)\right];$$

$$\frac{dx(84)}{dt}=\frac{3}{2}\left[\begin{array}{l}\left(\frac{10\cdot\left(x(26)^3+x(31)^3+x(37)^3+x(39)^3+x(40)^3\right)}{1+10\cdot\left(x(26)^3+x(31)^3+x(37)^3+x(39)^3+x(40)^3\right)}\cdot\frac{1}{1+10\cdot x(19)^3}-x(29)\cdot x(84)\right)^2\\+\left(2\cdot\left(\frac{10\cdot\left(x(26)^3+x(31)^3+x(37)^3+x(39)^3+x(40)^3\right)}{1+10\cdot\left(x(26)^3+x(31)^3+x(37)^3+x(39)^3+x(40)^3\right)}\cdot\frac{1}{1+10\cdot x(19)^3}-x(29)\cdot x(84)\right)\cdot x(84)x(29)\right)\end{array}\right];$$

$$\frac{dx(85)}{dt}=\frac{3}{2}\left[\begin{array}{l}\left(\frac{10\cdot\left(x(20)^3+x(21)^3+x(26)^3+x(28)^3+x(31)^3+x(40)^3\right)}{1+10\cdot\left(x(20)^3+x(21)^3+x(26)^3+x(28)^3+x(31)^3+x(40)^3\right)}\cdot\frac{1}{1+10\cdot x(19)^3}-x(30)\cdot x(85)\right)^2\\+\left(2\cdot\left(\frac{10\cdot\left(x(20)^3+x(21)^3+x(26)^3+x(28)^3+x(31)^3+x(40)^3\right)}{1+10\cdot\left(x(20)^3+x(21)^3+x(26)^3+x(28)^3+x(31)^3+x(40)^3\right)}\cdot\frac{1}{1+10\cdot x(19)^3}-x(30)\cdot x(85)\right)\cdot x(85)x(30)\right)\end{array}\right];$$

$$\frac{dx(86)}{dt}=\frac{3}{2}\left[\left(\frac{10\cdot x(20)^3}{1+10\cdot x(20)^3}\cdot-x(31)\cdot x(86)\right)^2+\left(2\cdot\left(\frac{10\cdot x(20)^3}{1+10\cdot x(20)^3}\cdot-x(31)\cdot x(86)\right)\cdot x(86)x(31)\right)\right];$$

$$\frac{dx(87)}{dt}=\frac{3}{2}\left[\left(\frac{10\cdot\left(x(30)^3+x(35)^3\right)}{1+10\cdot\left(x(30)^3+x(35)^3\right)}\cdot\frac{1}{1+10\cdot x(7)^3}-x(32)\cdot x(87)\right)^2+\left(2\cdot\left(\frac{10\cdot\left(x(30)^3+x(35)^3\right)}{1+10\cdot\left(x(30)^3+x(35)^3\right)}\cdot\frac{1}{1+10\cdot x(7)^3}-x(32)\cdot x(87)\right)\cdot x(87)x(32)\right)\right];$$

$$\frac{dx(88)}{dt}=\frac{3}{2}\left[\left(\frac{10\cdot\left(x(20)^3+x(28)^3+x(42)^3\right)}{1+10\cdot\left(x(20)^3+x(28)^3+x(42)^3\right)}\cdot\frac{1}{1+10\cdot x(19)^3}-x(33)\cdot x(88)\right)^2+\left(2\cdot\left(\frac{10\cdot\left(x(20)^3+x(28)^3+x(42)^3\right)}{1+10\cdot\left(x(20)^3+x(28)^3+x(42)^3\right)}\cdot\frac{1}{1+10\cdot x(19)^3}-x(33)\cdot x(88)\right)\cdot x(88)x(33)\right)\right];$$

$$\frac{dx(89)}{dt}=\frac{3}{2}\left[\left(\frac{10\cdot\left(x(25)^3+x(40)^3+x(55)^3\right)}{1+10\cdot\left(x(25)^3+x(40)^3+x(55)^3\right)}\cdot-x(34)\cdot x(89)\right)^2+\left(2\cdot\left(\frac{10\cdot\left(x(25)^3+x(40)^3+x(55)^3\right)}{1+10\cdot\left(x(25)^3+x(40)^3+x(55)^3\right)}\cdot-x(34)\cdot x(89)\right)\cdot x(89)x(34)\right)\right];$$

$$\frac{dx(90)}{dt}=\frac{3}{2}\left[\left(\frac{10\cdot x(29)^3}{1+10\cdot x(29)^3}\cdot\frac{1}{1+10\cdot\left(x(36)^3+x(42)^3\right)}-x(35)\cdot x(90)\right)^2+\left(2\cdot\left(\frac{10\cdot x(29)^3}{1+10\cdot x(29)^3}\cdot\frac{1}{1+10\cdot\left(x(36)^3+x(42)^3\right)}-x(35)\cdot x(90)\right)\cdot x(90)x(35)\right)\right];$$

$$\frac{dx(91)}{dt}=\frac{3}{2}\left[\begin{array}{l}\left(\frac{10\cdot\left(x(29)^3+x(33)^3+x(35)^3\right)}{1+10\cdot\left(x(29)^3+x(33)^3+x(35)^3\right)}\cdot\frac{1}{1+10\cdot\left(x(18)^3+x(30)^3+x(37)^3+x(39)^3+x(40)^3\right)}-x(36)\cdot x(91)\right)^2\\+\left(2\cdot\left(\frac{10\cdot\left(x(29)^3+x(33)^3+x(35)^3\right)}{1+10\cdot\left(x(29)^3+x(33)^3+x(35)^3\right)}\cdot\frac{1}{1+10\cdot\left(x(18)^3+x(30)^3+x(37)^3+x(39)^3+x(40)^3\right)}-x(36)\cdot x(91)\right)\cdot x(91)x(36)\right)\end{array}\right];$$

$$\frac{dx(92)}{dt}=\frac{3}{2}\left[\left(\frac{10\cdot\left(x(29)^3+x(33)^3+x(35)^3\right)}{1+10\cdot\left(x(29)^3+x(33)^3+x(35)^3\right)}\cdot\frac{1}{1+10\cdot x(38)^3}-x(37)\cdot x(92)\right)^2+\left(2\cdot\left(\frac{10\cdot\left(x(29)^3+x(33)^3+x(35)^3\right)}{1+10\cdot\left(x(29)^3+x(33)^3+x(35)^3\right)}\cdot\frac{1}{1+10\cdot x(38)^3}-x(37)\cdot x(92)\right)\cdot x(92)x(37)\right)\right];$$

$$\frac{dx(93)}{dt}=\frac{3}{2}\left[\left(\frac{10\cdot\left(x(18)^3+x(37)^3\right)}{1+10\cdot\left(x(18)^3+x(37)^3\right)}\cdot\frac{1}{1+10\cdot\left(x(38)^3+x(41)^3\right)}-x(38)\cdot x(93)\right)^2+\left(2\cdot\left(\frac{10\cdot\left(x(18)^3+x(37)^3\right)}{1+10\cdot\left(x(18)^3+x(37)^3\right)}\cdot\frac{1}{1+10\cdot\left(x(38)^3+x(41)^3\right)}-x(38)\cdot x(93)\right)\cdot x(93)x(38)\right)\right];$$

$$\frac{dx(94)}{dt}=\frac{3}{2}\left[\left(\frac{10\cdot\left(x(29)^3+x(35)^3\right)}{1+10\cdot\left(x(29)^3+x(35)^3\right)}\cdot\frac{1}{1+10\cdot x(39)^3}-x(39)\cdot x(94)\right)^2+\left(2\cdot\left(\frac{10\cdot\left(x(29)^3+x(35)^3\right)}{1+10\cdot\left(x(29)^3+x(35)^3\right)}\cdot\frac{1}{1+10\cdot x(39)^3}-x(39)\cdot x(94)\right)\cdot x(94)x(39)\right)\right];$$

$$\frac{dx(95)}{dt}=\frac{3}{2}\left[\begin{array}{l}\left(\frac{10\cdot\left(x(20)^3+x(32)^3+x(37)^3+x(53)^3\right)}{1+10\cdot\left(x(20)^3+x(32)^3+x(37)^3+x(53)^3\right)}\cdot\frac{1}{1+10\cdot\left(x(35)^3+x(48)^3+x(55)^3\right)}-x(40)\cdot x(95)\right)^2\\+\left(2\cdot\left(\frac{10\cdot\left(x(20)^3+x(32)^3+x(37)^3+x(53)^3\right)}{1+10\cdot\left(x(20)^3+x(32)^3+x(37)^3+x(53)^3\right)}\cdot\frac{1}{1+10\cdot\left(x(35)^3+x(48)^3+x(55)^3\right)}-x(40)\cdot x(95)\right)\cdot x(95)x(40)\right)\end{array}\right];$$

$$\frac{dx(96)}{dt}=\frac{3}{2}\left[\left(\frac{10\cdot x(39)^3}{1+10\cdot x(39)^3}\cdot\frac{1}{1+10\cdot x(38)^3}-x(41)\cdot x(96)\right)^2+\left(2\cdot\left(\frac{10\cdot x(39)^3}{1+10\cdot x(39)^3}\cdot\frac{1}{1+10\cdot x(38)^3}-x(41)\cdot x(96)\right)\cdot x(96)x(41)\right)\right];$$

$$\frac{dx(97)}{dt}=\frac{3}{2}\left[\left(\frac{10\cdot\left(x(31)^3+x(34)^3+x(38)^3\right)}{1+10\cdot\left(x(31)^3+x(34)^3+x(38)^3\right)}\cdot\frac{1}{1+10\cdot x(40)^3}-x(42)\cdot x(97)\right)^2+\left(2\cdot\left(\frac{10\cdot\left(x(31)^3+x(34)^3+x(38)^3\right)}{1+10\cdot\left(x(31)^3+x(34)^3+x(38)^3\right)}\cdot\frac{1}{1+10\cdot x(40)^3}-x(42)\cdot x(97)\right)\cdot x(97)x(42)\right)\right];$$

$$\frac{dx(98)}{dt}=\frac{3}{2}\left[\left(\frac{1}{1+10\cdot\left(x(30)^3+x(35)^3\right)}-x(43)\cdot x(98)\right)^2+\left(2\cdot\left(\frac{1}{1+10\cdot\left(x(30)^3+x(35)^3\right)}-x(43)\cdot x(98)\right)\cdot x(98)x(43)\right)\right];$$

$$\frac{dx(99)}{dt}=\frac{3}{2}\left[\left(\frac{10\cdot\left(x(50)^3+x(54)^3\right)}{1+10\cdot\left(x(50)^3+x(54)^3\right)}\cdot\frac{1}{1+10\cdot\left(x(23)^3+x(32)^3+x(39)^3\right)}-x(44)\cdot x(99)\right)^2+\left(2\cdot\left(\frac{10\cdot\left(x(50)^3+x(54)^3\right)}{1+10\cdot\left(x(50)^3+x(54)^3\right)}\cdot\frac{1}{1+10\cdot\left(x(23)^3+x(32)^3+x(39)^3\right)}-x(44)\cdot x(99)\right)\cdot x(99)x(44)\right)\right];$$

$$\frac{dx(100)}{dt}=\frac{3}{2}\left[\left(\frac{10\cdot\left(x(22)^3+x(25)^3+x(45)^3+x(49)^3+x(50)^3+x(55)^3\right)}{1+10\cdot\left(x(22)^3+x(25)^3+x(45)^3+x(49)^3+x(50)^3+x(55)^3\right)}\cdot\frac{1}{1+10\cdot x(40)^3}-x(45)\cdot x(100)\right)^2+\left(2\cdot\left(\frac{10\cdot\left(x(22)^3+x(25)^3+x(45)^3+x(49)^3+x(50)^3+x(55)^3\right)}{1+10\cdot\left(x(22)^3+x(25)^3+x(45)^3+x(49)^3+x(50)^3+x(55)^3\right)}\cdot\frac{1}{1+10\cdot x(40)^3}-x(45)\cdot x(100)\right)\cdot x(100)x(45)\right)\right];$$

$$\frac{dx(101)}{dt}=\frac{3}{2}\left[\left(\frac{10\cdot x(47)^3}{1+10\cdot x(47)^3}-x(46)\cdot x(101)\right)^2+\left(2\cdot\left(\frac{10\cdot x(47)^3}{1+10\cdot x(47)^3}-x(46)\cdot x(101)\right)\cdot x(101)x(46)\right)\right];$$

$$\frac{dx(102)}{dt}=\frac{3}{2}\left[\left(\frac{10\cdot x(23)^3}{1+10\cdot x(23)^3}\cdot\frac{1}{1+10\cdot x(30)^3}-x(47)\cdot x(102)\right)^2+\left(2\cdot\left(\frac{10\cdot x(23)^3}{1+10\cdot x(23)^3}\cdot\frac{1}{1+10\cdot x(30)^3}-x(47)\cdot x(102)\right)\cdot x(102)x(47)\right)\right];$$

$$\frac{dx(103)}{dt}=\frac{3}{2}\left[\left(\frac{10\cdot x(25)^3}{1+10\cdot x(25)^3}\cdot\frac{1}{1+10\cdot x(55)^3}-x(48)\cdot x(103)\right)^2+\left(2\cdot\left(\frac{10\cdot x(25)^3}{1+10\cdot x(25)^3}\cdot\frac{1}{1+10\cdot x(55)^3}-x(48)\cdot x(103)\right)\cdot x(103)x(48)\right)\right];$$

$$\frac{dx(104)}{dt}=\frac{3}{2}\left[\left(\frac{10\cdot\left(x(24)^3+x(50)^3\right)}{1+10\cdot\left(x(24)^3+x(50)^3\right)}-x(49)\cdot x(104)\right)^2+\left(2\cdot\left(\frac{10\cdot\left(x(24)^3+x(50)^3\right)}{1+10\cdot\left(x(24)^3+x(50)^3\right)}-x(49)\cdot x(104)\right)\cdot x(104)x(49)\right)\right];$$

$$\frac{dx(105)}{dt}=\frac{3}{2}\left[\left(\frac{10\cdot\left(x(45)^3+x(48)^3+x(49)^3\right)}{1+10\cdot\left(x(45)^3+x(48)^3+x(49)^3\right)}\cdot\frac{1}{1+10\cdot\left(x(7)^3+x(35)^3+x(42)^3+x(55)^3\right)}-x(50)\cdot x(105)\right)^2+\left(2\cdot\left(\frac{10\cdot\left(x(45)^3+x(48)^3+x(49)^3\right)}{1+10\cdot\left(x(45)^3+x(48)^3+x(49)^3\right)}\cdot\frac{1}{1+10\cdot\left(x(7)^3+x(35)^3+x(42)^3+x(55)^3\right)}-x(50)\cdot x(105)\right)\cdot x(105)x(50)\right)\right];$$

$$\frac{dx(106)}{dt}=\frac{3}{2}\left[\left(\frac{10\cdot x(25)^3}{1+10\cdot x(25)^3}\cdot\frac{1}{1+10\cdot x(42)^3}-x(51)\cdot x(106)\right)^2+\left(2\cdot\left(\frac{10\cdot x(25)^3}{1+10\cdot x(25)^3}\cdot\frac{1}{1+10\cdot x(42)^3}-x(51)\cdot x(106)\right)\cdot x(106)x(51)\right)\right];$$

$$\frac{dx(107)}{dt}=\frac{3}{2}\left[\left(\frac{10\cdot\left(x(45)^3+x(53)^3\right)}{1+10\cdot\left(x(45)^3+x(53)^3\right)}\cdot\frac{1}{1+10\cdot x(54)^3}-x(52)\cdot x(107)\right)^2+\left(2\cdot\left(\frac{10\cdot\left(x(45)^3+x(53)^3\right)}{1+10\cdot\left(x(45)^3+x(53)^3\right)}\cdot\frac{1}{1+10\cdot x(54)^3}-x(52)\cdot x(107)\right)\cdot x(107)x(52)\right)\right];$$

$$\frac{dx(108)}{dt}=\frac{3}{2}\left[\left(\frac{1}{1+10\cdot\left(x(39)^3+x(45)^3\right)}-x(53)\cdot x(108)\right)^2+\left(2\cdot\left(\frac{1}{1+10\cdot\left(x(39)^3+x(45)^3\right)}-x(53)\cdot x(108)\right)\cdot x(108)x(53)\right)\right];$$

$$\frac{dx(109)}{dt}=\frac{3}{2}\left[\left(\frac{10\cdot\left(x(24)^3+x(45)^3\right)}{1+10\cdot\left(\left(x(24)^3+x(45)^3\right)\right)}\cdot\frac{1}{1+10\cdot x(51)^3}-x(54)\cdot x(109)\right)^2+\left(2\cdot\left(\frac{10\cdot\left(x(24)^3+x(45)^3\right)}{1+10\cdot\left(\left(x(24)^3+x(45)^3\right)\right)}\cdot\frac{1}{1+10\cdot x(51)^3}-x(54)\cdot x(109)\right)\cdot x(109)x(54)\right)\right];$$

$$\frac{dx(110)}{dt}=\frac{3}{2}\left[\begin{aligned}&\left(\frac{10\cdot\left(x(23)^3+x(25)^3+x(42)^3+x(55)^3\right)}{1+10\cdot\left(x(23)^3+x(25)^3+x(42)^3+x(55)^3\right)}\cdot\frac{1}{1+10\cdot\left(x(5)^3+x(45)^3\right)}-x(55)\cdot x(110)\right)^2\\&+\left(2\cdot\left(\frac{10\cdot\left(x(23)^3+x(25)^3+x(42)^3+x(55)^3\right)}{1+10\cdot\left(x(23)^3+x(25)^3+x(42)^3+x(55)^3\right)}\cdot\frac{1}{1+10\cdot\left(x(5)^3+x(45)^3\right)}-x(55)\cdot x(110)\right)\cdot x(110)x(55)\right)\end{aligned}\right].$$

C. Analyze steady states and microarray data

Steady State Data and Ruijin Hospital Samples

1: In the absence of network intervention, the simulation yield steady states for the network, target:0.

2: Microarray Data from Ruijin Hospital's Samples, GEO: GSE54129.

Analysis Process for the four Data Sets

We obtained microarray data from samples collected at Ruijin Hospital. To facilitate analysis, the data underwent Z-score normalization, transforming it into a standardized form with a mean of 0 and a standard deviation of 1. This normalization method eliminates inherent size differences between genes, enabling meaningful comparisons on a common scale. It ensures compatibility for comparing initially dissimilar and non-directly comparable values. This normalization step establishes the foundation for subsequent comparisons with gene data from Ruijin Hospital.

1. Analysis of All Steady States:

To analyze the simulation data, we standardized all the data with a mean of 0 and a standard deviation of 1. Using Principal Component Analysis (PCA), we identified the top contributing components, PC1 and PC2 (as illustrated in Figure S2). Subsequently, we performed hierarchical clustering on the stable points of the network, leading to the identification of three distinct clusters: A1, A2, and A3 (as depicted in Figure 3A in the main text). This finding suggests the presence of three major degenerate attractor basins.

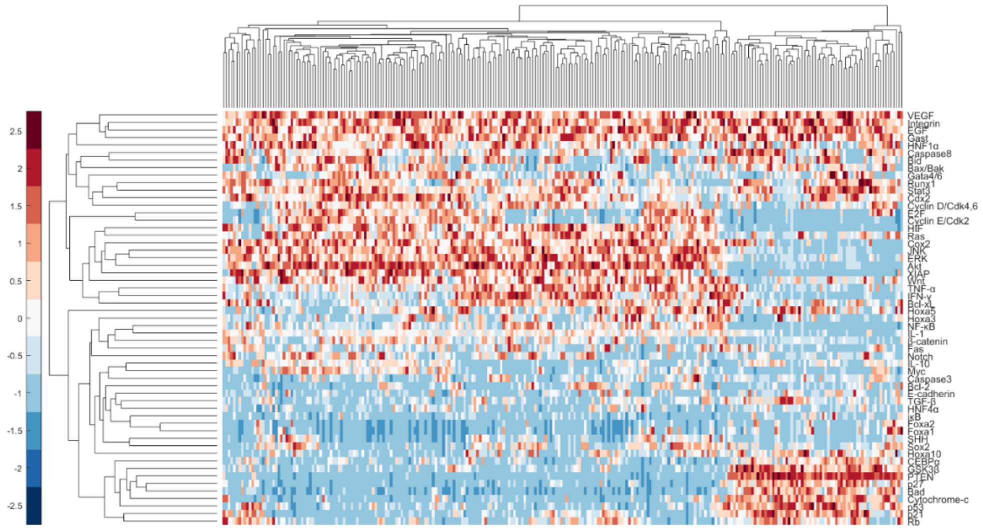

a): Heatmap illustrating the clustering of steady-states obtained from network self-emergence

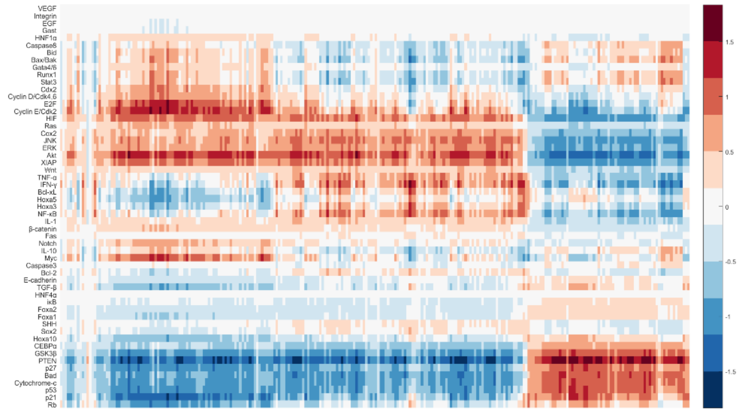

b): Heatmap depicting the steady-state after dimension reduction using PCA

Figure S2: A comparison of steady-state dimension reduction before and after PCA analysis. The x-axis represents different steady states, while the y-axis represents genes.

K-Means clustering was used to obtain expression profiles for center points of each steady state type (see Table S3).

Table S3: Expression profiles of the 55 endogenous agents corresponding to the centroids of each attractor obtained from K-Means clustering.

	a1	a2	a3
**Cyclin D/Cdk4,6**	0.812341	0.382667	0.564437
**Cyclin E/Cdk2**	1	0.178585	0.724772
**Myc**	0.923107	0.409748	0.516937
**E2F**	0.996777	0.31111	0.604813
**p21**	0	0.915668	0.18215
**p27**	0	0.95058	0.128899
**p53**	0.002957	0.981622	0.095427
**Rb**	0.075054	0.703766	0.367134
**Caspase3**	0.510212	0.563246	0.433403
**Cytochrome-c**	0.040945	1	0.067425
**Caspase8**	0.549696	0.655466	0.331908
**XIAP**	0.9362	0.041092	0.892168
**Bcl-2**	0.409545	0.411512	0.606767
**Bcl-xL**	0.269538	0.38211	0.661863
**Bid**	0.670132	0.594254	0.37311
**Bad**	0.077672	1	0.05084
**Bax/Bak**	0.649011	0.668195	0.30115

**Fas**	0.497222	0.441271	0.560644
**PTEN**	0	1	0
**Integrin**	0.455581	0.55193	0.454741
**E-cadherin**	0.332151	0.598732	0.428832
**β-catenin**	0.714726	0.349945	0.615368
**SHH**	0.472596	0.415052	0.591892
**Notch**	0.742841	0.410748	0.548078
**Wnt**	0.682659	0.264684	0.708412
**VEGF**	0.509332	0.500135	0.498197
**Cox2**	0.745515	0.148063	0.816636
**Ras**	0.7495	0.295632	0.664789
**JNK**	0.824193	0.040576	0.912682
**Akt**	1	0	1
**EGF**	0.431092	0.542084	0.469195
**HIF**	0.955851	0.035836	0.894044
**ERK**	0.835236	0.095996	0.853952
**Gast**	0.430979	0.536357	0.47508
**NF-κB**	0.517957	0.178804	0.825756
**iκB**	0.4224	0.609983	0.401205
**TNF-α**	0.52417	0.21405	0.788549
**IL-10**	0.636556	0.607003	0.366045
**IL-1**	0.639242	0.273246	0.70739
**TGF-β**	0.177165	0.638182	0.416083
**IFN-γ**	0.438092	0.158854	0.860439
**Stat3**	0.649172	0.655584	0.314038
**GSK3β**	0.039154	1	0.069769
**CEBPα**	0.267183	0.82952	0.204058
**Cdx2**	0.781582	0.476979	0.473333
**Foxa1**	0.28065	0.652941	0.382502
**Foxa2**	0.313928	0.645028	0.384668
**Gata4/6**	0.668576	0.489704	0.480465
**HNF1α**	0.643459	0.438581	0.537306
**HNF4α**	0.470249	0.51848	0.486382
**Hoxa3**	0.334589	0.345824	0.687418
**Hoxa5**	0.205341	0.502736	0.549776
**Hoxa10**	0.194217	0.66491	0.385666
**Runx1**	0.660676	0.638364	0.329622
**Sox2**	0.417453	0.44712	0.568887

The data is linearly scaled to [0, 1], with the red-blue color scale indicating the magnitude of expression. The first column represents the factors in the network, while a1, a3, and a2 represent the centroids of the first, second, and third attractors, respectively.

2. Analysis of all samples from Ruijin Hospital.

In order to compare the results with the network prediction, the same group of samples was standardized using Z-score prior to dimensionality reduction. As depicted in Figure S3 (a), the original data exhibited noise, posing challenges in extracting meaningful information directly. Hence, Principal Component Analysis (PCA) was employed to reduce the dimensionality of the samples. Principal components (PC1, PC2) with a contribution rate exceeding 8% were selected. As demonstrated in the two plots of Figure S3, dimensionality reduction successfully preserved the key information from the original data while mitigating the impact of noise. Subsequent analysis was performed based on the expression profiles obtained after dimensionality reduction.

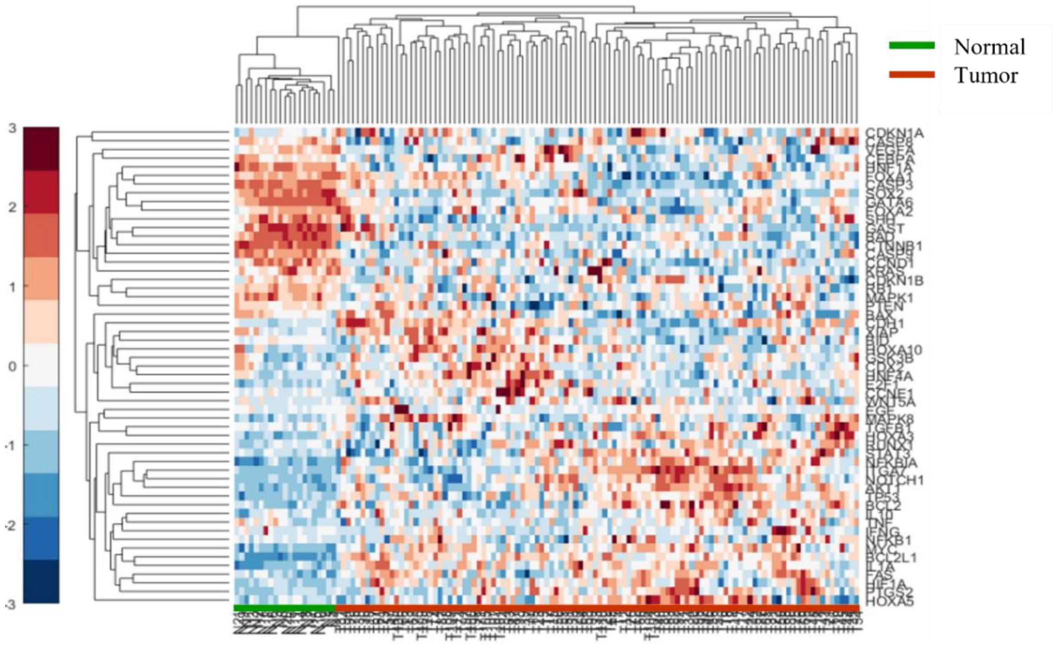

a): Hierarchical clustering of gastric cancer and normal samples

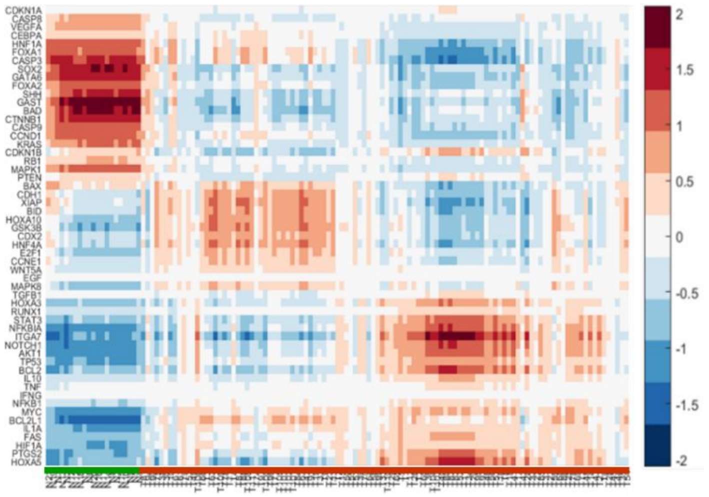

b): PCA dimensionality reduction results of gastric cancer and normal samples

Figure S3: Dimensionality reduction of different samples collected in the clinical setting. The x and y coordinates in the figure represent different samples and genes, respectively. The green color bar represents normal gastric mucosa samples, and the red color bar represents tumor samples, the same applies to subsequent figures.

Explore the classification of samples using hierarchical clustering and K-Means clustering methods. Next, hierarchical clustering was performed on the experimental data from Ruijin Hospital to cluster the samples. The clustering results are shown in main text Figure 4. The results indicate that using unsupervised hierarchical clustering, all samples from Ruijin Hospital were also clustered into three groups, with the normal samples forming one group and the abnormal tumor samples forming two types. This suggests that these tumor samples can be classified into two different subtypes of gastric cancer, which is consistent with the computational results obtained from the network constructed in this study.

The classification of samples was explored using hierarchical clustering and K-Means clustering

methods. Firstly, hierarchical clustering was performed on the experimental data obtained from Ruijin Hospital to group the samples. The clustering results are presented in Figure 4 of the main text. The findings indicate that through unsupervised hierarchical clustering, all samples from Ruijin Hospital were effectively clustered into three groups. Specifically, the normal samples formed one distinct group, while the abnormal tumor samples were divided into two subtypes. This observation suggests that these tumor samples can be classified into two different subtypes of gastric cancer, which aligns with the computational results obtained from the constructed network in this study.

Then, K-Means clustering is performed on the samples to validate the results of hierarchical clustering, as shown in Figure 4B(Main text). The expression profiles of each cluster center are listed in Table S4. Comparing the expression profiles of each cluster obtained from hierarchical clustering (Main text Figure 4A) with that obtained from K-Means clustering (Table S4), it is evident that the leftmost cluster from hierarchical clustering, which represents the normal samples, is consistent with the expression profile of the third cluster center obtained from K-Means clustering. The middle and rightmost clusters from hierarchical clustering, representing tumor samples, are consistent with that of the first and second cluster centers from K-Means clustering respectively. Thus the chip data from gastric cancer and normal gastric mucosa samples from Ruijin Hospital can be divided into three major groups (attractors). One represents the normal samples, and two from tumor samples map to the two subtypes of gastric cancer.

Subsequently, K-Means clustering was conducted on the samples to validate the results obtained from hierarchical clustering. The outcomes of K-Means clustering are illustrated in Figure 4B(Main text). The expression profiles of each cluster center are provided in Table S4. By comparing the expression profiles of each cluster derived from hierarchical clustering (Figure 4A) with those obtained from K-Means clustering (Table S4), it becomes evident that the leftmost cluster from hierarchical clustering, representing the normal samples, corresponds to the expression profile of the third cluster center obtained from K-Means clustering. Likewise, the middle and rightmost clusters from hierarchical clustering, representing tumor samples, correspond to the first and second cluster centers from K-Means clustering, respectively. Consequently, the chip data obtained from gastric cancer and normal gastric mucosa samples at Ruijin Hospital can be categorized into three major groups (attractors). One group represents the normal samples, while the other two groups, derived from tumor samples, correspond to the two subtypes of gastric cancer.

K-Means clustering was used to obtain expression profiles for center points of each samples type (see Table S4).

Table S4: Expression profiles corresponding to the centroids in the samples via K-means clustering.

	S1	S2	S3
**CCND1**	1	0.243971	0.510167
**CCNE1**	0.3385	0.380601	0.724192
**MYC**	0	0.715975	0.594914
**E2F1**	0.15803	0.448054	0.747377
**CDKN1A**	0.51142	0.544928	0.442855
**CDKN1B**	0.705598	0.626856	0.243051
**TP53**	0.022652	0.758515	0.470675
**RB1**	0.776091	0.412786	0.446446
**CASP3**	1	0.097422	0.576625
**CASP9**	1	0.296725	0.408527
**CASP8**	0.817113	0.444988	0.38738
**XIAP**	0.368035	0.252075	0.85336
**BCL2**	0.173356	0.857348	0.275713
**BCL2L1**	0	0.625806	0.761097

**BID**	0.327915	0.291901	0.830413
**BAD**	1	0.367792	0.186171
**BAX**	0.620236	0.268263	0.695958
**FAS**	0.076902	0.70063	0.506267
**PTEN**	0.712012	0.523151	0.35688
**ITGA7**	0.005616	1	0.179697
**CDH1**	0.31693	0.322892	0.801398
**CTNNB1**	1	0.339587	0.290346
**SHH**	1	0.399362	0.252043
**NOTCH1**	0	0.883118	0.371901
**WNT5A**	0.263096	0.493579	0.637913
**VEGFA**	0.828017	0.370038	0.46619
**PTGS2**	0.049952	0.800871	0.407684
**KRAS**	0.966577	0.371815	0.387766
**MAPK8**	0.146799	0.475043	0.723026
**AKT1**	0	0.860131	0.384901
**EGF**	0.410982	0.532323	0.51251
**HIF1A**	0.039635	0.670165	0.561299
**MAPK1**	0.988827	0.333954	0.418344
**GAST**	1	0.283262	0.244801
**NFKB1**	0.229789	0.594561	0.541996
**NFKBIA**	0	0.880029	0.376097
**TNF**	0.426477	0.576666	0.45378
**IL10**	0.302776	0.678233	0.407051
**IL1A**	0.02175	0.64386	0.600932
**TGFB1**	0.536404	0.552672	0.420313
**IFNG**	0.417655	0.512894	0.530818
**STAT3**	0.183271	0.817774	0.315033
**GSK3B**	0.099084	0.380693	0.856119
**CEBPA**	0.705448	0.350615	0.555765
**CDX2**	0.271017	0.378374	0.763927
**FOXA1**	1	0.155888	0.602871
**FOXA2**	1	0.287483	0.449373
**GATA6**	1	0.24089	0.359273
**HNF1A**	1	0.199564	0.52174
**HNF4A**	0.277628	0.298705	0.850445
**HOXA3**	0.172308	0.759048	0.38754
**HOXA5**	0.146412	0.893219	0.249976
**HOXA10**	0.167958	0.382244	0.816381
**RUNX1**	0.281883	0.600319	0.506751
**SOX2**	1	0.298416	0.275932

The data is linearly scaled to [0, 1] and represented by a red-blue gradient indicating high/low expression levels. The first column represents the gene, while S1, S3, and S2 represent the centroids of the first, second, and third attractor basins, respectively.

The expression profiles of the attractor centers computed by the network model were compared with the expression profiles of the attractor centers obtained from the samples collected at Ruijin Hospital. The comparison revealed conformity rates of 96.36%, 60.00%, and 89.09% for the three attractors, as presented in Table S5. It is important to consider several factors in evaluating the degree of agreement between the model and clinical data at the molecular level. Firstly, the simplicity of the core network, which consists of only 55 nodes, highlights the robustness and efficiency of the model. Secondly, it is crucial to acknowledge potential errors in the measurement of gene expression data, as these can introduce variability in the results. Lastly, the presence of heterogeneity among cancer samples further emphasizes the need for careful interpretation of the findings. Given these considerations, the level of concordance observed between the model and clinical data warrants further simulation and analysis. This approach can serve as a valuable "dry experiment" platform for exploring molecular-targeted therapy in cancer, significantly reducing the search space for subsequent wet experiments.

Table S5: A comparison of self-emerged steady states of the network to the gastric cancer and normal samples collected from Ruijin Hospital at the molecular level.

Notes	a1	s3	|a1-s3|	a2	s2	|a2-s2|	a3	s1	|a3-s1|
Cyclin D/Cdk4,6	0.812341	0.510167	0.302174	0.382667	0.243971	0.138696	0.564437	1	0.435563

Cyclin E/Cdk2	1	0.724192	0.275808	0.178585	0.380601	0.202016	0.724772	0.3385	0.386272
Myc	0.923107	0.594914	0.328193	0.409748	0.715975	0.306228	0.516937	0	0.516937
E2F	0.996777	0.747377	0.2494	0.31111	0.448054	0.136944	0.604813	0.15803	0.446783
p21	0	0.442855	0.442855	0.915668	0.544928	0.37074	0.18215	0.51142	0.32927
p27	0	0.243051	0.243051	0.95058	0.626856	0.323724	0.128899	0.705598	0.576698
p53	0.002957	0.470675	0.467718	0.981622	0.758515	0.223107	0.095427	0.022652	0.072774
Rb	0.075054	0.446446	0.371392	0.703766	0.412786	0.29098	0.367134	0.776091	0.408957
Caspase3	0.510212	0.576625	0.066412	0.563246	0.097422	0.465824	0.433403	1	0.566597
Cytochrome-c	0.040945	0.408527	0.367583	1	0.296725	0.703275	0.067425	1	0.932575
Caspase8	0.549696	0.38738	0.162317	0.655466	0.444988	0.210478	0.331908	0.817113	0.485204
XIAP	0.9362	0.85336	0.08284	0.041092	0.252075	0.210983	0.892168	0.368035	0.524132
Bcl-2	0.409545	0.275713	0.133832	0.411512	0.857348	0.445836	0.606767	0.173356	0.433411
Bcl-xL	0.269538	0.761097	0.49156	0.38211	0.625806	0.243697	0.661863	0	0.661863
Bid	0.670132	0.830413	0.160281	0.594254	0.291901	0.302353	0.37311	0.327915	0.045195
Bad	0.077672	0.186171	0.108499	1	0.367792	0.632208	0.05084	1	0.94916
Bax/Bak	0.649011	0.695958	0.046947	0.668195	0.268263	0.399933	0.30115	0.620236	0.319085
Fas	0.497222	0.506267	0.009045	0.441271	0.70063	0.25936	0.560644	0.076902	0.483742
PTEN	0	0.35688	0.35688	1	0.523151	0.476849	0	0.712012	0.712012
Integrin	0.455581	0.179697	0.275884	0.55193	1	0.44807	0.454741	0.005616	0.449125
E-cadherin	0.332151	0.801398	0.469247	0.598732	0.322892	0.27584	0.428832	0.31693	0.111902
β-catenin	0.714726	0.290346	0.42438	0.349945	0.339587	0.010357	0.615368	1	0.384632
SHH	0.472596	0.252043	0.220554	0.415052	0.399362	0.015689	0.591892	1	0.408108
Notch	0.742841	0.371901	0.370941	0.410748	0.883118	0.47237	0.548078	0	0.548078
Wnt	0.682659	0.637913	0.044745	0.264684	0.493579	0.228895	0.708412	0.263096	0.445316
VEGF	0.509332	0.46619	0.043142	0.500135	0.370038	0.130097	0.498197	0.828017	0.32982
Cox2	0.745515	0.407684	0.337831	0.148063	0.800871	0.652808	0.816636	0.049952	0.766684
Ras	0.7495	0.387766	0.361734	0.295632	0.371815	0.076183	0.664789	0.966577	0.301789
JNK	0.824193	0.723026	0.101168	0.040576	0.475043	0.434468	0.912682	0.146799	0.765883
Akt	1	0.384901	0.615099	0	0.860131	0.860131	1	0	1
EGF	0.431092	0.51251	0.081418	0.542084	0.532323	0.00976	0.469195	0.410982	0.058213
HIF	0.955851	0.561299	0.394552	0.035836	0.670165	0.634329	0.894044	0.039635	0.85441
ERK	0.835236	0.418344	0.416891	0.095996	0.333954	0.237958	0.853952	0.988827	0.134875
Gast	0.430979	0.244801	0.186178	0.536357	0.283262	0.253095	0.47508	1	0.52492
NF-κB	0.517957	0.541996	0.024039	0.178804	0.594561	0.415757	0.825756	0.229789	0.595967
iκB	0.4224	0.376097	0.046303	0.609983	0.880029	0.270046	0.401205	0	0.401205
TNF-α	0.52417	0.45378	0.07039	0.21405	0.576666	0.362616	0.788549	0.426477	0.362072
IL-10	0.636556	0.407051	0.229505	0.607003	0.678233	0.071231	0.366045	0.302776	0.063268
IL-1	0.639242	0.600932	0.038311	0.273246	0.64386	0.370614	0.70739	0.02175	0.685639
TGF-β	0.177165	0.420313	0.243148	0.638182	0.552672	0.08551	0.416083	0.536404	0.120321
IFN-γ	0.438092	0.530818	0.092727	0.158854	0.512894	0.35404	0.860439	0.417655	0.442784
Stat3	0.649172	0.315033	0.334139	0.655584	0.817774	0.162189	0.314038	0.183271	0.130766
GSK3β	0.039154	0.856119	0.816965	1	0.380693	0.619307	0.069769	0.099084	0.029315
CEBPα	0.267183	0.555765	0.288582	0.82952	0.350615	0.478905	0.204058	0.705448	0.50139
Cdx2	0.781582	0.763927	0.017656	0.476979	0.378374	0.098605	0.473333	0.271017	0.202316
Foxa1	0.28065	0.602871	0.322221	0.652941	0.155888	0.497053	0.382502	1	0.617498
Foxa2	0.313928	0.449373	0.135444	0.645028	0.287483	0.357546	0.384668	1	0.615332
Gata4/6	0.668576	0.359273	0.309303	0.489704	0.24089	0.248813	0.480465	1	0.519535
HNF1α	0.643459	0.52174	0.121719	0.438581	0.199564	0.239017	0.537306	1	0.462694
HNF4α	0.470249	0.850445	0.380196	0.51848	0.298705	0.219775	0.486382	0.277628	0.208754
Hoxa3	0.334589	0.38754	0.052951	0.345824	0.759048	0.413225	0.687418	0.172308	0.51511
Hoxa5	0.205341	0.249976	0.044635	0.502736	0.893219	0.390483	0.549776	0.146412	0.403364
Hoxa10	0.194217	0.816381	0.622164	0.66491	0.382244	0.282666	0.385666	0.167958	0.217709
Runx1	0.660676	0.506751	0.153925	0.638364	0.600319	0.038044	0.329622	0.281883	0.047738
Sox2	0.417453	0.275932	0.14152	0.44712	0.298416	0.148704	0.568887	1	0.431113
Conformity rates			96.36%			89.09%			60.00%

The first column represents the 55 factors in the network. The second column displays the expression profile of the center for the first attractor domain, predicted by the model. The third column shows the expression profile of the attractor center for the second attractor, using samples from Ruijin Hospital. The fourth column represents the absolute difference between the second and third columns, with values greater than 0.5 marked in red (indicating non-matching) and values less than 0.5 marked in blue (indicating matching). The seventh and tenth columns represent the absolute differences in the expression profiles of two additional pairs of attractor centers between the two datasets. The last row represents the matching rates of each attractor at

the molecular level.

3. Analysis of gastric cancer and normal samples from TCGA Database.

The same method was adopted for analysis TCGA data, the final comparison results are shown here.

Table S6: A comparison of self-emerged steady states of the network to the gastric cancer and normal samples collected from TCGA data.

Notes	a1	c1	|a1-c1|	a2	c2	|a2-c2|	a3	c3	|a3-c3|
CCND1	0.812341	0.562448059	0.249892941	0.382667	0.221208671	0.161458329	0.564437	0.614541482	0.050104482
CCNE1	1	0.735503373	0.264496627	0.178585	0.311865721	0.133280721	0.724772	0.298002562	0.426769438
MYC	0.923107	0.574940833	0.348166167	0.409748	0.380557774	0.029190226	0.516937	0.479472536	0.037464464
E2F1	0.996777	0.634261135	0.362515865	0.31111	0.118196869	0.192913131	0.604813	0.586465415	0.018347585
CDKN1A	0	0.32665207	0.32665207	0.915668	0.289690547	0.625977453	0.18215	0.904833089	0.722683089
CDKN1B	0	0.355387149	0.355387149	0.95058	0.549623449	0.400956551	0.128899	0.672436013	0.543537013
TP53	0.002957	0.51183916	0.50888216	0.981622	0.330430608	0.651191392	0.095427	0.607429549	0.512002549
RB1	0.075054	0.845619906	0.770565906	0.703766	0.338776413	0.364989587	0.367134	0.119187173	0.247946827
CASP3	0.510212	0.673498919	0.163286919	0.563246	0.05423185	0.50901415	0.433403	0.576765577	0.143362577
CASP9	0.040945	0.302677982	0.261732982	1	0.42464457	0.57535543	0.067425	0.84035242	0.77292742
CASP8	0.549696	0.759790934	0.210094934	0.655466	0.178076203	0.477389797	0.331908	0.361175248	0.029267248
XIAP	0.9362	0.596981706	0.339218294	0.041092	0.472098776	0.431006776	0.892168	0.380409886	0.511758114
BCL2	0.409545	0.273099792	0.136445208	0.411512	0.69724928	0.28573728	0.606767	0.682877051	0.076110051
BCL2L1	0.269538	0.671252426	0.401714426	0.38211	0.072153671	0.309956329	0.661863	0.566848809	0.095014191
BID	0.670132	0.655691395	0.014440605	0.594254	0.06622025	0.52803375	0.37311	0.593682625	0.220572625
BAD	0.077672	0	0.077672	1	0.386294112	0.613705888	0.05084	1	0.94916
BAX	0.649011	0.327906036	0.321104964	0.668195	0.146730568	0.521464432	0.30115	1	0.69885
FAS	0.497222	0.654916001	0.157694001	0.441271	0.456936413	0.015665413	0.560644	0.307864609	0.252779391
PTEN	0	0.479714645	0.479714645	1	0.649168234	0.350831766	0	0.419752549	0.419752549
ITGA7	0.455581	0.259664553	0.195916447	0.55193	1	0.44807	0.454741	0.47178388	0.01704288
CDH1	0.332151	0.649397489	0.317246489	0.598732	0.042446251	0.556285749	0.428832	0.620232935	0.191400935
CTNNB1	0.714726	0.6864425	0.0282835	0.349945	0.298224385	0.051720615	0.615368	0.378884203	0.236483797
SHH	0.472596	0.416770024	0.055825976	0.415052	0.151273386	0.263778614	0.591892	0.876318773	0.284426773
NOTCH1	0.742841	0.534309597	0.208531403	0.410748	0.716264076	0.305516076	0.548078	0.291621029	0.256456971
WNT5A	0.682659	0.82806957	0.14541057	0.264684	0.513647757	0.248963757	0.708412	0.016113153	0.692298847
VEGFA	0.509332	0.626776726	0.117444726	0.500135	0.199118148	0.301016852	0.498197	0.537848136	0.039651136
PTGS2	0.745515	0.702995871	0.042519129	0.148063	0.592458041	0.444395041	0.816636	0.138892276	0.677743724
KRAS	0.7495	0.69582024	0.05367976	0.295632	0.418948926	0.123316926	0.664789	0.276680104	0.388108896
MAPK8	0.824193	0.812490343	0.011702657	0.040576	0.43798918	0.39741318	0.912682	0.0941787	0.8185033
AKT1	1	0.209221359	0.790778641	0	0.397645999	0.397645999	1	0.995168754	0.004831246
EGF	0.431092	0.637635416	0.206543416	0.542084	0.55545067	0.01336667	0.469195	0.260476832	0.208718168
HIF1A	0.955851	0.912819687	0.043031313	0.035836	0.366916896	0.331080896	0.894044	0.001457643	0.892586357
MAPK1	0.835236	0.661324239	0.173911761	0.095996	0.504355555	0.408359555	0.853952	0.263784404	0.590167596
GAST	0.430979	0.342000901	0.088978099	0.536357	0.248659761	0.287697239	0.47508	0.912795812	0.437715812
NFKB1	0.517957	0.557970591	0.040013591	0.178804	0.295798836	0.116994836	0.825756	0.566230479	0.259525521
NFKBIA	0.4224	0.375738494	0.046661506	0.609983	0.42916095	0.18082205	0.401205	0.731506947	0.330301947
TNF	0.52417	0.550667401	0.026497401	0.21405	0.35813121	0.14408121	0.788549	0.531002951	0.257546049
IL10	0.636556	0.47822275	0.15833325	0.607003	0.492529823	0.114473177	0.366045	0.536941108	0.170896108
IL1A	0.639242	0.879025465	0.239783465	0.273246	0.366135172	0.092889172	0.70739	0.050844148	0.656545852
TGFB1	0.177165	0.368179181	0.191014181	0.638182	0.583131794	0.055050206	0.416083	0.629350998	0.213267998
IFNG	0.438092	0.702452043	0.264360043	0.158854	0.226748455	0.067894455	0.860439	0.408251201	0.452187799
STAT3	0.649172	0.647148695	0.002023305	0.655584	0.398326448	0.257257552	0.314038	0.36212628	0.04808828
GSK3B	0.039154	0.842474101	0.803320101	1	0.330861841	0.669138159	0.069769	0.129543358	0.059774358
CEBPA	0.267183	0.346718144	0.079535144	0.82952	0.276856707	0.552663293	0.204058	0.88527467	0.68121667
CDX2	0.781582	0.594925008	0.186656992	0.476979	0.273840201	0.203138799	0.473333	0.528979675	0.055646675
FOXA1	0.28065	0.51348319	0.23283319	0.652941	0.213795736	0.439145264	0.382502	0.69071038	0.30820838
FOXA2	0.313928	0.373994561	0.060066561	0.645028	0.143528583	0.501499417	0.384668	0.943791406	0.559123406
GATA6	0.668576	0.344155462	0.324420538	0.489704	0.064544641	0.425159359	0.480465	1	0.519535
HNF1A	0.643459	0.511669709	0.131789291	0.438581	0.071158462	0.367422538	0.537306	0.798081145	0.260775145
HNF4A	0.470249	0.55836606	0.08811706	0.51848	0.049632054	0.468847946	0.486382	0.746441656	0.260059656
HOXA3	0.334589	0.375007008	0.040418008	0.345824	0.873466846	0.527642846	0.687418	0.406269751	0.281148249
HOXA5	0.205341	0.320065522	0.114724522	0.502736	0.921201494	0.418465494	0.549776	0.450571455	0.099204545
HOXA10	0.194217	0.787542368	0.593325368	0.66491	0.41571689	0.24919311	0.385666	0.146570129	0.239095871
RUNX1	0.660676	0.738768265	0.078092265	0.638364	0.292737617	0.345626383	0.329622	0.307334235	0.022287765
SOX2	0.417453	0.275771892	0.141681108	0.44712	0.597101236	0.149981236	0.568887	0.752565178	0.183678178

Conformity rates	90.10%	78.20%	70.90%

The first column represents the 55 factors in the network. The second column displays the expression profile of the center for the first attractor domain, predicted by the model. The third column shows the expression profile of the attractor center for the second attractor, using samples from TCGA. The fourth column represents the absolute difference between the second and third columns, with values greater than 0.5 marked in red (indicating non-matching) and values less than 0.5 marked in blue (indicating matching). The seventh and tenth columns represent the absolute differences in the expression profiles of two additional pairs of attractor centers between the two datasets. The last row represents the matching rates of each attractor at the molecular level.

4. Analysis of all samples from GEO Database.

We compared the calculated results of VEGF=0 with a set of experimental data from GEO Datasets (GSE160613), Gene expression data of human gastric cancer MKN45 cells xenograft tumors treated with anti-VEGFR2 and anti-VEGF-A antibodies, the final comparison results are shown here.

Table S7: A comparison of simulation results of anti-VEGF with GEO data.

	Simulate Results		GEO: GSE160613		
**Notes**	Cancer1/Normal	Cancer2/Normal	antiVEGFR2/NC	antiVEGFR2&VEGFA/NC	antiVEGFA/NC
**CCND1**	↑	↓	↑	↑	↑
**CCNE1**	↑	↑	↓	↑	↑
**MYC**	↑	↓	↑	↑	↑
**E2F1**	↑	↑	↑	↓	↓
**CDKN1A**	↓	↓	↑	↑	↑
**CDKN1B**	↓	↓	↓	↑	↑
**TP53**	↓	↓	↓	↑	↓
**RB1**	↓	↑	↑	↑	↓
**CASP3**	↓	↓	↓	↓	↑
**CASP9**	↓	↓	↓	↓	↓
**CASP8**	↓	↓	↑	↓	↓
**XIAP**	↑	↑	↓	↓	↓
**BCL2**	↑	↑	↓	↓	↓
**BCL2L1**	↑	↑	↓	↑	↑
**BID**	↓	↓	↓	↑	↑
**BAD**	↓	↓	↓	↑	↑
**BAX**	↓	↓	↑	↓	↑
**FAS**	↑	↑	↓	↓	↓
**PTEN**	↓	↓	↑	↓	↑
**ITGA7**	↓	↓	↓	↓	↓
**CDH1**	↓	↓	—	—	—
**CTNNB1**	↑	↑	↑	↓	↑
**SHH**	↑	↑	↓	↓	↓
**NOTCH1**	↑	↓	↑	↓	↓
**WNT5A**	↑	↑	↓	↓	↓
**VEGFA**	↓	↓	↑	↑	↓
**PTGS2**	↑	↑	—	—	—
**KRAS**	↑	↑	↓	↓	↓
**MAPK8**	↑	↑	↓	↓	↓
**AKT1**	—	—	↑	↑	↑
**EGF**	↓	↓	↓	↓	↓
**HIF1A**	↑	↑	—	—	—
**MAPK1**	↑	↑	↑	↑	↑
**GAST**	↓	↓	↓	↓	↓
**NFKB1**	↑	↑	↑	↑	↑
**NFKBIA**	↓	↓	↓	↓	↓
**TNF**	↑	↑	↓	↓	↓
**IL10**	↓	↓	↓	↓	↓
**IL1A**	↑	↑	↓	↓	↓
**TGFB1**	↓	↓	↓	↓	↓
**IFNG**	↑	↑	↓	↓	↓
**STAT3**	↓	↓	↓	↓	↓
**GSK3B**	↓	↓	↑	↓	↓
**CEBPA**	↓	↓	↓	↓	↑

**CDX2**	↑	↓	↓	↓	↓
**FOXA1**	↓	↑	↓	↓	↓
**FOXA2**	↓	↓	↑	↑	↓
**GATA6**	↑	↓	↑	↑	↑
**HNF1A**	↑	↓	↓	↓	↓
**HNF4A**	↑	↑	↓	↑	↑
**HOXA3**	↑	↑	↓	↓	↓
**HOXA5**	↓	↑	↓	↓	↓
**HOXA10**	↓	↓	↓	↑	↑
**RUNX1**	↓	↓	↓	↓	↓
**SOX2**	↑	↑	↓	↓	↓

Calculating the "pre-inhibition/post-inhibition" values for both the calculated VEGF=0 and the GEO dataset (GSE160613). If >1 is "↑", and <1 is "↓". The accuracy was 57.3%, significantly higher than the average concordance (~25%) of randomly generated numbers with the same dimensions in two columns.

D. Simulation results after perturb the network.

1: A typical simulation results, simulation_result.

2: Network intervention with VEGF resulted in a predicted steady state of the network, target:VEGF=0.

3: Network intervention with Caspase3 and NF-κB yielded the following predicted results, targets: Caspase3=1 & NF-κB=0.

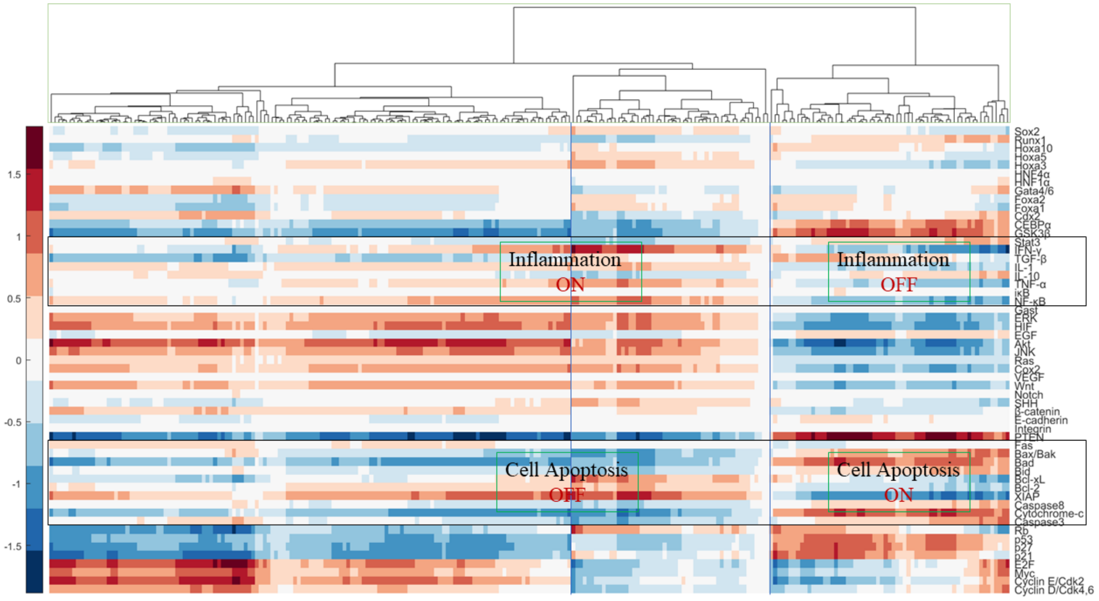

Figure S4 Hierarchical clustering heatmap of anti-VEGF treatment. After inhibiting VEGF, the left and middle clusters show apoptosis modules activated and growth factor modules opened, indicating that cells are still able to evade apoptosis and have unrestricted cell proliferation.

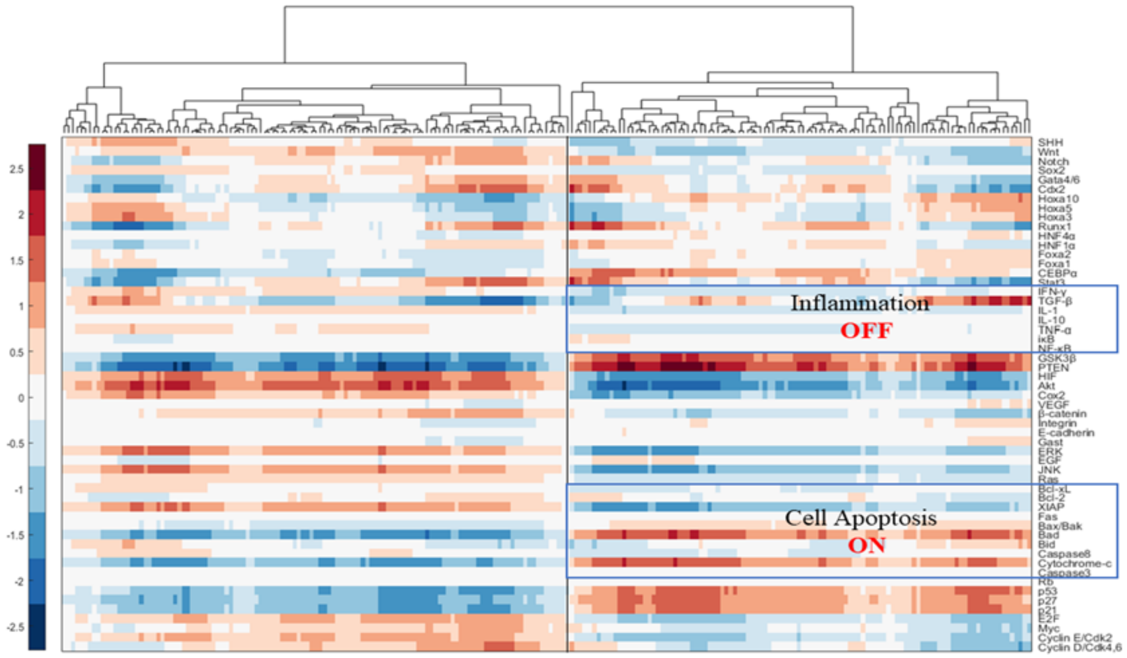

Figure S5 Hierarchical clustering heatmap of inhibiting Caspase3 and activating NF-κB. After inhibiting Caspase3 and activating NF-κB, the right cluster shows the apoptosis module open and the immune module closed, indicating that cells cannot evade apoptosis and have restricted cell proliferation, and the immune response is weakened.
